# Multi-gene analysis of the *Russula* crown clade (Russulales, Basidiomycota) revealed six new species and *Alboflavinae* subsect. nov. from Fagaceae forests in China

**DOI:** 10.3389/fpls.2024.1454035

**Published:** 2024-10-07

**Authors:** Caiyun Niu, Tiezhi Liu, Shiyi Zhao, Jing Ren, Yi Zhao, Xia Kang, Weiqiang Qin, Xuejiao Xie, Xu Zhang, Tiezheng Wei, Jinghua Tian, Xiao Li, Ming Li, Shoumian Li, Guojie Li

**Affiliations:** ^1^ College of Horticulture, Hebei Agricultural University, Baoding, Hebei, China; ^2^ Hebei Key Laboratory of Vegetable Germplasm Innovation and Utilization, Baoding, Hebei, China; ^3^ Collaborative Innovation Center of Vegetable Industry of Hebei Province, Baoding, Hebei, China; ^4^ College of Chemistry and Life Sciences, Chifeng University, Chifeng, China; ^5^ Jishou University, Zhangjiajie, Hunan, China; ^6^ State Key Laboratory of Mycology, Institute of Microbiology, Chinese Academy of Sciences, Beijing, China

**Keywords:** Agaricomycetes, ectomycorrhizal fungi, edible fungi, macrofungus, Russulaceae, taxonomy

## Abstract

**Introduction:**

The crown clade is one of two major groups in the *Russula* subg. *Russula*.

**Methods/material:**

An analysis of Chinese samples was performed based on the morphology, internal transcribed spacer (ITS) sequences, and multi-gene phylogenies of 28S nrLSU, 16S mtSSU, *rpb1*, *rpb2*, and *tef1-α*.

**Results:**

The results supported the independence of six new species: *Russula alboflava* (sect. *Amethystinae*), *R. chrysantha* (subsect. *Chamaeleontinae*), *R. liyui* (subsect. *Laricinae*), *R. lutescens* (subsect. *Olivaceinae*), *R. paraxerampelina*, and *R. prunicolor* (subsect. *Xerampelinae*) from Fagaceae forest habitats. Subsect. *Alboflavinae* was newly proposed in sect. *Amethystinae*. Members of the new subsection include *R*. *alboflava*, *R. burlinghamiae*, and possibly *R. ballouii*.

**Discussion:**

Our analyses also supported the claim that two species of *R. fulvograminea* (subsect. *Laricinae*) and *R. subrubens* (subsect. *Xerampelinae*) have a Eurasian distribution. The habitat and primary hosts of the main phylogenetic clades within related subsections were summarized and discussed.

## Introduction

*Russula* Pers. is the genus that contains the most numerous species in Russulales, with a total of approximately 750 to 900 known species widely scattered in tropical rainforests, temperate deciduous woodlands, subarctic tundra, and subalpine stands of coniferous trees (Kirk et al., 2008; Looney et al., 2016). The de facto number of species in the *Russula* genus may be as high as 2,000 (Adamčík et al., 2019). This genus is one of the most common and dominant members of the ectomycorrhizal (ECM) fungal communities in forest ecosystems (Buyck et al., 1996; Geml et al., 2009; Looney et al., 2016, 2018), of which Fagaceae is one of the main ECM plant lineages (Wang et al., 2017; Hackel et al., 2022). Many species of this genus are collected around the world as delicious and nutritious edible mushrooms (Yang and Pfister, 2006; Song et al., 2007; Buyck et al., 2008; Wang et al., 2009; Dai et al. 2010; Li 2010; Dugan, 2011; Li, 2014; Wang, 2019; Wang, 2020; Wei et al., 2021).

The conjoint morphological and molecular phylogenetic analyses of the *Russula* genus were initiated in the early years of this century. Most of the higher-level phylogenetic relationships in this group remain unsolved because only internal transcribed spacer (ITS) sequences of a limited number of species were analyzed (Eberhardt, 2002; Miller and Buyck, 2002; Li et al., 2019). Multi-gene analyses were carried out later to clarify the generic and intrageneric phylogenetic relationships of the Russulaceae and *Russulas*. These results strongly support the phylogenetic framework of the *Russula* genus (Buyck et al., 2008, 2018; Looney et al., 2016). The concept of a crown clade was proposed by Looney et al. (2016) and adopted in subsequent analyses (Buyck et al., 2018; Ghosh et al., 2021a; Song et al., 2021). This clade gained its name because of its uppermost topological location in the phylogram. It is characterized by being agaricoid and rarely secotioid or gasteroid basidiomata, with a variously colored pileus, equal lamellae, rare or absent lamellulae, a mostly mild and rarely acrid tasting context, a usually ochraceous to yellow spore print, a amyloid suprahilar spot, absent or present primordial hyphae, and generally narrow hyphal extremities in the suprapellis (Buyck et al., 2018). Members of this clade comprise five of the nine subgenera of *Russula* in Romagnesi (1985). In the infrageneric taxonomy of Sarnari (1998), the crown clade contains partial members of the subg. *Russula* Romagn. According to the classification scheme of Singer (1986), the partial species of sect. *Decolorantes*, sect. *Rigidae*, and sect. *Russula* are current members of the crown clade. Looney et al. (2016) regarded the crown clade as a subgeneric group in *Russula*. Buyck et al. (2018) processed the branch composed of the crown and *Russula* clades of Looney et al. (2016) as subg. *Russula* emend. Buyck & V. Hofst. The multi-gene phylogenetic analyses of the crown clade indicated a lack of support for most of the conclusions. This could be caused by the rapid spread of the subg. *Russula* as the dominant species in many regions of the Northern Hemisphere (Buyck et al., 2018). The high species diversity of the crown clade in the *Russula* genus adds a huge taxonomic complexity to this group (Looney et al., 2016; Buyck et al., 2018; Adamčík et al., 2019).

The high species diversity of the crown clade has been reflected in the taxonomy of *Russula* from China, the adjacent East, Southeast Asia, and the Himalayan regions in recent years. Although a total of 31 new species of *Russula* in the crown clade have been described from these areas during the past five years (Jabeen et al., 2020; Ghosh et al., 2021a, b, 2023; Hampe and Manz, 2021; Kiran et al., 2021; Song et al., 2021; Wang J. et al., 2021; Ji et al., 2022; Khurshid et al., 2022; Li and Bau, 2022; Zhou et al., 2022a, b, 2023; Liu et al., 2024), the actual number still remains unknown. Here, we describe six new species, including two new Chinese records of the *Russula* crown clade based on detailed morphology and multi-gene phylogeny. The relationships between forest type and phylogenetic topology of relevant infrageneric taxa are illustrated and discussed, with the goal of unraveling the specific diversity and evolutionary process of *Russula*.

## Materials and methods

### Specimen collection sites

Fresh basidiomata were collected from the Fagaceae forests in Hunan, Hebei, and Inner Mongolia during fungal forays from 2019 to 2023. The majority of the newly collected specimens in this analysis were from Taihang, Yanshan, and the southern Khingan Mountains, extending from Hebei to Inner Mongolia, which contains areas of deciduous coniferous forest, evergreen coniferous forest, intermixed broad-leaved and coniferous forest, and deciduous broad-leaved forest at altitudes ranging from 600 to 2500 m. Ectomycorrhizal hosts in this area include broad-leaved tree species *Betula platyphylla*, *Juglans mandshurica*, *Populus davidiana*, *Quercus mongolica*, *Q. wutaishansea*, and *Tilia mongolica*, as well as coniferous tree species *Abies nephrolepis*, *Picea meyeri*, *P. wilsonii*, *Pinus tabuliformis*, and *Platycladus orientalis* (Wang L. et al., 2021). A small number of specimens in this study were collected from Zhangjiajie City in Hunan Province, southern China. The main forest types in this region include low mountain coniferous forests, evergreen-deciduous forests, and montane elfin forests. The main ectomycorrhizae-forming broad-leaved tree species are *Castanopsis sclerophylla*, *Lithocarpus glaber*, *Quercus engleriana*, *Q. phanera*, *Q. multinervis*, and coniferous trees of *Pinus massoniana* (Qi, 1990; Luo et al., 2009). There were 50 new collections from Taihang, Yanshan, and the Greater Khingan Mountains, as well as three from the Wuling Mountains in Zhangjiajie City. Detailed information on sampling sites is available in **Supplementary Table 1**.

### Morphology

Basidiome pictures were taken with Nikon E7900 and D3100 digital cameras. Macro-morphological characters were recorded under daylight following the color standards of Ridgway (1912). The spore print color followed the standards of Romagnesi (1985). The newly collected samples were dehydrated using a Fruit LT-21 electric food drier at 60–70°C for 12 h. Dried specimens were preserved in the mycological herbarium of Chifeng University (CFSZ), the herbarium of Hebei Agricultural University (HBAU), and the Herbarium of the Mycology Institute of Microbiology, Chinese Academy of Sciences (HMAS). The herbarium abbreviation codes used in this analysis followed those of the Index Herbariorum (https://sweetgum.nybg.org/science/ih, accessed in 2024).

The morphological terminologies in previous studies were used in our descriptions (Romagnesi, 1985; Sarnari, 1998; Adamčík et al., 2019). The morphological observations for the new species were based on corresponding specimen aggregations. The microscopic characters of the specimens were observed in small pieces of rehydrated tissue using a Nikon Eclipse 80i microscope and imaged with a Cossim U3CCD06000KPA camera. Anticlinal sections were created manually with a Jianyu stainless blade. Rehydration was accomplished by immersion in 5% KOH. Microscopic illustrations were made after staining with Congo red. Melzer’s reagent was used to detect the amyloid reaction of the basidiospore ornamentations. The size range of the basidiospores was presented in the form of (W–) X–Y (–Z), where W and Z are the minimum and maximum values of basidiospore width and length respectively. X–Y corresponds to the 95% size range after the exclusion of the 5% minimum and maximum values. The number of basidiospores, basidiomata, and specimens used for the measurement data is shown in the form *a/b/c*. At least 20 targets were observed and measured for each microscopic structure. The Q values in plain font are the size ranges of the basidiospore length/width ratio, and Q values in bold are the means and standard deviations of the basidiospore length/width ratio. The length of ornamentations and sterigma was excluded in the measurements of basidiospores and basidia. Sulfovanillin (SV) was used to examine the color changes of the cystidium contents in the hymenium and pileipellis. Line drawings were created from optical microscope photos with a Wacom CTL-671 pen tablet. High-magnification detailed microscopic morphological characters of basidiospores were observed and photographed with a Hitachi-SU8010 field-emission scanning electron microscope (SEM).

### DNA isolation, PCR, and sequencing

Whole genomic DNA was extracted from dried *Russula* specimens using an optimized CTAB method (Li, 2014), and the ITS1-5.8S-ITS2 ribosomal DNA region (ITS) was amplified with ITS5/ITS4 primers (White et al., 1990). The partial large subunit of nuclear ribosomal DNA region (28S nrLSU) was amplified with LROR/LR5 primers (Vilgalys and Hester, 1990), and the small subunit mitochondrial DNA region (16S mtSSU) was amplified with MS1/MS2 primers (White et al., 1990). The genes (*rpb1* and *rpb2*) for the largest and second-largest subunits of RNA polymerase were amplified with the rpb1-Af/rpb1-Cr and brpb2-6f/frpb2-7cR primer pairs (Liu et al., 1999; Matheny et al., 2002; Matheny, 2005). The translation elongation factor 1 (*tef1-α*) gene was amplified using EF-983F/EF-1567R (Rehner and Buckley, 2005). The amplification reaction contained 21 μL of ddH_2_O, 25 μL of PCR mix (Beijing Catascis Biotech Co., Ltd.), 1.5 μL of both forward and reverse primers (10 μmol/L, Shanghai Sangon Biotech Co., Ltd.), and 1 μL of DNA template (ca. 20 ng). The PCR parameters were as follows: initial denaturation at 95°C for 5 min, 35 cycles of 55 s at 95°C for denaturation, 55 s at 55°C for annealing, 55 s at 95°C for extension, and a final extension at 72°C for 10 min. Touchdown PCR conditions were set for *rpb1*, *rpb2*, and *tef1-α* gene amplifications as described in the references above. Annealing temperatures were lowered from 66°C to 56°C by 1°C per cycle over the first 10 cycles, with a 1.5 min extension time per cycle at 72°for the *tef1-α* gene. A 0.3 s ramp from annealing to extension step per cycle was set for the *rpb1* and *rpb2* genes. The PCR products were detected using 1.5% agarose gel electrophoreses, and a Sangon EZ-10 PCR product spin column purification kit was used to remove impurities. DNA sequencing was performed using an Applied Biosystems 3730xl DNA analyzer from the Suzhou GeneWiz Biotechnology Co., Ltd. An Applied Biosystems BigDye Terminator v3.1 kit was used in the sequencing operations with the same primer pairs as for the PCR procedure. Eligible DNA sequences were submitted to the GenBank database (https://www.ncbi.nlm.nih.gov/genbank, accessed on June 2024). The accession numbers of the newly generated sequences are listed in bold type in **Supplementary Tables 2**-**6**.

### ITS and multi-gene phylogeny analyses

The newly acquired sequences in this study were initially aligned with sequences in GenBank using the BLAST nucleotide comparison tool. Sequences derived from forward and reverse primers were aligned and spliced using SeqMan Pro from Lasergene 7.7.0 (DNASTAR, Inc). A total of 599 referential sequences were retrieved from the GenBank and UNITE (https://unite.ut.ee, accessed in 2024) databases, including 270 ITS sequences and 329 from other genes. The ITS sequences were cited as corresponding to *Russula* infrageneric groups from preliminary phylogenetic analyses (Eberhardt 2002; Miller and Buyck, 2002; Vidal et al., 2002, 2019; Whitbeck, 2003; Durall et al., 2006; Palmer et al., 2008; Kranabetter et al., 2009; Schoch et al., 2012; Geml and Taylor, 2013; Li et al., 2013, 2018a, 2018b; Osmundson et al., 2013; Li, 2014, 2021; Guo et al., 2014; Suz et al., 2014; Adamčík et al., 2016a; Malysheva et al., 2016; Mua et al., 2016; Rosenblad et al., 2016; Bazzicalupo et al., 2017; Jabeen et al., 2017; Jiang 2017; Jiang et al., 2017; Liu et al., 2017; Katanić et al., 2019; Leonhardt et al., 2019; Xu et al., 2019; Kiran et al., 2021; Motiejūnaitė et al., 2021; Shi, 2021; Sleiman et al., 2021; Trendel, 2021; Shirakawa et al., 2022; Zhou et al., 2022a, 2022b). Representative lineages of previous multi-gene phylogenetic analyses were consulted for the sampling of the referential sequences (Calonge and Martín, 2000; Eberhardt 2002; Schoch et al., 2012; Looney et al., 2016; Bazzicalupo et al., 2017; Caboň et al., 2017, 2019; Buyck et al., 2018; Crous et al., 2019; Vidal et al., 2019; Zhou et al., 2022a; Kiran et al., 2021; Song et al., 2021; Li et al., 2023). Sequences of the same genes were gathered and multi-aligned using Mafft 7.520 with the G-INS-i strategy (Katoh and Standly, 2016). Unevenly or mistakenly aligned sites at the beginning and end were manually adjusted using Bioedit 7.1.3.0 (Hall, 1999). Phylogenetic analyses of new species and closely related lineages were carried out based on ITS regions. The DNA matrices of nrLSU, mtSSU, *rpb1*, *rpb2*, and *tef-1α* were concatenated using SequenceMatrix 1.7.8 (Vaidya et al., 2011) for multi-gene analyses. Maximum likelihood (ML) analyses were performed with raxmlGUI 1.3 (Edler et al., 2020). The ML + rapid bootstrap setting and GTRGAMMAI model were used with 1,000 bootstrap iterations (Felsenstein, 1985). Bayesian analyses were carried out using MrBayes 3.2.7 (Ronquist and Huelsenbeck, 2003; Ronquist et al., 2012). The substitution models for Bayesian analyses of each gene were calculated by MrModeltest 2.3 through the Akaike information criterion using PAUP^*^ 4.0 (Swofford 2004; Nylander, 2004; Liddle, 2007). A Markov chain Monte Carlo simulation was run for 4.0 × 10^6^ generations, with the sampling frequency set to every 100th generation. The calculation was stopped when the standard deviation of the split frequencies stably fell below 0.01. The first 25% of the sampling trees were discarded for the burn-in phase of each analysis. Node and branch parameters were summarized and written to the consensus tree. Bayesian posterior probabilities were calculated based on the remaining 75% of the trees. The calculation convergences were evaluated using Tracer 1.7 (Rambaut et al., 2018). Phylogenetic trees were presented in FigTree 1.4.4 (http://tree.bio.ed.ac.uk/, accessed on 22 April 2024). Annotations for taxon names in the phylograms were added using Adobe Illustrator 16.0.0 and Adobe Photoshop 10.0.

## Results

### Multi-gene phylogeny

**Figure 1:**
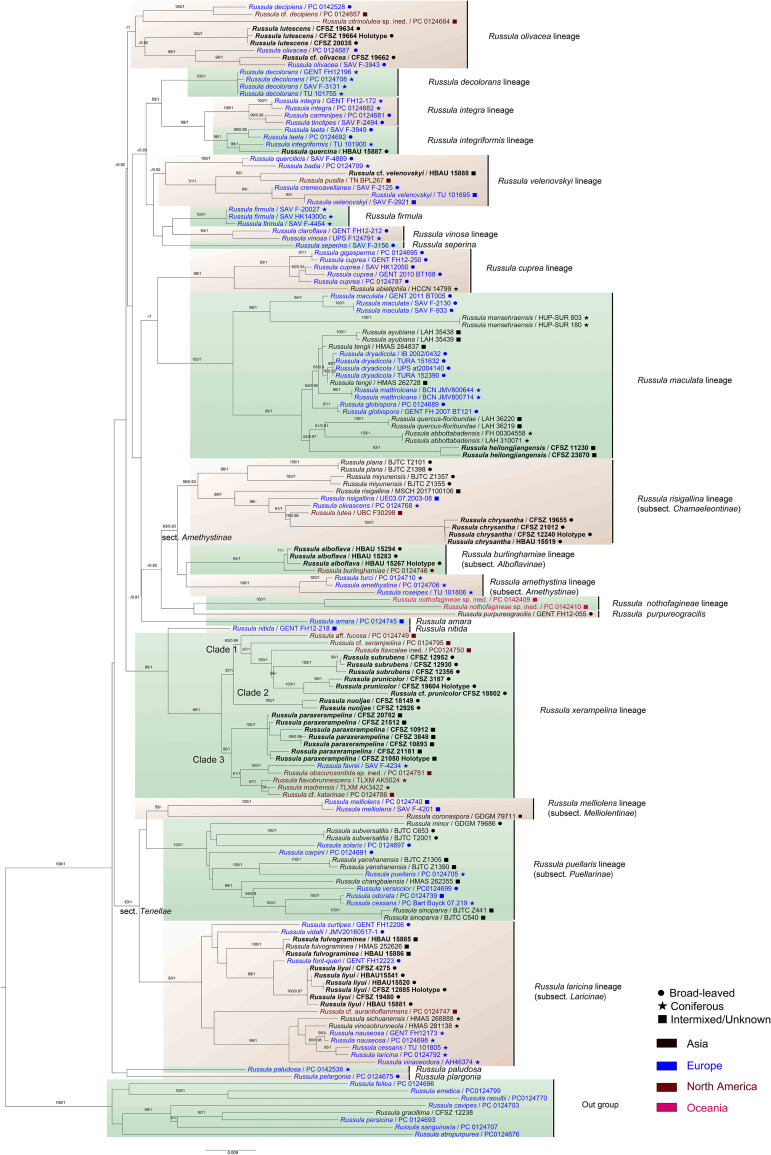
Maximum likelihood (ML) phylogenetic tree of the *Russula* crown clade combining nrLSU, mtSSU, *rpb1*, *rpb2*, and *tef1-α* sequences. Bootstrap values (MLBS) ≥75% of ML and posterior probabilities (PP) of Bayesian analysis ≥0.9 are presented above the clades as MLBS/PP. Labels in bold represent new collections for this analysis.

Sequence similarities in the ITS regions of the new species and the new Chinese records were evaluated through BLAST searches in the GenBank and UNITE databases (**Supplementary Table 7**). The 3,613 bp matrix for multi-gene phylogenetic analyses contains 878 bp of nrLSU, 492 bp of mtSSU, 995 bp of *rpb1*, 703 bp of *rpb2*, and 544 bp of *tef-1α* DNA. The best nucleotide substitution model for the Bayesian analysis is GTR+I+G for nrLSU and mtSSU, and SYM+I+G for *rpb1*, *rpb2*, and *tef-1α*. The matrix includes 734 sequences, of which 183 are newly sequenced. Sequences of the matrix correspond to 17 lineages and seven species, as well as eight species of subg. *Russula* as an out-group. Phylogenetic analyses of locus combinations for the *Russula* crown clade indicate a similar topology to those of Looney et al. (2016); Buyck et al. (2018), and Adamčík et al. (2019). Only the phylogenetic topology of the ML analysis is presented in **Figure 1** based on the consistency of the basal ranks in the BI, ML, and MP trees. The phylograms indicated that all of the new species formed highly supported clades in the multi-gene analyses.

The close relationship between *R. alboflava* and *R. burlinghamiae* was supported (MLBS 94, BSPP 1). The *Russula alboflava* lineage clusters with that of *R. amethystina*, forming a clade that is not highly supported. The *R. chrysantha* clade closely nests with those of samplings identified as *R. risigallina*, *R. lutea*, and *R. olivascens* (MLBS). The strength of the phylogenetic position of *R. fulvograminea* in subsect. *Laricinae* (Romagn.) Bon still remains unconfirmed by multi-gene phylogeny. The close relationship (MLBS) and obvious genetic distance between *R. liyui* and *R. font-queri* were revealed. The *R. lutescens* clade clustered with samples identified as *R. olivacea*, *R*. cf. *olivacea* with possible support of Bayesian analysis (MLBS). The *R. paraxerampelina* clade formed a strongly supported clade with specimens of *R.* cf. *katarinae*, *R. favrei*, *R. flavobrunnescens*, *R. obscurosordida*, and *R. madrensis* (MLBS). Clades of *R. prunicolor* and *R. subrubens* closely nested with those of *R*. aff. *fucosa*, *R*. cf. *xerampelina*, and *R. tlaxcalae* ined. (MLBS). The *R. chrysantha* clade was supported by ML analysis.

### ITS region phylogeny

A total of 76 sequences, including 11 newly sequenced ones, were included in the ITS region matrix of sect. *Amethystinae*. These sequences corresponded to two species and five complexes in this section, as well as four species in an out-group from sect. *Polychromae* (Maire) Sarnari. The matrix was 613 bp in length, and there were 139 bp of ITS-1, 168 bp of 5.8S, and 251 bp of ITS-2 in the database. Of the 613 total characters in the ITS matrix of sect. *Amethystinae*, 373 were constant, 58 variable characters were parsimony uninformative, and 182 were parsimony informative. The tree had a CI of 0.636, an RI of 0.871, an RC of 0.553, an HI of 0.364, and a TL of 472. The best substitution model selected for Bayesian analysis of sect. *Amethystinae* was SYM+I+G. The *R. alboflava* clade, which contains three specimens from Japan, can be significantly distinguished from known species (**Supplementary Figure 1**), as it clustered with samples of *R. burlinghamiae* with strong support (MLBS, BSPP). The *R. chrysantha* clade was supported by ML analysis with a bootstrap value of 60, and northern European samples were also included in this clade.

A total of 105 sequences, including 20 newly sequenced ones, were found in the ITS region matrix of subsect. *Laricinae*. These sequences corresponded to seven species and nine complexes in this subsection, as well as four species in an out-group from subsect. *Maculatinae* (Romagn.) Konrad & Joss. The matrix was 575 bp in length, and there were 138 bp of ITS-1, 167 bp of 5.8S and 274 bp of ITS-2 in the database. Of the 575 total characters in subsect. *Laricinae* ITS matrix, 350 were constant, 36 variable characters were parsimony-uninformative, and 189 were parsimony-informative. The tree had a CI of 0.465, an RI of 0.858, an RC of 0.399, an HI of 0.535, and a TL of 688. The best substitution model selected for Bayesian analysis of subsect. *Laricinae* was HKY+I+G. *Russula liyui* cannot be clearly distinguished from *R. font-queri* in ITS phylogenetic analyses (**Supplementary Figure 2**). European and Asian samplings of *R*. *fulvograminea* formed a fully supported clade (MLBS, BSPP).

A total of 55 sequences, including four newly sequenced ones, were involved in the ITS region matrix of subsect. *Olivaceinae*. The long insertions in the ITS region of this subsection were removed manually. These sequences corresponded to two species and three complexes in this subsection, as well as four species in an out-group from subsect. *Xerampelinae*. The matrix was 609 bp in length, including 136 bp of ITS-1, 167 bp of 5.8S, and 258 bp of ITS-2 in the database. Of 609 total characters in the ITS matrix of subsect. *Olivaceinae*, 437 were constant, 44 variable characters were parsimony uninformative, and 128 were parsimony informative. The tree had a CI of 0.679, an RI of 0.921, an RC of 0.625, an HI of 0.321, and a TL of 308. The best substitution model selected for Bayesian analysis of subsect. *Olivaceinae* was SYM+I+G. The fully supported clade of *R. lutescens* can be distinguished from other members of *R. alutacea* complex (**Supplementary Figure 3**).

A total of 85 sequences, including 14 newly sequenced ones, were involved in the ITS matrix region of subsect. *Xerampelinae*. These sequences corresponded to nine species and six complexes in this subsection, as well as two species of *R. nitida* (Pers.) Fr. as an out-group. The matrix was 638 bp in length, and there were 138 bp of ITS-1, 167 bp of 5.8S, and 276 bp of ITS-2 in the database. Of the 638 total characters in the ITS matrix of subsect. *Xerampelinae*, 523 were constant, 35 variable characters were parsimony uninformative, and 80 were parsimony informative. The tree had a CI of 0.627, an RI of 0.914, an RC of 0.573, an HI of 0.373, and a TL of 193. The best substitution model selected for Bayesian analysis of subsect. *Xerampelinae* was SYM+I+G. The independence of *R. paraxerampelina* and *R. prunicolor* in the ITS phylogenetic analyses was demonstrated by their strongly supported clades, although the phylogenetic position of *R. paraxerampelina* in subsection *Xerampelinae* is still ambiguous. The clade composed of *R*. *prunicolor* and *R*. *graveolens* received significant support (MLBS). The Chinese samples of *R. subrubens* are closely nested with the European ones (**Supplementary Figure 4**).

### Taxonomy

*Russula alboflava* C.Y. Niu, W.Q. Qin and G.J. Li, sp. nov.

**Figure 2:**
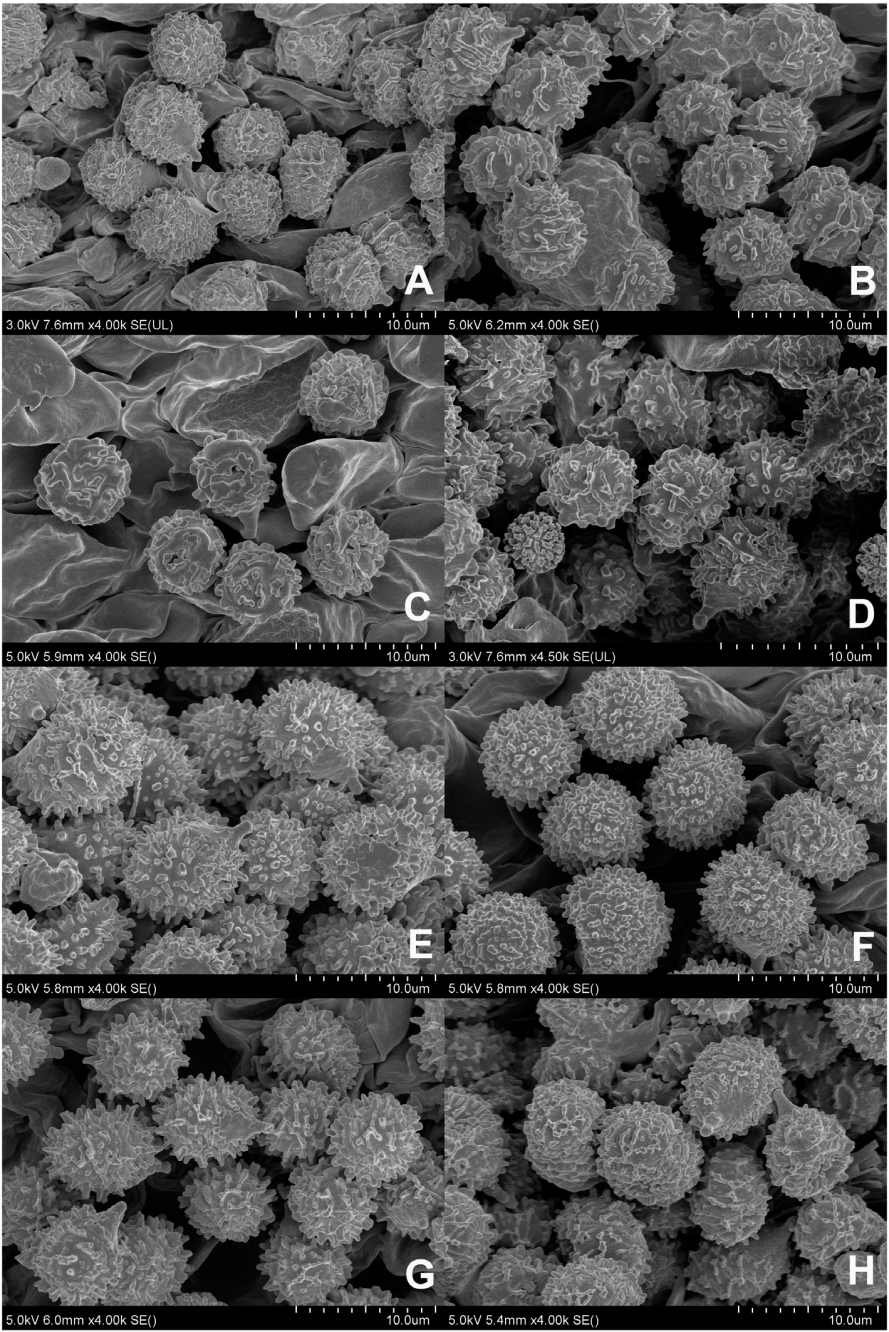
Scanning electron microscope images of basidiospores from *Russula* crown clade species. **(A)** *Russula alboflava* (HBAU15267, holotype), **(B)** *Russula chrysantha* (CFSZ12240, holotype), **(C)** *Russula fulvograminea* (HBAU15886), **(D)** *Russula liyui* (CFSZ12885, holotype), **(E)** *Russula lutescens* (CFSZ19664, holotype); **(F)** *Russula paraxerampelina* (CFSZ21050, holotype), **(G)** *Russula prunicolor* (CFSZ19604, holotype), **(H)** *Russula subrubens* (CFSZ12356).

**Figure 3:**
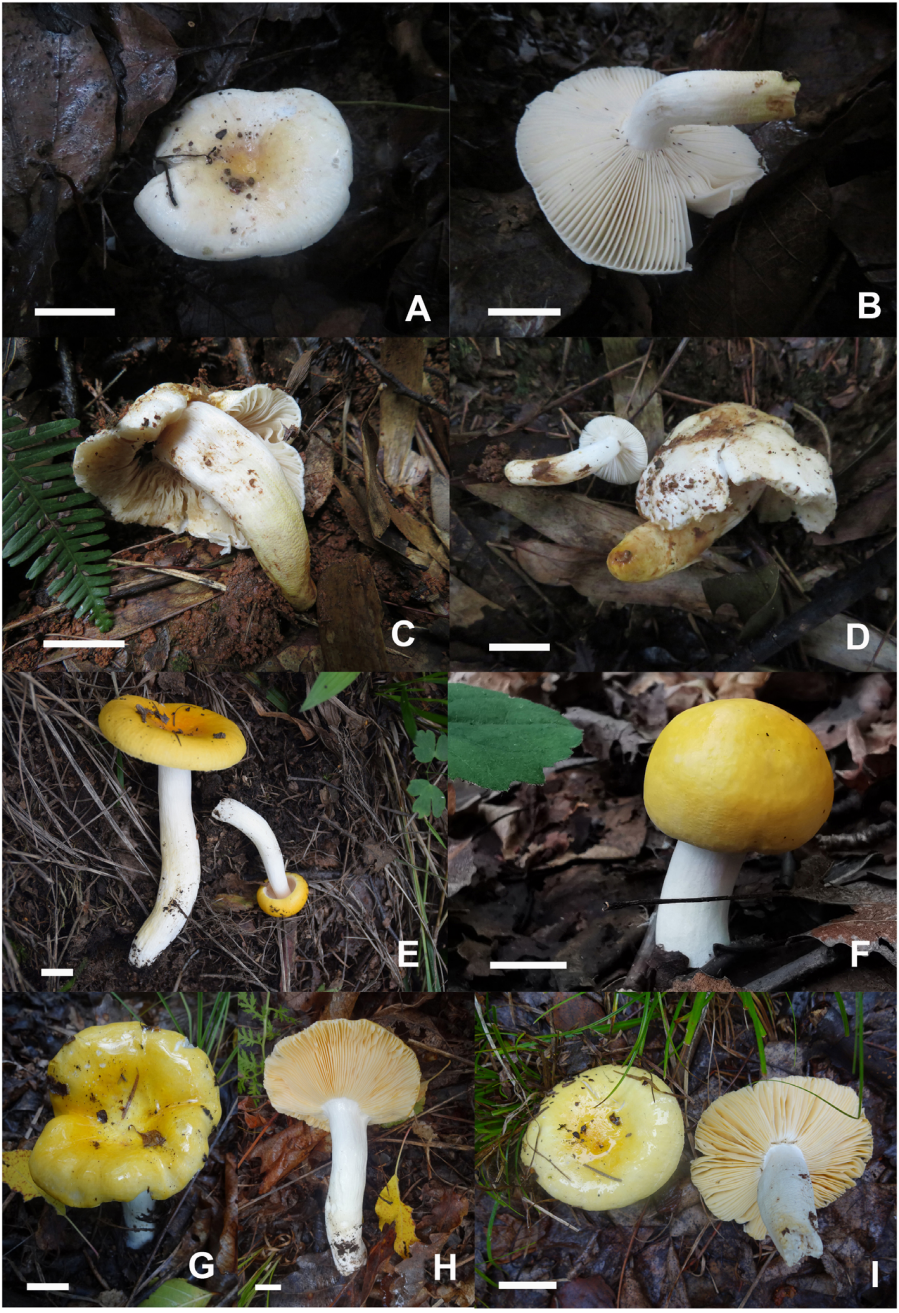
Basidiomata of *Russula alboflava*. **(A, B)** HBAU15267, holotype, **(C, D)** HBAU15294; *Russula chrysantha*, **(E)** HBAU15519, **(F)** CFSZ21012, **(G, H)** CFSZ 12240, holotype, **(I)** CFSZ19597.

**Figure 4:**
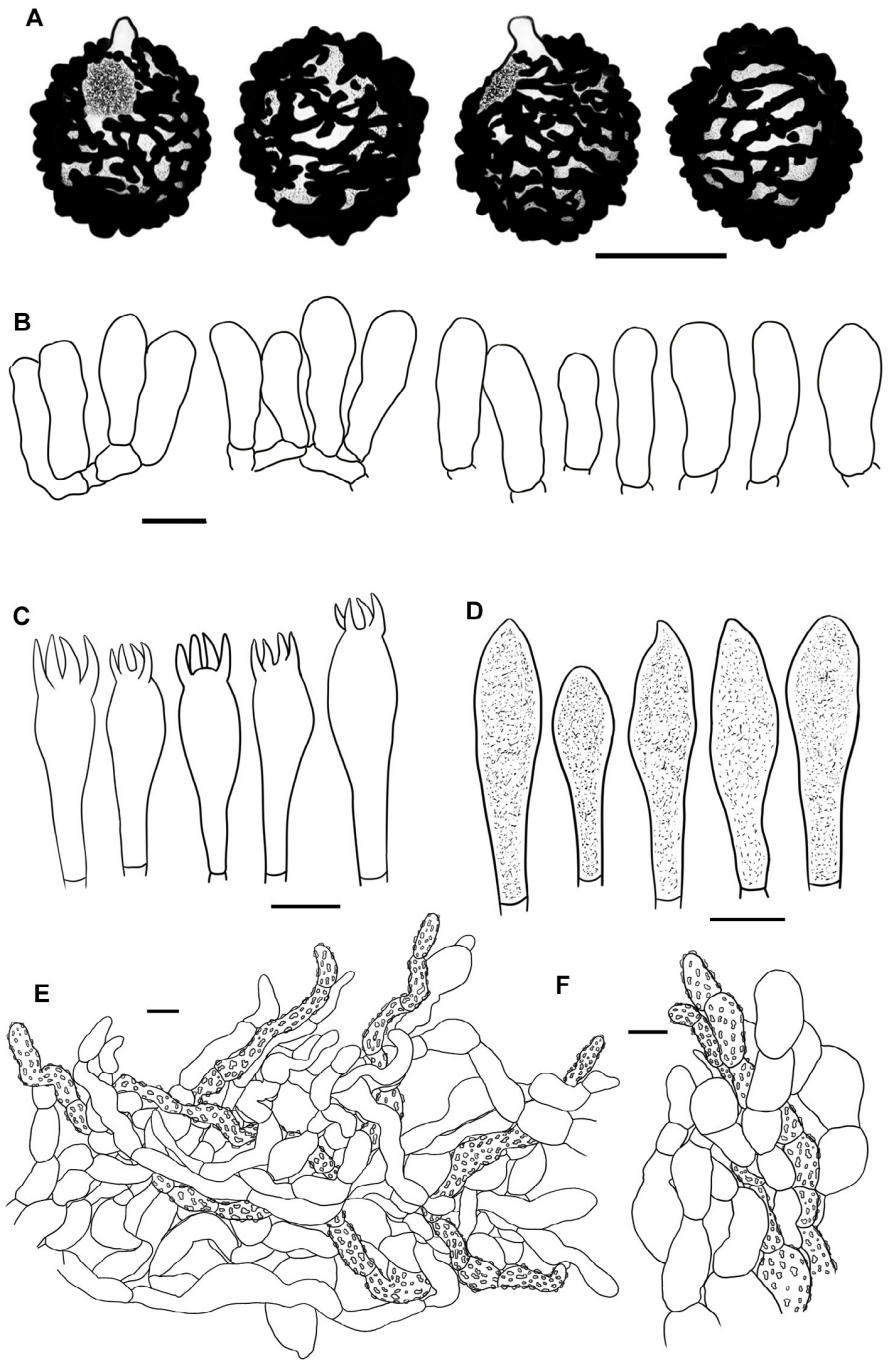
Line drawings of microscope images of *Russula alboflava* (HBAU15267, holotype). **(A)** basidiospores, **(B)** marginal cells, **(C)** basidia, **(D)** pleurocystidia, **(E)** suprapellis in the pileus center, **(F)** suprapellis at the pileus margin.

**Figures 2A**, **3A–D**, **4**.

Fungal Names FN 571960.

Diagnosis: Pileus 23–38 mm in diam., whitish tinged with pale yellowish; margin acute, indistinctly striated. Lamellae adnate to adnexed, cream to pale ochraceous; edge even; lamellulae absent. Stipe 27–43 × 5–12 mm, clavate to subclavate, white in middle-upper part, ochraceous to pale yellow towards the base. Context 1–3 mm thick at pileus center, white, turning ochraceous when bruised, taste mild, odor indistinct. Spore print cream to pale ocher. Basidiospores (5.4–) 5.8–7.6 (–8.1) × (–5.0) 5.3–6.6 (–7.0) μm, ornamentations 0.3–0.8 µm in height, partly to completely reticulate, rarely isolated. Basidia 23–38 × 9–12 μm, clavate to subclavate. Pleurocystidia 27–49 × 6–12 μm, subclavate to clavate, infrequently fusiform; apex obtuse to bluntly acuminate; cheilocystidia not observed; lamellar edges infertile. Pileipellis composed of two layers. Suprapellis is an epithelium at pileus center, and a trichoderm at pileus margin. Primordial hyphae present. Pileocystidia absent. Habitat is forests of broad-leaved Fagaceae.

Etymology: The epithet ‘*alboflava*’ refers to the yellow-tinged white pileus.

Holotype: China, Hunan Province, Zhangjiajie City, Yongding District, Jishou University, Zhangjiajie Campus, hill at the back of campus, 8 July 2019, W.Q. Qin 20190640 (HBAU15267).

Description: Basidiomata small to very small. Pileus 23–38 mm in diam.; first hemispheric, then expanding to convex, and plano-convex in old specimens, applanate, often depressed at center when mature; glossy, glutinous to viscid when young and wet; whitish tinged with pale yellowish, Aniline Yellow (IV19i), Honey Yellow (XXX19′′), to Chamois (XXX19′′b) at center, Light Buff (XV17′f), Cartridge Buff (XXX19′′f), to Marguerite Yellow (XXX23′′f) towards the margin, sometimes completely fading to White (LIII) when mature; margin acute, slightly incurved when young, becoming planate when mature, often cracked, indistinctly striated 1/6–1/5 from the edge inwards, peeling 1/6–1/4 of the radius. Lamellae adnate to adnexed, 2–3 mm at midpoint of disc radius, fragile, frequently forked near the stipe, initially white, becoming cream-colored or pale ochraceous with tinges of Cream Color (XVI19′f), Maize Yellow (III19f) to Martius Yellow (III23f) when mature, turning pale yellowish with tinges of Mustard Yellow (XVI19′b) to Buff Yellow (IV20d) when bruised; edges even, 15–20 blades in 1 cm near the pileus margin, lamellulae absent. Stipe central to faintly decentered, 27–43 × 5–12 mm, clavate to subclavate, sometimes subcylindrical, tapering towards the base, annulus absent, longitudinally rugulose on most of the surface, areolate squamules present near the base, White (LIII) in the middle-to-upper part, ochraceous to pale yellowish with tinges of Light Orange Yellow (III17d), Buff Yellow (IV21d) to Apricot Yellow (IV19b) towards the base, turning Antimony Yellow (XV17′b), Ochraceous Buff (XV15′b) to Warm Buff (XV17′d) when bruised; stuffed when young, becoming tubular to hollow with age. Context 1–3 mm thick at pileus center, fragile, white (LIII) when young, slowly turning ochraceous tinged with Yellow Ocher (XV17′) to Ochraceous-Tawny (XV15′i) when bruised, taste mild, odor indistinct. Spore print cream to pale ocher (IIb–IIIa).

Basidiospores [100/2/2] (5.4–) 5.8–7.6 (–8.1) × (–5.0) 5.3–6.6 (–7.0) μm, Q = (1.02–) 1.05–1.27 (–1.29), (Q = 1.17 ± 0.07), subglobose to broadly ellipsoid, occasionally globose; ornamentations composed of subconical to verrucous, rarely subcylindrical warts, 0.3–0.8 µm in height, dense to very dense (9–14 in a 3 µm diam. circle), partly to completely reticulate, rarely isolated, mostly fused in long, branched lines (3–7 fusions in the circle), frequently connected by fine lines (2–6 in the circle); suprahilar spot large, amyloid, smooth, radically projected at the edge. Basidia 23–38 × 9–12 μm, clavate to subclavate, four-spored, hyaline; sterigmata 5–7 µm in length, somewhat tortuous. Marginal cells 17–25 × 5–10 μm, cylindrical to subcylindrical, occasionally subclavate. Hymenial cystidia pleurocystidia widely dispersed, 50–120/mm^2^, 27–49 × 6–12 μm, subclavate to clavate, infrequently fusiform, projecting 10–20 μm beyond the hymenium; apex obtuse to bluntly acuminate; contents granular, densely distributed, blackish gray in SV; cheilocystidia not observed; lamellar edges infertile. Pileipellis composed of two layers, somewhat difficult to distinguish from spherocytes in the context. Suprapellis an epithelium 50–70 µm thick at pileus center, composed of pseudoparenchymatous, inflated, mostly oblique, occasionally erect, septate, hyaline hyphae, often turning pale yellow in KOH; terminal cells obviously inflated to ellipsoid, 12–35 × 5–15 μm, apex obtuse, infrequently tapered; subapical cells mostly cylindrical, occasionally bifurcated, 6–12 µm in width; primordial hyphae rare, 1–3 celled, 4–7 µm in width, acid-resistant granulate incrustations. Suprapellis in pileus margin a trichoderm 60–100 µm thick, composed of horizontal, oblique to erect hyphae; terminal cells cylindrical to subcylindrical, often inflated to ellipsoid and broadly ellipsoid, 10–100 × 4–15 μm, more or less tapered towards the apex; subapical cells cylindrical, sometimes flexuous; primordial hyphae present, cylindrical, 4–6 µm in width. Subpellis a cutis, 70–120 µm thick, composed of procumbent, cylindrical, septate hyphae 3–8 μm in width, frequently interwoven inflated elements 10–14 μm in width. Pileocystidia absent. Clamp connections not observed in all tissues.

Habit and habitat: Single to scattered in soil in broad-leaved forests dominated by *Castanea henryi*, *Castanopsis eyrei*, *Castanopsis fargesii*, *Castanopsis tibetana*, *Lithocarpus corneus*, *Quercus acutissima*, and *Q. glauca*.

Known distribution: central China (Hunan Province).

Additional specimens examined: China, Hunan Province, Zhangjiajie City, Yongding District, Jishou University, Zhangjiajie Campus, hill at the back of campus, 4 July 2019, W.Q. Qin 20190669 (HBAU15283); ibid, Zhushitou National Forest Farm, 3 August 2019, W.Q. Qin 20190686 (HBAU15294).

Notes: *Russula alboflava* is characterized by a white pileus with a pale yellowish color at the center, an ochraceous scurfy stipe base, and a habitat in subtropical forests dominated by Fagaceae spp. *Russula burlinghamiae* Singer and *R. ballouii* Peck known from the eastern coast of North America, as well as *R. alboflava*, share the morphological characters of cream-white to grayish-yellow pileus surface, strongly scurfy lower stipe surface, mild taste, cream-colored to pale ocher spore print, and broad-leaved trees in habitat (Peck, 1913). *Russula burlinghamiae* differs in having a minutely granular pileus with a separable pellicle on the margin and larger basidiospores, 8.5 × 6.5 µm (Singer, 1938; Buyck et al., 2003).

*Russula ballouii* can be distinguished from *R*. *alboflava* by its pale brick-red pileus edge cracking into minute scales, larger basidiospores, 8–10 µm in diam., and a habitat of poplar forests (Peck, 1913). *Russula alboflava* is also somewhat similar to the subsect. *Chamaeleontinae* Singer species in having a glabrous pileus surface, absence of pileocystidia, mild tasting context, and presence of primordial hyphae in pileipellis (Sarnari, 1998). The members of subsect. *Chamaeleontinae*, such as *R. helios* Malençon ex Sarnari, *R. ochracea* Fr., *R. olivacens* (Fr.) Fr., and *R. risigallina* (Batsch) Sacc., can be distinguished by their yellow spore print and slender pileipellis hyphae (Sarnari, 2005).

*Russula alboflava* is similar to *R. burlinghamiae* Singer and *R. ballouii* Peck from the eastern coast of North America in ITS and multi-gene phylogeny (**Figure 1**; **Supplementary Figure 1**). The ITS analysis indicated that two Japanese samples originally identified as *R. ballouii* (LC667103) and unknown *Russula* species (UDB014137) were closely clustered with *R*. *alboflava*. This indicated that *R*. *alboflava* may have a wider distribution in East Asia. The lineages of *R*. *amethystina*, *R*. *burlinghamiae*, and *R*. *risigallina* form a reasonably well-supported clade (MLBS 63, BSPP 0.93) in the multi-gene analysis (**Figure 1**). The independent phylogenetic position of the *R*. *burlinghamiae* lineage in this clade suggests that it represents an unknown subsection.

*Russula* subsect. *Alboflavinae* G.J. Li, subsect. nov.

Fungal Names FN 571961.

Type species: *Russula alboflava* C.Y. Niu, W.Q. Qin and G.J. Li.

Diagnosis: Pileus initially glabrous, cuticle rarely pulverulent, disrupted into scales when mature, cream-colored tinged with yellow or brick red; lamellae white, cream, to pale ochraceous, lamellulae absent; context fragile, taste mild; stipe base often scurfy to squamulose, yellowish towards base; spore ornamentations composed of warts and ridges linked with fine lines; hymenial cystidia dispersed; pileus two-layered, suprapellis an epithelium with inflated, ellipsoid to versatile pseudoparenchymatous hyphae, pileocystidia rare, primordial hyphae present; habitat broad-leaved forests dominated by Fagaceae.

Etymology: The specific epithet “*alboflavae*” refers to the type species *R. alboflava*.

Species included: *R*. *alboflava* and *R*. *burlinghamiae* from Asia and North America, possibly also *R. ballouii*.

Notes: This new subsection has a close relationship with subsect. *Amethystinae* (Romagn.) Bon and subsect. *Chamaeleontinae* Singer (**Figure 1**). This indicates that the new subsection includes members of subg. *Russula* emend. Buyck & V. Hofst., sect. *Amethystinae* (Romagn.) Sarnari following recent hierarchical classification (Sarnari, 1998; Buyck et al., 2018). Subsection *Alboflavinae* can be distinguished from subsect. *Amethystinae* and subsect. *Chamaeleontinae* as having a scurfy to squamulose stipe base, cream to pale ochraceous spore print and pseudoparenchymatous tissue composed of obviously inflated hyphae in the pileipellis. Of these two closely related taxa, subsect. *Chamaeleontinae* has more similarities with subsect. *Alboflavinae*, such as initially glabrous pileus, inflated pileipellis elements, and a habitat of broad-leaved forests (Sarnari, 2005). The exact phylogenetic position of subsect. *Alboflavinae* in sect. with respect to *Amethystinae* still remains unresolved by the multi-gene phylogenetic analyses of Buyck et al. (2018) and this study.

*Russula chrysantha* C.Y. Niu, T.Z. Liu and G.J. Li, sp. nov.

**Figure 5:**
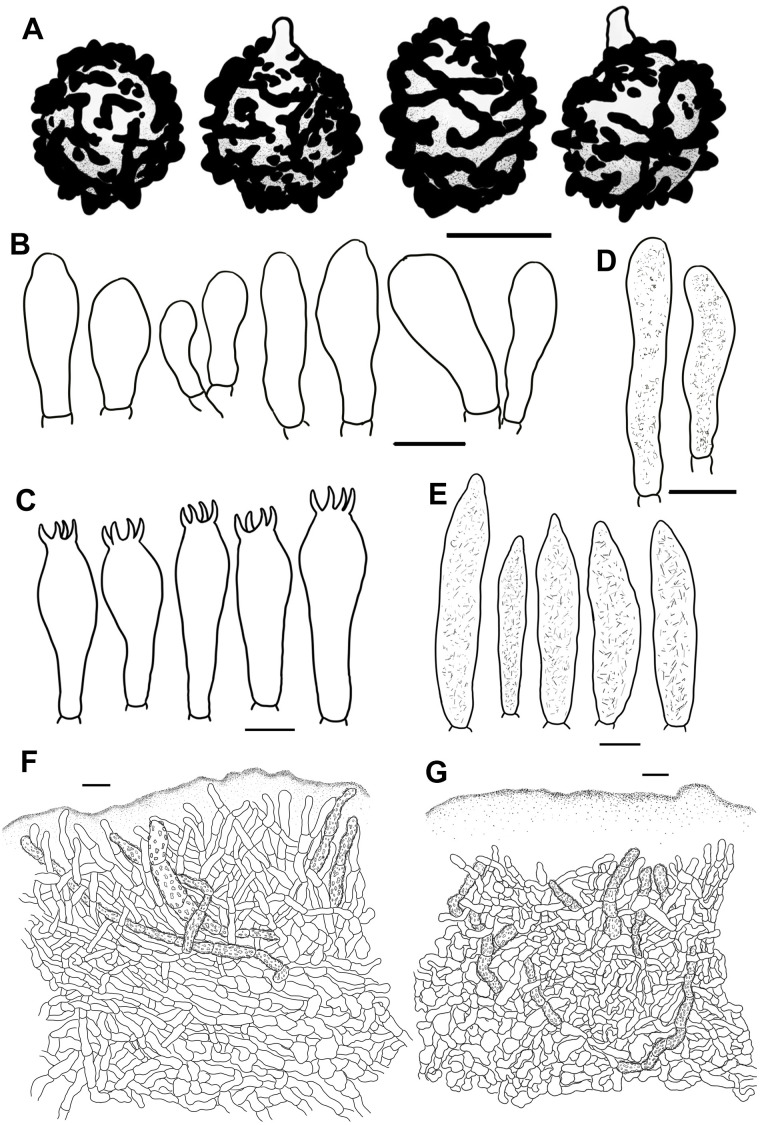
Line drawings of microscope images of *Russula chrysantha* (CFSZ12240, holotype). **(A)** basidiospores, **(B)** marginal cells; **(C)** basidia, **(D)** cheilocystidia, **(E)** pleurocystidia, **(F)** suprapellis in the pileus center, **(G)** suprapellis at the pileus margin.

**Figures 2B**, **3E–I**, **5**.

Fungal Names FN 571962.

Diagnosis: Pileus 34–62 mm in diam., bright yellowish, often fading towards the edge; margin subacute to acute, mostly not striated. Lamellae adnate, sometimes decurrent, ochraceous to pale yellowish; edges even, partly fluctuant; lamellulae rare. Stipe 41–76 × 9–14 mm, cylindrical to subcylindrical, white tinged with pale yellow. Context 2–3 mm thick at pileus center, taste mild with no distinct odor. Spore print deep yellow. Basidiospores (6.0–) 6.4–8.8 (–9.3) × (5.2–) 5.7–7.3 (–7.7) μm, subglobose to broadly ellipsoid, globose or, uncommonly, globose and ellipsoid; ornamentations 0.4–0.9 µm in height, subreticulate, often fused as short crests and ridges. Basidia 27–46 × 10–13 μm, clavate to subclavate. Pleurocystidia 35–58 × 7–13 μm, fusiform, subfusiform to subcylindrical; apex often bluntly acuminate, rarely obtuse; cheilocystidia rare; lamellar edges fertile. Pileipellis two-layered. Suprapellis an ixotrichoderm at pileus center and margin. Primordial hyphae present. Pileocystidia not observed. Habitat in broad-leaved forests of *Quercus*.

Etymology: The specific epithet ‘*chrysantha*’ refers to the bright yellow pileus similar to a chrysanthemum flower.

Holotype: Inner Mongolia Autonomous Region, China, Chifeng City, Bairin Youqi, Saihanwula National Nature Reserve, Wangfengou, 12 September 2016, T.Z. Liu & Z.L. Song (CFSZ12240).

Description: Basidiomata small to medium-sized. Pileus 34–62 mm in diam., subglobose to hemispheric when young, then umbonate, convex to pulvinate, turning planate with age, often centrally concave; glutinous to viscous when wet, smooth, sometimes glabrous, bright yellow, Capucine Yellow (III15b), Capucine Orange (III15d) to Apricot Yellow (IV19b) at the center, often fading into paler tinges of Light Orange Yellow (III17d), Pale Orange Yellow (III17f) to maize yellow (III19f) towards the margin, Naples Yellow (XVI19′d), Massicot Yellow (XVI21′f) to Naphthalene Yellow (XVI23′f) when mature; margin subacute to acute, incurved at first, then flat when mature, infrequently wavy, mostly not striated, rarely indistinctly striated, 1/5–1/3 from the edge inwards, peeling 1/7–1/4 of the radius. Lamellae adnate, rarely adnexed, 2–4 mm in height at the mid-point of pileus radius, occasionally forked near stipe and margin, often interveined, ochraceous to pale yellowish, initially Naples Yellow (XVI19′d) to Straw Yellow (XVI21′d), Mustard Yellow (XVI19′b) to Primuline Yellow (XVI19′) ultimately, unchanging when bruised; edge even, partly fluctuant, 14–22 blades per cm at the pileus margin; lamellulae rare. Stipe central, occasionally subcentral, 41–76 × 9–14 mm, cylindrical to subcylindrical, sometimes subclavate, indistinctly tapered towards the base, annulus absent, initially smooth, longitudinally rugulose when mature, White (LIII), partly stained with pale yellowish tinges of Light Orange Yellow (III17d) to Pale Orange Yellow (III17f) at lower parts, first solid, then hollow with age. Context 2–3 mm thick at pileus center, unchanging when bruised, occasionally becoming ochraceous tinged with Cinnamon Buff (XXIX15′′d) to Chamois (XXX19′′b), brittle, taste mild, no distinct flavor or odor. Spore print deep yellow (IVb–IVd).

Basidiospores [100/2/2] (6.0–) 6.4–8.8 (–9.3) × (5.2–) 5.7–7.3 (–7.7) μm, Q = (1.01–) 1.05–1.32 (–1.41), (Q = 1.19 ± 0.09), subglobose to broadly ellipsoid, globose, uncommonly globose and ellipsoid; ornamentations composed of verrucous, subcylindrical to subconical, infrequently conical amyloid warts, 0.4–0.9 µm in height, subreticulate, moderately distant to dense (4–9 in a 3 µm diam. circle), often fused as short crests and ridges [(1–)2–4 fusions in the circle], dispersedly to frequently connected by lines [(0–)1–3(–4) in the circle]; suprahilar spot amyloid, smooth to slightly verrucose, indistinctly descending. Basidia 27–46 × 10–13 μm, clavate to subclavate, occasionally subcylindrical, four-spored, hyaline; sterigmata 4–6 µm in length, slightly incurved. Marginal cells 27–46 × 5–10 μm, clavate to subclavate, more or less flexuous. Hymenial cystidia pleurocystidia widely dispersed, 150–270/mm^2^, 35–58 × 7–13 μm, fusiform, subfusiform to subcylindrical, occasionally subclavate, projecting 10–20 μm beyond hymenium; apex often bluntly acuminate, rarely obtuse; contents crystalline to granular, sparsely distributed, grayish in SV; cheilocystidia widely dispersed, 33–44 × 10–13 μm, subfusiform to cylindrical; apex obtuse; contents same as those of pleurocystidia; lamella edges fertile. Pileipellis two-layered, composed of suprapellis and subpellis, unambiguously distinguished from the spherocytes below. Suprapellis 60–100 µm thick, an ixotrichoderm at pileus center, composed of gelatinized, mostly vertical to oblique, rarely horizontal, hyaline hyphae, infrequently branched; terminal cells 10–20 × 3–7 µm, cylindrical to clavate, often ventricose, rarely tapered at apex; subapical cells 10–25 × 4–7 µm, cylindrical, infrequently flexuous or branched; primordial hyphae infrequent, 6–8 μm in width. Suprapellis 50–70 µm thick at pileus margin, an ixotrichoderm composed of gelatinized, mainly erect to suberect hyaline hyphae, arising from the underlying inflated subpellis hyphae; terminal cells 6–15 × 3–8 µm, cylindrical to somewhat flexuous; subapical cells cylindrical, at times inflated; primordial hyphae 2–3(–5) celled, cylindrical, 5–7 μm in width. Subpellis a cutis, 70–80 µm thick, composed of mainly horizontal, interlaced, hyaline, subcylindrical, inflated to more or less irregularly shaped hyphae, 4–8 μm in width; cystidioid cells infrequent. Pileocystidia not observed. Clamp connections absent in all tissues.

Habit and habitat: dense to scattered in broad-leaved forests of *Quercus aliena*, *Q. dentata*, *Q. mongolica*, *Q. variabilis*, and *Q. wutaishansea.*

Known distribution: northern China (Hebei Province, Inner Mongolia Autonomous Region).

Additional specimens examined: China, Inner Mongolia Autonomous Region, Chifeng City, Bairin Youqi, Saihanwula National Nature Reserve, Zhenggou, 2 September 2008, T.Z. Liu, H.M. Tian & C. Sun (CFSZ3779); Chifeng City, Harqin Qi, Ma’anshan Forest Park, 3 September 2019, T.Z. Liu & Y.M. Gao (CFSZ21558); ibid, (CFSZ21532); Chifeng City, Harqin Qi, Meilin Township, Taipingzhuang Village, 6 August 2018, T.Z. Liu, Y.Q. Guan, N. Liu (CFSZ19597); Chifeng City, Hexigten Qi, Jingpeng Township, Hongguang Village, 15 August 2017, T.Z. Liu & G.L. Yu (CFSZ18271); Chifeng City, Ningcheng County, Heilihe National Nature Reserve, Sandaohe Village, 17 August 2018, T.Z. Liu & T.T. Yu (CFSZ19655); ibid, Sidaogou Village, 22 August 2019, T.Z. Liu & T. Li (CFSZ21012).

Notes: The new species is a member of subsect. *Chamaeleontinae* because of the glabrous surface of its pileus, mild taste of the context, yellow spore print, inflated terminal cell apex in suprapellis, presence of primordial hyphae in pileipellis, and habitat in broad-leaved forests (Sarnari, 1998, 2005). The new species could be confused with *R. flaviceps* Peck, *R. gilva* Zvára, *R. helios* Malençon ex Sarnari, *R*. *ochracea* Fr., *R*. *postiana* Romell, and *R*. *risigallina*, which all have a yellowish pileus. *Russula flaviceps* can be distinguished from *R*. *chrysantha* as having a slightly acrid context, higher spore ornamentations 0.9–1.1 µm in height, and hymenial cystidia with mostly acute apices and an appendix 3–9 (–11) μm in length (Adamčík et al., 2013). *R. gilva* was described as having a context with a mustard smell, narrower basidia, 9–11 µm in width, and longer hymenial cystidia, 67–85 × 7.5–9 µm. *R. helios* can be distinguished by its larger pileus, 70–100 mm in diam., wider basidia, 45–58 × 11.5–16 µm, and narrow primordial hyphae, 3–4 µm in width. *R*. *ochracea* is differentiated from *R*. *chrysantha* by the presence of a pinkish tinge along the pileus margin, spore ornamentations composed of mostly isolated warts, and narrower suprapellis hyphae up to 5 µm in width (Romagnesi, 1985; Sarnari, 2005). *R*. *postiana* differs in sometimes having a pale grayish green pileus, larger basidiospores, 8.5–9.5(–10) × 7–8.5 µm, and narrow apical cells, 2–3 µm, in the pileipellis (Ruotsalainen and Vauras, 1990). *R*. *risigallina* was described as having a mostly red to orange pileus, old basidiomata with an odor of withered roses, spore ornamentations composed of more or less isolated warts and spines, and narrower hymenial cystidia, 7–10 µm in width (Romagnesi, 1985; Sarnari, 2005).

For those members of the subsect. *Chamaeleontinae*, originally described from Asia, *R. miyunensis* C.L. Hou, H. Zhou, & G.Q. Cheng can be distinguished from *R*. *chrysantha* by the presence of a dark red to brown pileus, hymenial cystidia with an appendix 2–5 μm in length, and a habitat of *Carpinus turczaninowii* forest. *R. plana* C.L. Hou, H. Zhou, & G.Q. Cheng was described as having a brick-red to deep red pileus, shorter and wider basidia, (22–)23.4–33.2(–38) × (10–)12.1–15.9(–18) µm, as well as mostly one-celled primordial hyphae (Zhou et al., 2022a). *R*. *brunneopurpurea* Jabeen & Khalid differs in having a purple to brownish-purple pileus, white to cream spore print, basidiospore ornamentations composed of isolated warts, and mucronate to rostrate hymenial cystidia (Jabeen et al., 2017).

*Russula chrysantha* is the sister species of *R. flaviceps* Peck from the eastern United States, *R. helios*, *R. postiana* and *R. risigallina* (synonym *R. vitellina* Gray) described from Europe, and *R. brunneopurpurea* reported from South Asia. These species cannot be clearly distinguished through ITS phylogenetic analyses (**Supplementary Figure 1**). This new species can be differentiated from *R. olivascens*, *R. risigallina*, and North American specimens identified as *R. lutea* (Huds.). See the gray zone in the multi-gene phylogram (**Figure 1**).

*Russula fulvograminea* Ruots., Sarnari and Vauras, Riv. Micol. 40(2): 99, 1997.

**Figure 6:**
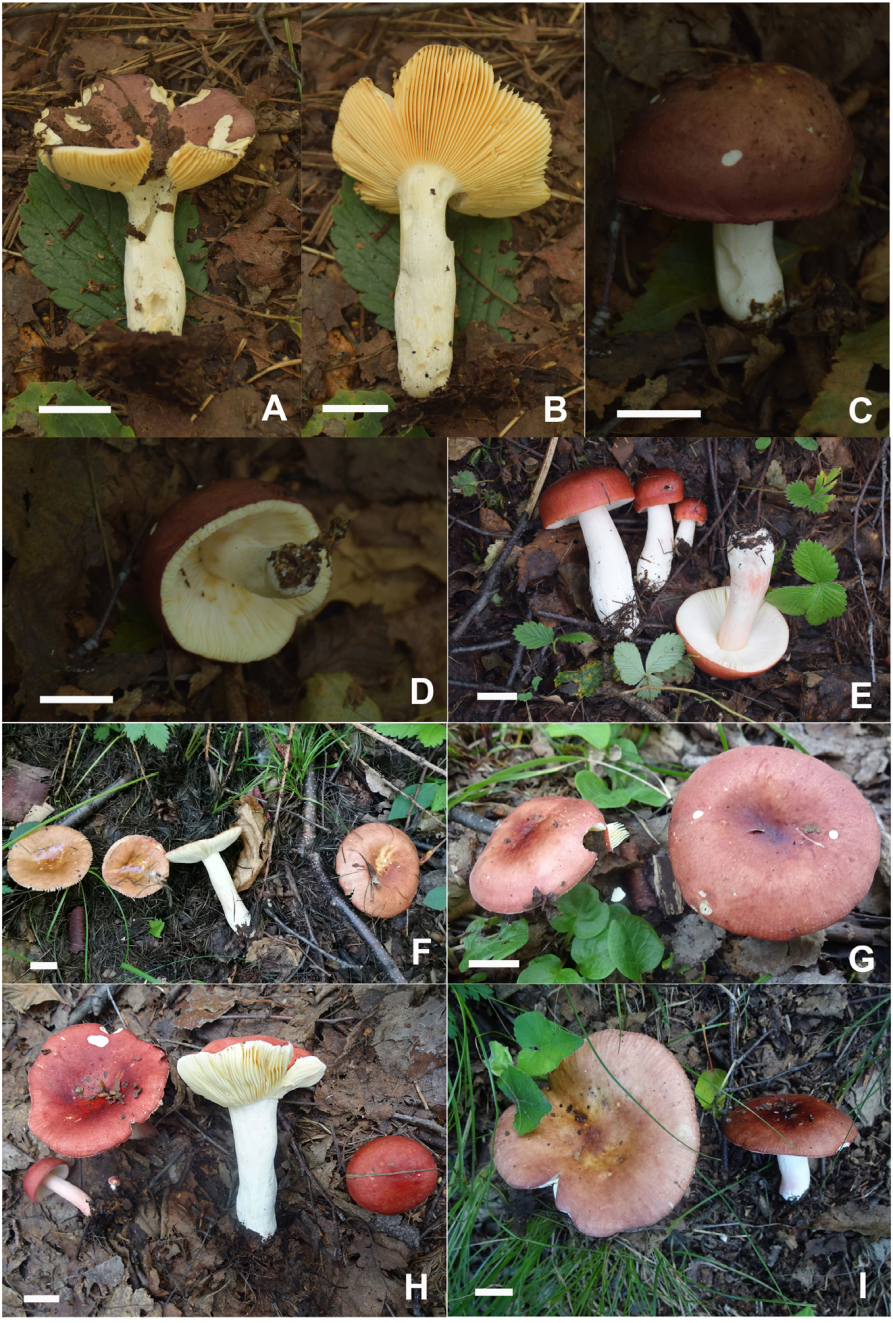
Basidiomata of *Russula fulvograminea*. **(A, B)** HBAU15885, **(C, D)** HBAU15886; *Russula liyui*, **(E)** HBAU15541, **(F)** CFSZ12885, **(G)** CFSZ19470, **(H)** CFSZ19480, **(I)** CFSZ19497.

**Figure 7:**
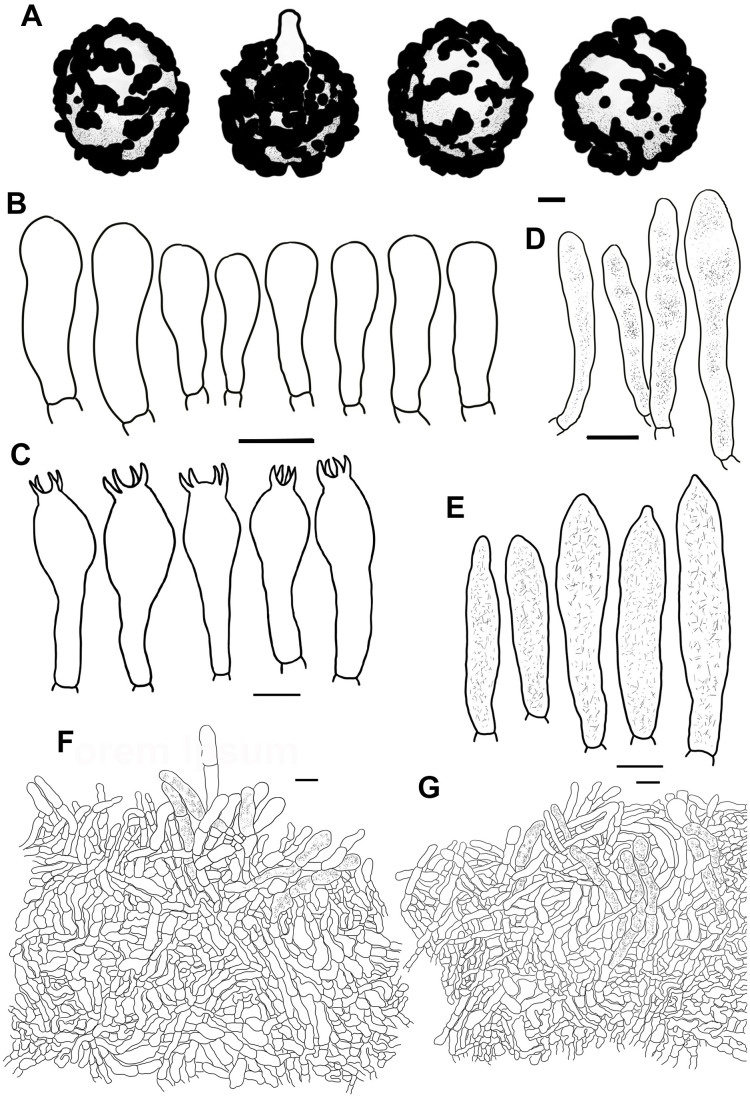
Line drawings of microscope images of *Russula fulvograminea* (HBAU15886). **(A)** basidiospores, **(B)** marginal cells; **(C)** basidia, **(D)** cheilocystidia, **(E)** pleurocystidia, **(F)** suprapellis in the pileus center, **(G)** suprapellis at the pileus margin.

**Figures 2C**, **6A–D**, **7**.

MycoBank MB 442901.

Description: Basidiomata mostly small- to medium-sized, rarely very small. Pileus 28–55 mm in diam., initially plano-hemispheric to umbonate, then becoming convex to flat-convex, flat to acetabuliform when mature, often shallow infundibuliform; slightly viscid when wet, dull, often partly exfoliated; erythrinus, pale to gray tinged with purple, Light Purple Drab (XLV1′′′′b), Light Vinaceous Drab (XLV5′′′′b), to Light Brownish Drab (XLV9′′′′b), sometimes Fawn Color (XL13′′′), army brown (XL13′′′i) to Deep Vinaceous Purple (XLIV69′′′), centrically faded to Light Vinaceous Gray (XXXIX9′′′d), Pale Vinaceous Brown (XXXIX3′′′f) to Pale Vinaceous Gray (XXXIX5′′′f); margin acute, faintly introverted at first, then expanding in a planate manner to fluctuant, often curled-up, frequently cracked, indistinctly striated 1/7–1/6 from the edge inwards, peeling 1/5–1/3 towards the center. Lamellae adnate to slightly decurrent, 2–4 mm in height at mid-point of pileus radius, rarely forked near the stipe and edge, interveined, ochraceous to pale yellowish, originally Cream Buff (XXX19′′d) to Pale Orange Yellow (III17f), turning Antimony Yellow (XV17′b) to Light Ochraceous-Buff (XV15′d) when mature, unchanging or turning Pale Ochraceous with tinges of Ochraceous Buff (XV15′b) to Yellow Ocher (XV17′) when bruised; edge even, 12–18 pieces at 1 cm from the pileus margin, lamellulae present. Stipe central to sub-central, 30–62 × 8–15 mm, cylindrical, sometimes subcylindrical, annulus absent, longitudinally rugulose, surface white, often ochraceous tinged with Cinnamon Buff (XXIX15′′d) to Clay Color (XXIX17′′) when bruised, farctate when young, hollow with age. Context 2–3 mm thick at pileus center, white (LIII), unchanging, slowly becoming cream to pale ochraceous with tinges of Cream Buff (XXX19′′d) to Chamois (XXX19′′b) when bruised, taste mild, no distinct odor. Spore print dark yellow (IVc–IVd).

Basidiospores [100/2/2] (6.0–) 6.5–8.6 (–9.9) × (5.1–) 5.5–7.7 (–8.3) μm, Q = (1.01–) 1.03–1.27 (–1.32), (Q = 1.15 ± 0.08), globose, subglobose to broadly ellipsoid; ornamentations composed of moderately distant to dense [(4–)5–7(–8), a 3 μm diam. circle] amyloid verrucose warts and spines, 0.3–0.7 µm in height, partly subreticulated, occasionally to frequently fused in clusters and short chains [1–3(–4) fusions in the circle], connected by dispersed to occasional fine lines [(0–)1–2 in the circle]; suprahilar spot large, covered with low amyloid ornamentation. Basidia 25–47 × 10–14 μm, often clavate to subclavate, rarely cylindrical to subcylindrical, four-spored, hyaline; sterigmata 4–7 μm, incurved, rarely straight. Marginal cells 15–26 × 5–8 μm, clavate to subclavate, infrequently subcylindrical. Hymenial cystidia pleurocystidia widely dispersed to dispersed, 300–650/mm^2^, 32–58 × 8–11 μm, cylindrical, subcylindrical to subclavate, projecting 10–25 μm beyond the hymenium; apex obtuse to bluntly acuminate, occasionally papilliform; contents crystalline to granular, heterogeneous, grayish in SV; cheilocystidia dispersed, 35–65 × 7–12 μm, clavate, subclavate to subfusiform; apex obtuse; lamellar edges infertile. Pileipellis stratified, composed of suprapellis and subpellis, sharply delimited from spherocytes in context tissue. Suprapellis 100–120 μm thick in pileus center, a trichoderm composed of vertical to subvertical, occasionally diverticulate, hyaline hyphae; terminal cells 10–19 × 3–7 μm, cylindrical to subclavate, infrequently constricted towards the apex; subapical cells 7–13 × 3–6 μm, cylindrical, rarely branched. Suprapellis 100–120 µm thick at pileus margin, possessing a trichoderm composed of interweaved vertical, subvertical to horizontal, rarely diverticulate, hyaline hyphae; terminal cells 7–15 × 3–7 μm, cylindrical to subcylindrical, infrequently ventricose or tapered at apex; subapical cells cylindrical, rarely flexuous. Subpellis a cutis, 50–70 µm thick, composed of mostly repand, subcylindrical to irregularly shaped, loosely interlaced, hyaline hyphae; cystidioid cells not observed. Pileocystidia abundant in pileus margin, mostly 1–5 septate, rarely multi-septate, 5–8 μm in width; apex obtuse; contents unevenly distributed, crystalline to granular, grayish in SV; pileocystidia fewer in pileus center, morphology same as those in pileus margin. Clamp connections not observed in all tissues.

Habit and habitat: solitary or scattered in intermixed broadleaved and coniferous forests dominated by *Betula costata*, *B*. *platyphylla*, *Larix gmelinii* var. *principis-rupprechtii*, *Picea meyeri*, *Picea wilsonii*, and *Pinus tabuliformis*.

Known distribution: Finland (Sarnari, 2005), northern China (Hebei Province, Inner Mongolia Autonomous Region), and Russia (Vladimir Oblast, https://wikigrib.ru).

Specimens examined: China, Inner Mongolia Autonomous Region, Chifeng City, Ningcheng County, Heilihe National Nature Reserve, 26 August 2021, S.Y. Zhao, C.Y. Niu, S. Chen, X.L. Gao & G.J. Li, 20210986 (HBAU15885). Hebei Province: Chengde City, Luanping County, Baicaowa National Forest Park, 21 August 2022, C.Y. Niu & G.J. Li 20220338 (HBAU15886); ibid, Shijiazhuang City, Lingshou County, Nanying Township, Caofang Village, and Wuyuezhai National Forest Park, G.J. Li, Y.B. Guo, X.J. Xie, X. Zhang, T.T. Fan 20200124 (HBAU15500).

Notes: *Russula fulvograminea* was originally described from Europe based on key characters of a pileus tinged with purple, brown, or green, pale yellow spore print, slightly diverticulate pileipellis hyphae, as well as a forest habitat of intermixed broadleaved and coniferous trees, mainly *Betula* and *Picea* species (Sarnari, 2005). Most of the morphological characters are in correspondence with those of Sarnari (2005). This species has not been reported in Asia, thus it is proposed as a new Chinese record. There are minor morphological differences between Asian and European specimens. The latter has a pileus tinged with yellowish to grayish green at the center, wider basidia 29–52 × 10–16 μm, and pileocystidia with 0–2 septa (Sarnari, 2005).

Specimens of *R*. *fulvograminea* formed a highly supported clade in the ITS phylogram (**Supplementary Figure 2**) (MLBS 100, MPBS 100, BSPP 1). The *R*. *fulvograminea* clade clustered with *R*. *laricinoaffinis* Bon and two sequestrate species *R. galileensis* (M.M. Moser, Binyam. & Aviz.-Hersh.) Trappe & T.F. Elliott and *R. vidalii* Trappe & T.F. Elliott (MLBS 58, BSPP 0.97) but this close relationship was not supported in the multi-gene analyses. *Russula fulvograminea* has been identified as a member of subsect. *Integriforminae* (Bon) Sarnari, sect. *Polychromae* (Maire) Sarnari based on morphology in Sarnari (2005). Multi-gene phylogenetic analyses showed that *R*. *fulvograminea* had a close relationship with members of subsect. *Laricinae* (Romagn.) Bon, sect. *Tenellae* Quél. (**Figure 1**).

*Russula liyui* C.Y. Niu, T.Z. Liu and G.J. Li, sp. nov.

**Figure 8:**
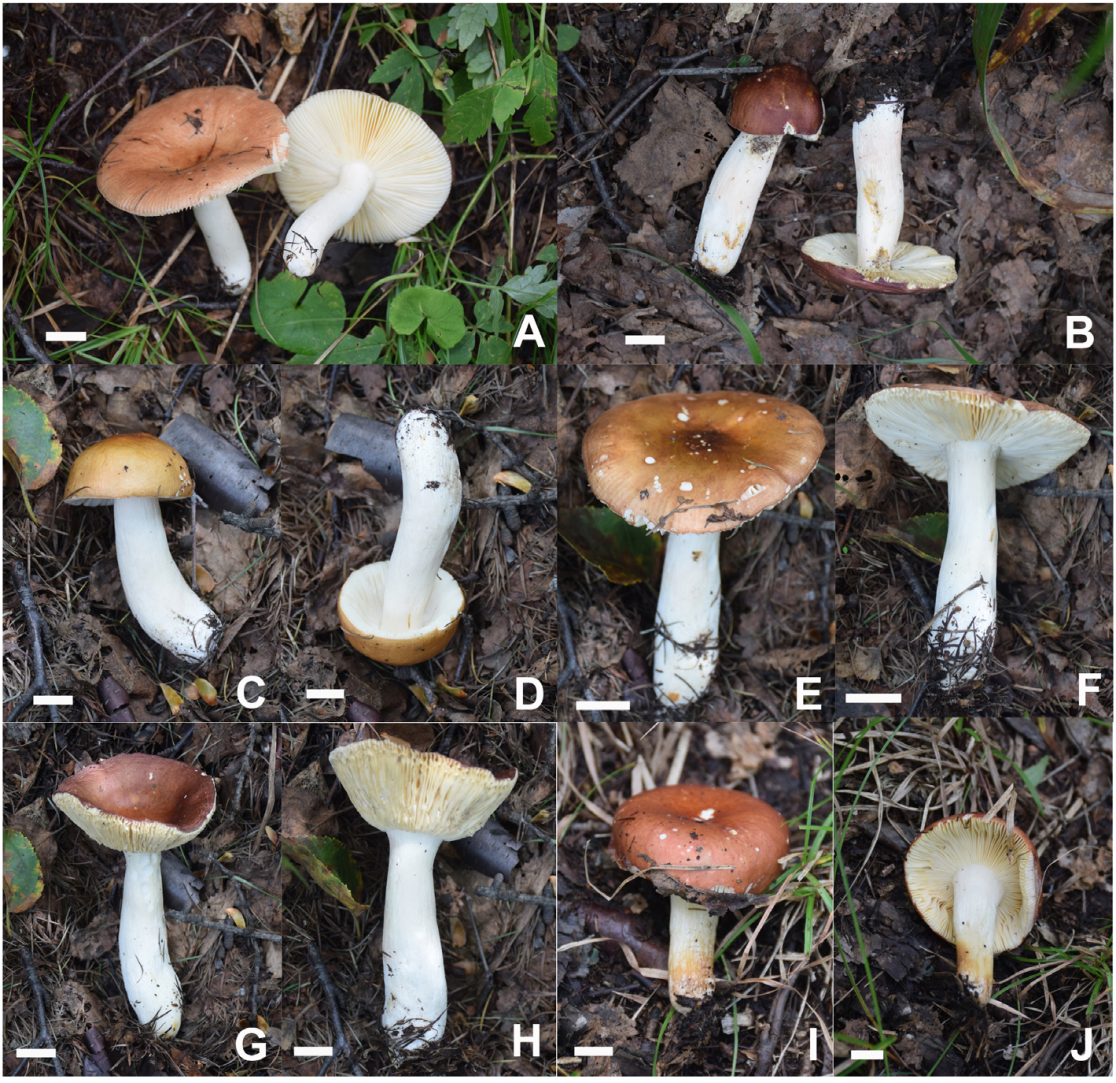
Basidiomata of *Russula liyui*. **(A)** HBAU15541, **(B)** HBAU15384, **(C, D)** HBAU15385, **(E, F)** HBAU 15386, **(G, H)** HBAU15391, **(I, J)** HBAU15395.

**Figure 9:**
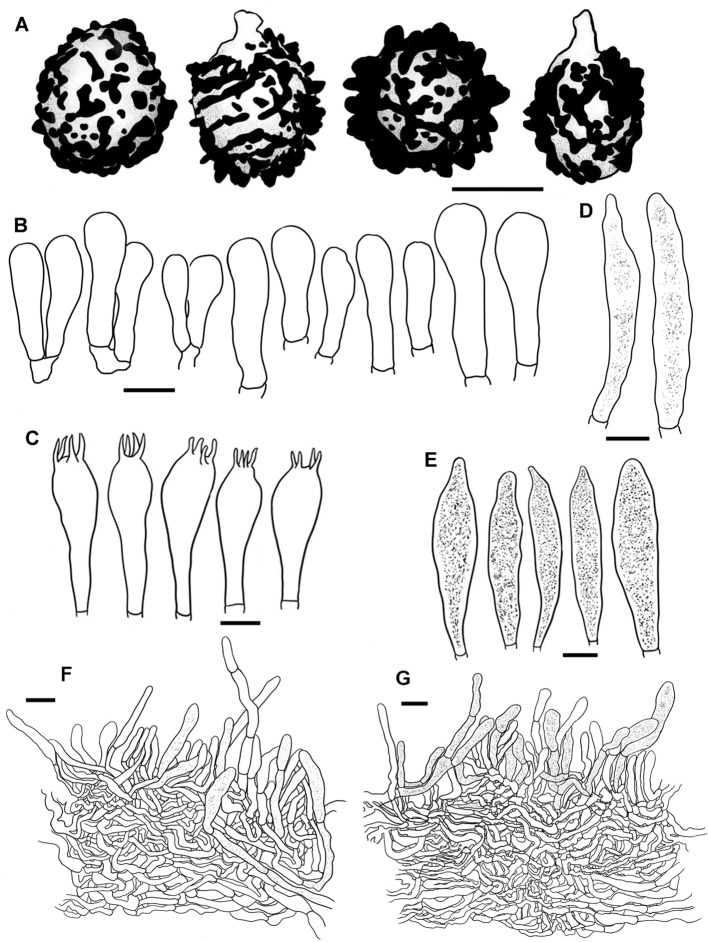
Line drawings of microscope images of *Russula liyui* (CFSZ12885, holotype). **(A)** basidiospores, **(B)** marginal cells; **(C)** basidia, **(D)** cheilocystidia, **(E)** pleurocystidia, **(F)** suprapellis in the pileus center, **(G)** suprapellis at the pileus margin.

**Figures 2D**, **6E–I**, **8**, **9**.

Fungal Names FN 571963.

Diagnosis: Pileus 33–67 mm in diam., variously colored, brownish red to erythrinus, centrally faded to pale ochraceous olive; margin acute, indistinctly striate. Lamellae adnate to adnexed, white to cream-colored; edge even; lamellulae rare. Stipe central, 37–68 × 9–15 mm, cylindrical, subcylindircal to subclavate, surface white. Context 2–4 mm thick at pileus center, white to pale cream; taste mild, no distinct odor. Spore print dark ocher to pale yellow. Basidiospores (5.4–) 5.9–9.6 (–10.2) × (5.3–) 5.8–7.6 (–8.1) μm, subglobose, broadly ellipsoid to ellipsoid, infrequently globose; ornamentations 0.5–1.0 μm in height, dispersed to occasionally linked by fine lines as branched crests and ridges; suprahilar spot large, amyloid. Basidia 28–48 × 9–14 μm, clavate to subclavate. Pleurocystidia fusiform to subfusiform, sometimes subclavate, apex obtuse to bluntly acuminate, infrequently lanceolate; cheilocystidia dispersed; lamellar edges fertile. Pileipellis composed of well-divided suprapellis and subpellis. Suprapellis, a trichoderm in pileus center and on margin. Pileocystidia often fasciculate at the pileus margin, 0–2 septate, 4–9 µm in width, cylindrical. Found in broad-leaved forests of *Betula* spp.

Etymology: The specific epithet “*liyui*” is proposed in honor of Prof. Yu Li, former president of the Jilin Agricultural University, for his outstanding contribution to mycology and edible mushroom cultivation.

Holotype: Inner Mongolia Autonomous Region, Chifeng City, Ningcheng County, Heilihe National Nature Reserve, Dabagou, China, 23 July 2017, T.Z. Liu (CFSZ12885).

Description: Basidiomata small to medium-sized. Pileus 33–67 mm in diam., first hemispheric, then gradually turning convex to planate, at times becoming concave, saucer-shaped to infundibuliform when mature; slightly viscid when wet, smooth, mostly glabrous, rarely dull, occasionally exfoliated in small patches; variously colored, mostly brownish red to erythrinus, Russet-Vinaceous (XXXIX9′′′), Deep Brownish Vinaceous (XXXIX5′′′) to Livid Brown (XXXIX1′′′), sometimes tinged with bright Dragon’s Blood Red (XIII5′i), Coral Red (XIII5′) to Jasper Red (XIII3′), pale pinkish tinges of light Congo Pink (XXVIII7′′d) to Vinaceous Pink (XXVIII9′′d), with darker tinges of deep purplish vinaceous (XLIV69′′′), the center faded to pale ochraceous olive with tinges of Olive Ocher (XXX21′′) to Old Gold (XVI19′); margin acute, initially incurved, undulate to planate in maturity, occasionally curled-up and cracked, indistinctly striate 6/1–3/1 from the edge inwards, peeling 1/5–1/3 towards the center. Lamellae adnate to adnexed, 2–5 mm in height at the midpoint of the disc radius, rarely forked near the stipe, sometimes interveined, primarily white (LIII), becoming pale cream with tinges of Naphthalene Yellow (XVI23′f), Massicot Yellow (XVI21′f) to Cream Color (XVI19′f) with age, unchanging when bruised; edge even, 13–19 blades per cm along the pileus margin, lamellulae rare. Stipe central, 37–68 × 9–15 mm, cylindrical, subcylindircal to subclavate, indistinctly tapered towards the base, annulus absent, smooth at first, but turning longitudinally rugulose with age, surface White (LIII), sometimes becoming ochraceous to pale brownish with tinges of Raw Sienna (III17i) to Aniline Yellow (IV19i) at the lower part, turning Yellow Ocher (XV17′) to Ochraceous-Tawny (XV15′i) when bruised; originally stuffed, becoming fistulous to hollow at last. Context 2–4 mm thick at pileus center, White (LIII), unchanging when bruised, slowly turning pale cream tinged with Cartridge Buff (XXX19′′f) to Light Buff (XV17′f) with age, fragile, taste mild, no distinct odor. Spore print dark ocher to pale yellow (IIId–IVb).

Basidiospores [100/2/2] (5.4–) 5.9–9.6 (–10.2) × (5.3–) 5.8–7.6 (–8.1) μm, Q = (1.02–) 1.07–1.33 (–1.37), (Q = 1.20 ± 0.08), subglobose, broadly ellipsoid to ellipsoid, infrequently globose; ornamentations composed of moderately distant to dense [(3–)4–7 in a 3 μm diam. circle] verrucose to subconical amyloid warts, partly subreticulated, 0.5–1.0 μm in height, occasionally to frequently fused in pairs, triplets or short lines [2–4(–5) fusions in the circle], dispersed to occasionally linked in fine lines as branched crests and ridges [(0–)1–3(–4) in the circle]; suprahilar spot large, amyloid, smooth to slightly uneven, often merged with adjacent ornamentations. Basidia 28–48 × 9–14 μm, clavate to subclavate, four-spored, hyaline; sterigmata 4–7 μm, straight to more or less tortuous. Marginal cells 20–34 × 9–14 μm, clavate to subclavate, occasionally cylindrical. Hymenial cystidia pleurocystidia moderately numerous to numerous 1100–2500/mm^2^, 41–72 × 8–15 μm, fusiform to subfusiform, sometimes subclavate, projecting 15–35 μm beyond hymenium; apex obtuse to bluntly acuminate, infrequently lanceolate; contents granular, dense, evenly distributed, dark gray in SV; cheilocystidia dispersed, 50–70 × 8–13 μm, fusiform to subfusiform; apex bluntly acuminate, rarely lanceolate; lamellar edges fertile. Pileipellis composed of suprapellis and subpellis well divided in 40–65 μm deep. Suprapellis a trichoderm in the center of the pileus composed of upright to more or less oblique, sometimes diverticulate, hyaline hyphae; primordial hyphae absent; terminal cells 13–27 × 3–5 µm, cylindrical, subcylindrical to subclavate; apex obtuse, sometimes inflated; subapical cells 11–30 × 2–5 µm, cylindrical, frequently flexuous. Suprapellis of the pileus margin a trichoderm composed of vertical to repand, rarely diverticulate elements; terminal cells 18–32 × 3–5 µm, cylindrical to subclavate, sometimes flexuous; apex obtuse; subapical cells 14–26 × 3–4 µm, cylindrical, rarely ramified. Subpellis a cutis, 60–90 µm thick, composed of horizontal to slightly ascending, interwoven, cylindrical to subcylindrical, occasionally flexuous hyphae 3–8 µm in width; cystidioid cells not observed. Pileocystidia often fasciculate at the pileus margin, 0–2 septate, 4–9 µm in width, cylindrical, infrequently embedded in the subpellis; apex obtuse; contents granular, dense in the pileus margin, gray in SV; pileocystidia in pileus center 3–8 µm in width, cylindrical, rarely subfusiform, contents relatively sparsely distributed. Clamp connections absent.

Habit and habitat: dense to scattered in broad-leaved forests of *Betula albosinensis* Burkill and *B. platyphylla* Sukaczev. Known distribution: northern China, in Hebei Province and Inner Mongolia Autonomous Region.

Additional specimens examined: China, Inner Mongolia Autonomous Region, Chifeng City, Ningcheng County, Heilihe National Nature Reserve, Sandaohe, 7 July 2010, T.Z. Liu (CFSZ4275); ibid, Sidaogou, 18 July 2004, T.Z. Liu (CFSZ2169); Chifeng City, Harqin Qi, Meilin Township, Taipingzhuang Village, 29 July 2018, T.Z. Liu & Y.Q. Guan (CFSZ19497); ibid, (CFSZ19480); ibid, (CFSZ19470). Hebei Province, Shijiazhuang City, Lingshou County, Nanying Township, Caofang Village, Wuyuezhai National Forest Park, 3 August 2020, G.J. Li, Y.B. Guo, X.M. Jiao, L. Sun 20190849 (HBAU15403); ibid, 20190850 (HBAU15385); ibid, 20190858 (HBAU15391); ibid, 20190861 (HBAU15393); ibid, 20190862 (HBAU15394); ibid, 20190863 (HBAU15395); ibid, 20190848 (HBAU15384); 21 August 2020, G.J. Li, Y.B. Guo, X.J. Xie, X. Zhang, T.T. Fan 20190875 (HBAU15403); ibid, 20200158 (HBAU15520); Shijiazhuang City, Pingshan City, Hehekou Township, Tuoliang National Nature Reserve, 22 August 2020, 20200190 (HBAU15541); ibid, 20200192 (HBAU15542); Chengde City, Pingquan City, Liuxi Township, Dawopu Village, Liaoheyuan National Forest Park, N 27 August 2021, S. Chen, X.L. Gao & G.J. Li, 20211137 (HBAU15880); ibid, 20211126 (HBAU15882); Zhangjiakou City, Chicheng County, Dushikou Township, Dushikou Village, Bingshanliang Scenic Area, 17 August 2021, S.Y. Zhao, C.Y. Niu, S. Chen, X.L. Gao & G.J. Li, 20210443 (HBAU15881).

Notes: Multi-gene phylogenetic analyses indicated that *R. liyui* is a member of sect. *Tenellae*. A combination of mild tasting context, indistinctly striate pileus margin, short basidia, absence of primordial hyphae, and multi-septate pileocystidia also supported this phylogenetic assignment of *R. liyui*. The new species is sister to *R. font-queri* (**Figure 1**). These two closely related species are barely distinguishable in ITS phylogeny (**Supplementary Figure 2**), which could lead to a mistaken identity for the new species as *R. font-queri* in preliminary research (Cao et al., 2019). Both of these species have a bright red to copper red, partly yellow, glabrous pileus, a stipe often flushed with red, a context turning ochraceous to yellow when injured, and a habitat of birch forest. *Russula font-queri* can be distinguished from *R. liyui* as having narrower hymenial cystidia/pileocystidia 9–12/6–9 µm in width, and irregularly formed pileocystidial appendages (Romagnesi, 1985; Sarnari, 2005).

The new species is a member of subsect. *Rhodellinae* (Romagn.) Bon, sect. *Tenellae* following the infrageneric classification of Sarnari (1998). Multi-gene phylogenetic analyses indicated that *R. font-queri* and *R. liyui* belong to subsect. *Laricinae*. Several members of this taxon have been reported based on Asian samplings in recent years. Morphological and habitat differences are as follows: *R. laricina* Velen. has a context turning grayish, spore ornamentations composed of mostly isolated warts, and a habitat of coniferous forests (Sarnari, 2005), *R. sichuanensis* G.J. Li & H.A. Wen has agaricoid to secotioid basidiomata, sinuate, contorted, very crowded, convoluted lamellae, and larger basidiospores 9.4–14.1 × 7.9–12.8 µm (Li et al., 2013), *R. vinosobrunneola* G.J. Li & R.L. Zhao has a vinaceous brown coloration on the pileus surface, narrower hymenial cystidia 6–7 μm in width, and slender pileocystidia 3–5 μm in width (Li et al., 2018a).

*Russula lutescens* C.Y. Niu, T.Z. Liu and G.J. Li, sp. nov.

**Figure 10:**
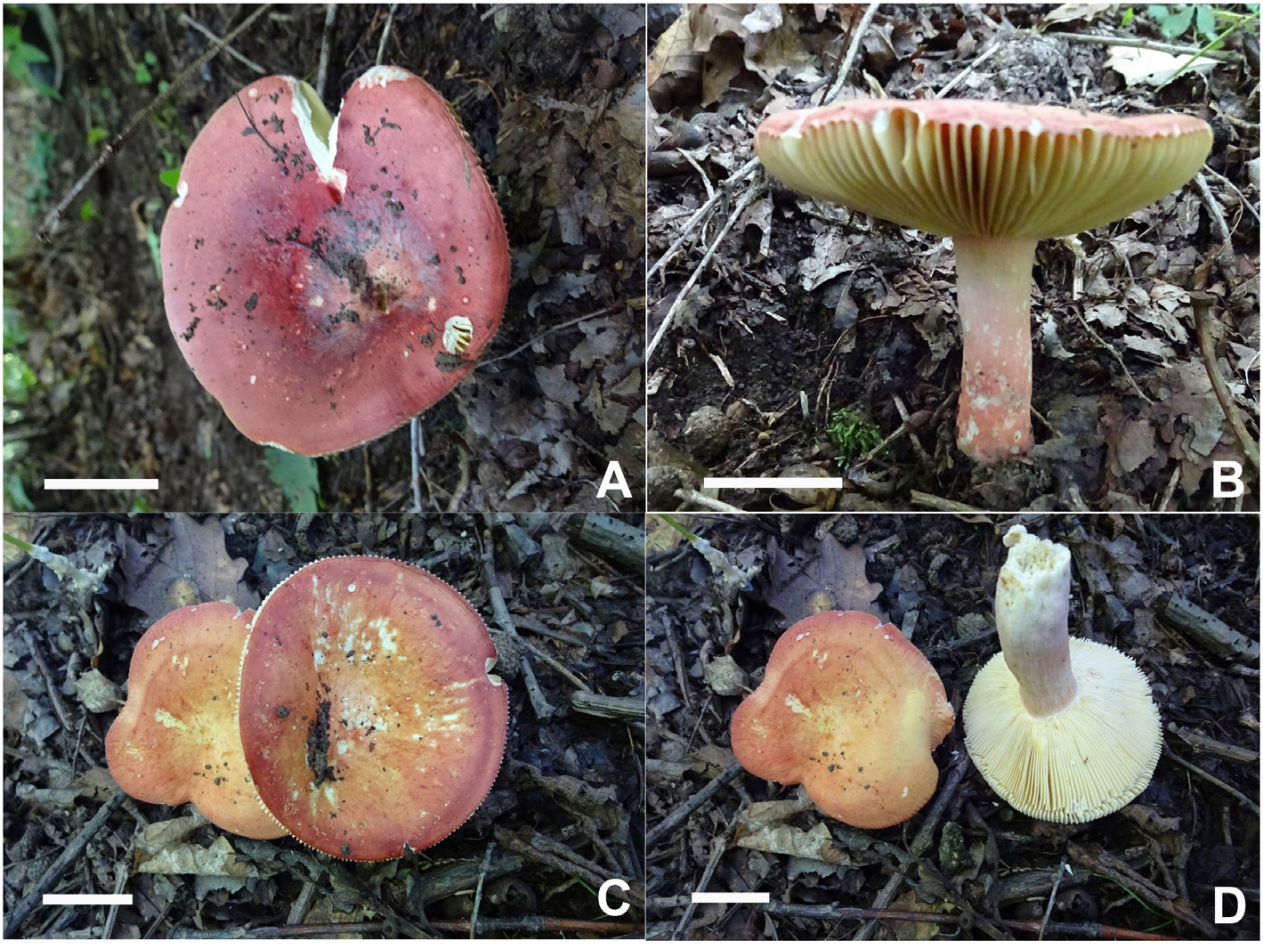
Basidiomata of *Russula lutescens*. **(A, B)** CFSZ19634, **(C, D)** CFSZ19664, holotype.

**Figure 11:**
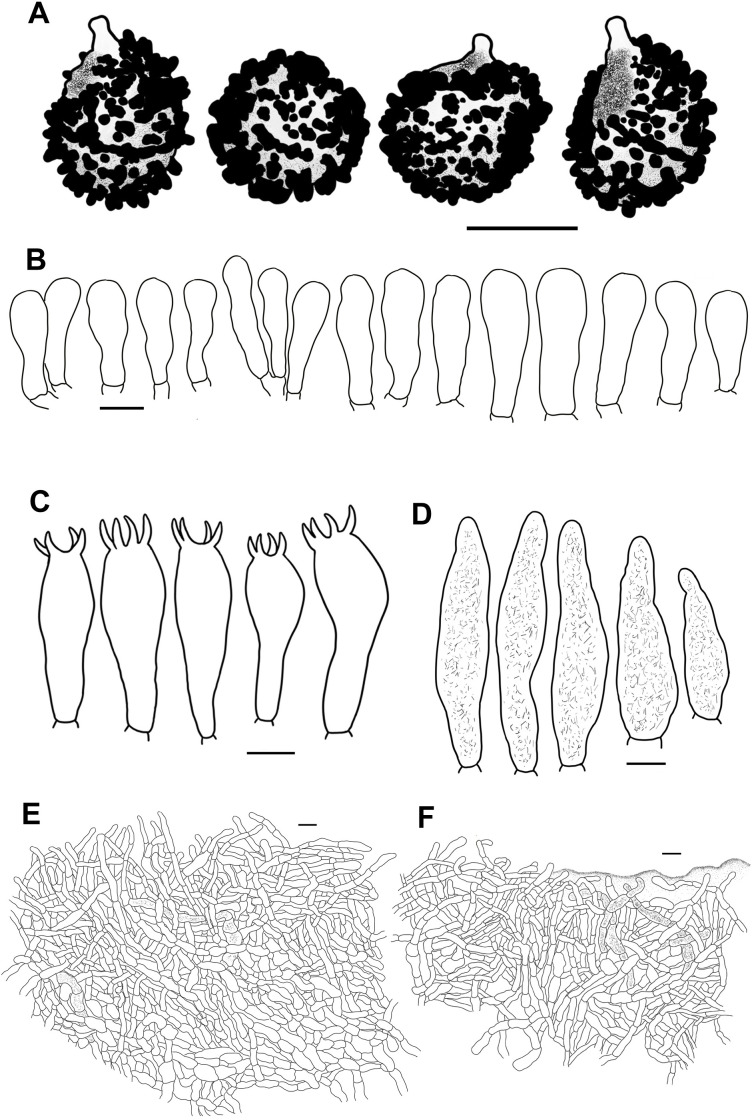
Line drawings of microscope images of *Russula lutescens* (CFSZ19664, holotype). **(A)** basidiospores, **(B)** marginal cells, **(C)** basidia, **(D)** pleurocystidia, **(E)** suprapellis in the pileus center, **(F)** suprapellis at the pileus margin.

**Figures 2E**, **10**, **11**.

Fungal Names FN 571964.

Diagnosis: Pileus 34–63 mm in diam., red to brownish red intermixed with tinges of yellowish ocher; margin subacute to acute, faintly striated. Lamellae adnexed, sometimes adnate, dark cream, ochraceous to pale yellow; edge even, occasionally undulant; lamellulae not observed. Stipe 37–55 × 7–13 mm, cylindrical to subcylindrical, surface mostly pinkish to pale reddish. Context 3–4 mm thick at pileus center, white, taste mild, no distinct odor. Spore print pale yellow. Basidiospores (7.0–) 7.3–10.1 (–10.6) × 6.6–8.6 (–8.9) μm, globose, subglobose to broadly ellipsoid, ornamentations 0.4–1.1 μm in height, dispersed to frequently fused as pairs, triplets, and rarely branched lines; suprahilar spot amyloid. Basidia 34–47 × 10–15 μm, subclavate to subcylindrical. Pleurocystidia 56–84 × 10–15 μm, subclavate, fusiform to subfusiform, infrequently clavate; apex obtuse, occasionally bluntly acuminate. Pileipellis contains two layers of suprapellis and subpellis. Suprapellis is a trichoderm in the center of the pileus, partly an ixotrichoderm. Pileocystidia rare, 3–4 septate, cylindrical, 4–9 µm in width. Habitat in broad-leaved forests of *Quercus*.

Etymology: The specific epithet “*lutescens*” refers to the lutescent pileus.

Holotype: Chifeng City, Ningcheng County, Heilihe National Nature Reserve, Sandaohe Village, 17 August 2018, T.Z. Liu & T.T. Yu (CFSZ19664).

Description: Basidiomata small to medium in size. Pileus 34–63 mm in diam., initially plano-hemispheric to convex, flat to shallow acetabuliform when mature; slightly viscid when wet, dull, smooth, exfoliated in small patches; red to brownish red tinged with yellowish ocher, Hydrangea Red (XXVII1′′i), Mineral Red (XXVII1′′k) to Ocher Red (XXVII5′′b), often becoming a lighter shade of Purplish Vinaceous (XXXIX1′′′b), Russet-Vinaceous (XXXIX9′′′) to Light Russet-Vinaceous (XXXIX9′′′b) towards the margin, faded at the center to Cadmium Yellow (III17), Raw Sienna (III17i) to buff-yellow (IV21d); margin subacute to acute, more or less incurved first, planate at last, rarely cracked at the edge, faintly striated 1/8–1/5 from the edge inwards, peeling 1/6–1/5 towards the center. Lamellae adnexed, sometimes adnate, 2–4 mm in height at the midpoint of the radius, rarely forked near the stipe attachments, often interveined, initially dark cream to ochraceous, becoming pale yellowish with age, Cream Color (XVI19′f), Pale Ochraceous-Buff (XV15′f) to Warm Buff (XV17′d); edge even, occasionally undulant, 10–17 pieces at 1 cm near the pileus margin, lamellulae not observed. Stipe central, occasionally subcentral, 37–55 × 7–13 mm, cylindrical to subcylindrical, indistinctly tapered towards the base, longitudinally rugulose; surface mostly pinkish to pale reddish tinged with Pinkish Vinaceous (XXVII5′′d), Corinthian Pink (XXVII3′′d) to Pale Vinaceous (XXVII1′′), frequently faded to Hydrangea Pink (XXVII5′′f), Livid Pink (XXVII3′′f) to Shell Pink (XXVII11′′f) towards the upper parts, first stuffed, but cavernous to hollow when mature. Context 3–4 mm thick at pileus center, initially White (LIII), unchanging when bruised, gradually turning cream with tinges of Maize Yellow (III19f) to Light Buff (XV17′f) with age, taste mild, no distinct odor. Spore print pale yellow (IVa–IVb).

Basidiospores [100/2/2] (7.0–) 7.3–10.1 (–10.6) × 6.6–8.6 (–8.9) μm, Q = (1.00–) 1.03–1.29 (–1.35), (Q = 1.17 ± 0.08), globose, subglobose to broadly or occasionally ellipsoid; ornamentations composed of moderately distant to dense [(4–)5–7(–8) in a 3 μm diam. circle], cylindrical, subcylindrical to verrucose, rarely subconical amyloid warts, 0.4–1.1 μm in height, dispersed to frequently fused as pairs, triplets, and rarely branched lines (1–4 fusions in the circle), dispersedly to occasionally connected by fine lines [0–1(–2) in the circle]; suprahilar spot amyloid, slightly verrucose, radically merged with nearby ornamentations at edge. Basidia 34–47 × 10–15 μm, subclavate to subcylindrical, four-spored, hyaline; sterigmata 5–8 μm, often incurved. Marginal cells 21–35 × 5–11 μm, clavate to subclavate, rarely subcylindrical to cylindrical, often flexuous. Hymenial cystidia pleurocystidia widely dispersed to dispersed, 170–630/mm^2^, 56–84 × 10–15 μm, subclavate, fusiform to subfusiform, infrequently clavate, projecting 20–55 μm beyond the hymenium; apex obtuse, occasionally bluntly acuminate; contents crystalline, sparse, grayish in SV; cheilocystidia not observed; lamella edges fertile. Pileipellis consists of two layers, the suprapellis and the subpellis, indistinctly distinguished from the underlying spherocytes in the context. The Suprapellis is a trichoderm in the center of the pileus, composed of tightly interlaced, 80–100 μm thick, erect to ascending, infrequently repand, hyaline hyphae; primordial hyphae absent; terminal cells 11–32 × 3–5 μm, cylindrical, occasionally flexuous, apex obtuse, ventricose, rarely tapered; subapical cells 15–25 × 3–5 μm, cylindrical, infrequently branched. Suprapellis of the pileus margin partly an ixotrichoderm, composed of somewhat gelatinized, loosely interwoven, vertical to subvertical elements; terminal cells 14–28 × 3–6 μm, cylindrical to subclavate; apex obtuse; subapical cells 10–15 × 3–5 μm, cylindrical. Subpellis a cutis, composed of repand to slightly oblique, cylindrical, sometimes inflated to ampuliform hyaline hyphal cells 3–9 µm in width; cystidioid cells infrequent. Pileocystidia in the pileus margin rare, 3–4 septate, arising from the subpellis, cylindrical, 4–9 µm in width; contents granular, sparsely and unevenly distributed, gray in SV; pileocystidia in the pileus center dispersedly distributed, 3–5 septate, 4–11 µm in width. Clamp connections absent in all tissues.

Habit and habitat: dense to scattered in broad-leaved forests of *Quercus aliena*, *Q. dentata*, *Q. mongolica*, *Q. variabilis*, and *Q. wutaishansea.*

Known distribution: northern China (Inner Mongolia Autonomous Region).

Additional specimens examined: China, Chifeng City, Ningcheng County, Heilihe National Nature Reserve, Sandaohe Village, 17 August 2018, T.Z. Liu & T.T. Yu (CFSZ19634); ibid, 23 August 2018, T.Z. Liu & Y.H. Tan (CFSZ20035).

Notes: This new species is a member of sect. *Amethystinae*, subsect. *Olivaceinae* Singer with a pale-yellow spore print, non-incrusted primordial hyphae in the pileipellis, and a habitat of broad-leaved forest, following the infrageneric classification of Sarnari (1998). This assignment was also supported by long insertions in the ITS region (Miller and Buyck, 2002). A limited number of species have been identified as members of subsect. *Olivaceinae*, namely *R. alutacea* (Pers.) Fr., *R. olivacea* (Schaeff.) Fr., and *R. vinosobrunnea* (Bres.) Romagn. *Russula alutacea* can be differentiated from *R*. *lutescens* by its reticulated spore ornamentations up to 0.8 μm in height, and longer hymenial cystidia up to 120 μm in length with an acute apex. *R. olivacea* differs in having larger basidiomata, up to 170 mm in diam., a velutinous, occasionally green pileus surface, longer basidia up to 65 μm in length, and long, fusiform hymenial cystidia 72–100 × 8.5–13(–16) μm with lanceolate appendages, and a suprapellis composed of short, bulky, ampullaceous cells 10–15 μm in width; *R. vinosobrunnea* can be distinguished from *R*. *lutescens* as having a larger pileus up to 120 mm in diam., spore ornamentations up to 1.2 μm in height, and longer hymenial cystidia, 60–130 × 8–15 μm (Romagnesi, 1985; Sarnari, 2005).

The *R. lutescens* clade has a close relationship with the samples that were identified as *R. alutacea*, *R. olivacea*, and *R. vinosobrunnea* in ITS phylogeny. These species formed a lineage that was regarded as an *R. alutacea* complex (**Figure 1**). Although ITS analyses revealed a highly specific diversity of subsect. *Olivaceinae*, the concrete multi-gene phylogenetic position of *R. lutescens* remains unknown because of limitations in sampling and sequencing (**Supplementary Figure 3**).

*Russula paraxerampelina* C.Y. Niu, T.Z. Liu and G.J. Li, sp. nov.

**Figure 12:**
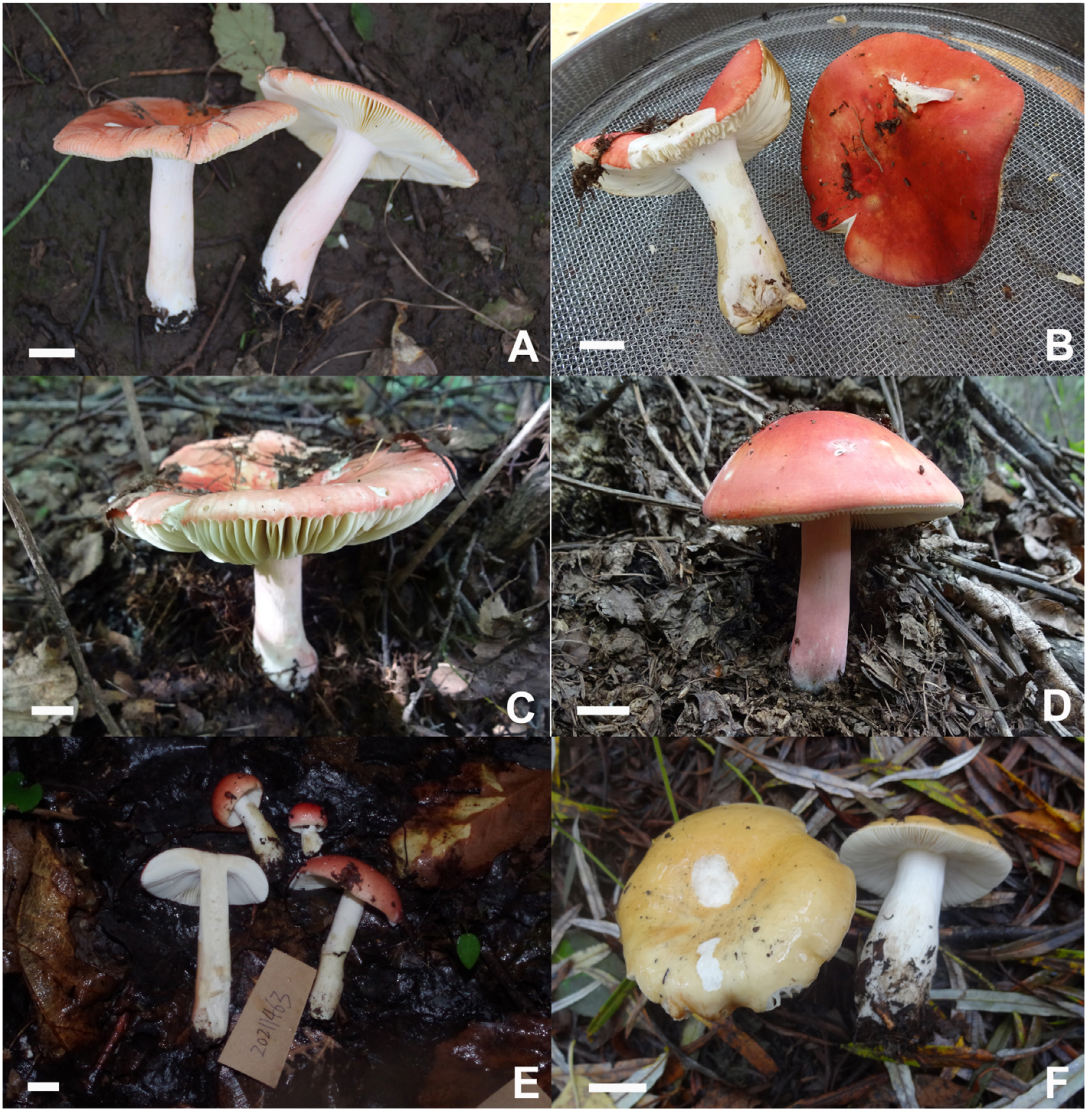
Basidiomata of *Russula paraxerampelina*. **(A)** HBAU15572, **(B)** CFSZ21050, holotype **(C)** CFSZ 21512, **(D)** CFSZ21181; *Russula prunicolor* **(E)** HBAU15883; *Russula subrubens* **(F)** CFSZ12952.

**Figure 13:**
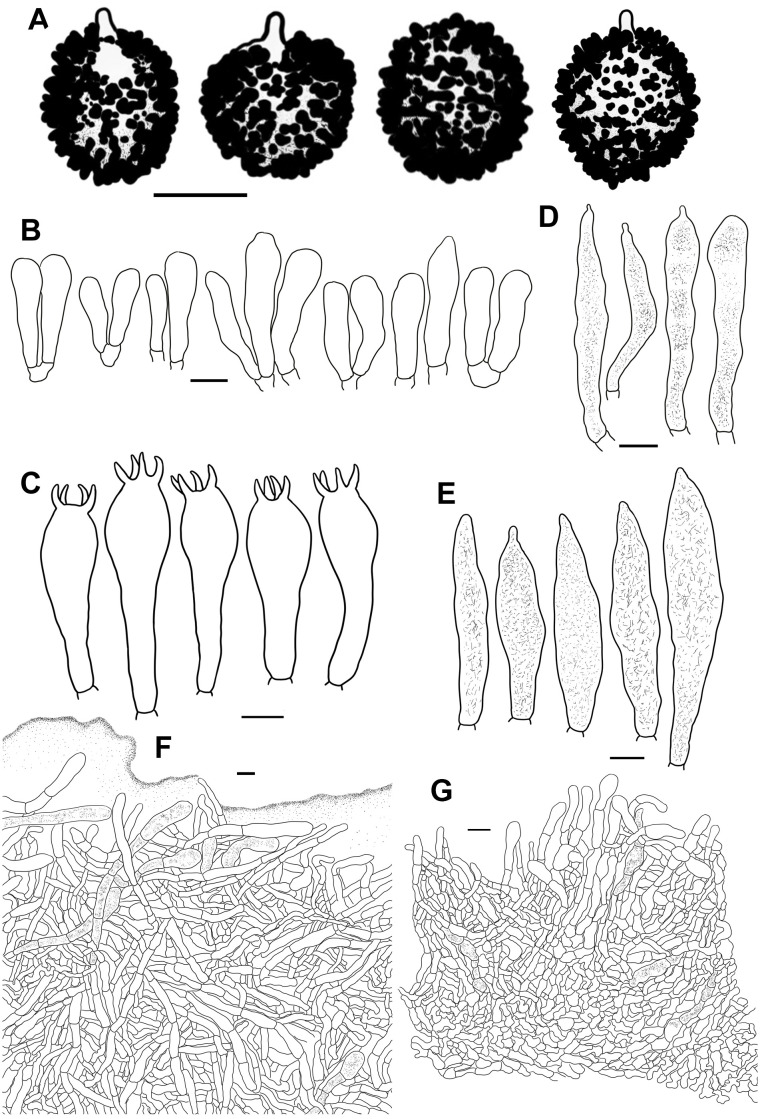
Line drawings of microscope images of *Russula paraxerampelina* (CFSZ21050). **(A)** basidiospores, **(B)** marginal cells; **(C)** basidia, **(D)** cheilocystidia, **(E)** pleurocystidia, **(F)** suprapellis in the pileus center, **(G)** suprapellis at the pileus margin.

**Figures 2F**, **12A–D**, **13**.

Fungal Names FN 571965.

Diagnosis: Pileus 43–78 mm in diam., bright reddish to pinkish, margin subacute to acute, indistinctly striated. Lamellae adnate to adnexed, dark cream tinged with pale ochraceous in older specimens; edge even; lamellulae rare. Stipe 40–84 × 9–15 mm, clavate, subclavate to subcylindrical, surface often white, pink to red. Context 3–5 mm thick at pileus center, white, cream to pale ochraceous; taste mild, odor more or less smelly. Spore print dark ocher to pale yellow. Basidiospores (7.2–) 7.5–10.0 (–11.3) × (5.6–) 6.0–9.0 (–9.7) μm, ornamentations 0.3–1.0 μm in height, frequently fused in pairs and chains; suprahilar spot large, amyloid. Basidia 32–62 × 11–16 μm, clavate to subclavate. Pleurocystidia 52–87 × 8–16 μm, fusiform to subfusiform, rarely subclavate and subcylindrical; apex bluntly acuminate, shortly lanceolate to papilliform, occasionally obtuse; cheilocystidia present. Pileipellis two-layered with suprapellis and subpellis. Suprapellis an ixotrichoderm at the center of the pileus and a trichoderm at the margin. Pileocystidia in the pileus margin dispersive in the suprapellis and subpellis, mostly 3–5 celled, cylindrical, infrequently fusiform, 5–10(–13) µm in width; Habitat in mixed coniferous and broad-leaved forests of *Larix*, *Picea*, *Pinus*, and *Quercus*.

Etymology: The specific epithet ‘*paraxerampelina*’ is named for its similarity to *R. xerampelina* (Schaeff.) Fr.

Holotype: China, Inner Mongolia Autonomous Region, Chifeng City, Ningcheng County, Heilihe National Nature Reserve, Sidaogou, 23 August 2019, T.Z. Liu & L.G. Yin (CFSZ21050).

Description: Basidiomata small- to medium-sized. Pileus 43–78 mm in diam., initially hemispheric to convex, becoming plano-convex to flat when mature, often slightly depressed in the center; slightly viscid when wet, smooth, more or less glabrous; bright reddish to pinkish colored, carmine (I1i), Brazil Red (I5i) to English Red (II7i), occasionally Coral Pink (XIII5′d), Orient Pink (II9f) to Safrano Pink (II7f), intermixed with some tinges of Bittersweet Orange (II9b), Flame Scarlet (II9) to Chrome Orange (II11), Ochraceous Buff (XV15′b) to Yellow Ocher (XV17′) when bruised; margin subacute to acute, initially incurved, becoming outstretched with age, undulate and curled up at times, infrequently cracked, indistinctly striated 1/8–1/6 from the edge inwards, peeling 1/4–1/3 of the radius. Lamellae adnate to adnexed, 2–4 mm in height at mid-point of the radius, often forked near the edge, interveined, initially white (LIII), dark cream, pale ochraceous in age, Light Buff (XV17′f), Pale Ochraceous Buff (XV15′f) to Cream (XVI19′f); edge even, 13–21 blades per cm along the pileus margin; lamellulae rare. Stipe central, rarely subcentral, 40–84 × 9–15 mm, clavate, subclavate to subcylindrical, somewhat ventricose towards the lower parts, annulus absent, initially smooth, longitudinally rugulose when mature, surface often White (LIII), partly to completely flushed with reddish and pinkish tinges of Coral Pink (XIII5′d), Pale Vinaceous (XXVII1′′) to Flesh Pink (XIII5′f), farctate when young, becoming fistulous to hollow with age. Context 3–5 mm at pileus center, initially White (LIII), unchanging when bruised, gradually becoming cream to pale ochraceous with tinges of Pale Ochraceous Buff (XV15′f) to light Ochraceous Buff (XV15′d) with age; taste mild, odor more or less smelly when old. Spore print dark ocher to pale yellow (IIId–IVa).

Basidiospores [100/2/2] (7.2–) 7.5–10.0 (–11.3) × (5.6–) 6.0–9.0 (–9.7) μm, Q = (1.02–) 1.07–1.33 (–1.39), (Q = 1.19 ± 0.08), subglobose, broadly ellipsoid to ellipsoid, infrequently globose, ornamentations composed of dense to very dense [(8–)9–12(–14) in a 3 μm diam. circle] verrucose to subcylindrical amyloid warts, 0.3–1.0 μm in height, frequently fused in pairs and chains [(2–)3–4(–5) fusions in the circle], dispersedly to occasionally linked with fine lines [0–2 in the circle]; suprahilar spot large, amyloid, subtly warty, irregularly projected in edge. Basidia 32–62 × 11–16 μm, clavate to subclavate, occasionally subcylindrical, four-spored, hyaline; sterigmata 3–7 μm, mostly tortuous. Marginal cells 18–38 × 5–11 μm, clavate to subclavate, sometimes subcylindrical or subfusiform. Hymenial cystidia pleurocystidia dispersed to moderately numerous (300–1100/mm^2^) 52–87 × 8–16 μm, fusiform to subfusiform, rarely subclavate or subcylindrical, projecting 15–45 μm beyond hymenium; apex bluntly acuminate, shortly lanceolate to papilliform, occasionally obtuse; contents crystalline to granular, sparse, grayish in SV; cheilocystidia moderately numerous, 48–73 × 8–12 μm, fusiform to subfusiform, occasionally subclavate to clavate; apex obtuse to papilliform; lamellar edges fertile. Pileipellis, two-layered, containing a suprapellis and subpellis unambiguously delimited from the subjacent context. Suprapellis, 100–150 μm thick with an ixotrichoderm in the center of the pileus, containing gelatinized, horizontal to oblique, infrequently vertical hyaline hyphae; apical cells 10–34(–43) × 4–9 μm, cylindrical to subcylindrical, sometimes subclavate or flexuous, apex obtuse, occasionally constricted; subapical cells 12–28 × 11–16 μm, cylindrical to subcylindrical, infrequently inflated. Suprapellis, a trichoderm at the pileus margin, containing erect to more or less tilted elements; apical cells 11–28 × 5–9 µm, cylindrical, subcylindrical to narrowly clavate, apex obtuse, sometimes ventricose; subapical cells 8–18 × 3–8 µm, cylindrical to subcylindrical, infrequently branched or inflated. Subpellis, a cutis 70–90 µm thick, composed of loosely interwoven, repand to oblique, subcylindrical to irregularly shaped, often inflated, hyaline hyphal cells, 3–8 µm in width; cystidioid cells infrequent. Pileocystidia dispersive in the suprapellis and subpellis, mostly 3–5 celled, cylindrical, infrequently fusiform, 5–10(–13) µm in width; contents granular, sparse and uneven, grayish in SV; pileocystidia in the pileus center mostly 1–3 celled, frequently clavate, other characters the same as those in the pileus margin. Clamp connections absent in all tissues.

Habit and habitat: solid to scattered in intermixed coniferous and broad-leaved forests of *Larix principis-rupprechtii*, *Picea meyeri*, *Pinus tabuliformis*, and *Quercus mongolica.*

Known distribution: northern China (Inner Mongolia Autonomous Region).

Additional specimens examined: China, Inner Mongolia Autonomous Region, Chifeng City, Ningcheng County, Heilihe National Nature Reserve, Sidaogou, 24 July 2005, T.Z. Liu (CFSZ2453); ibid, 16 July 2016, T.Z. Liu & Wulantuya (CFSZ10893); ibid, 17 July 2016, (CFSZ10912); ibid, 24 August 2019, T.Z. Liu & Y.N. Ren (CFSZ21181); ibid, 18 August 2019, (CFSZ20782); Chifeng City, Bairin Youqi, Saihanwula National Nature Reserve, Zhenggou, 2 September 2008, T.Z. Liu, H.M. Tian & C. Sun (CFSZ3848); Chifeng City, Harqin Qi, Ma’anshan Forest Park, 3 September 2019, T.Z. Liu & Y.M. Gao (CFSZ21512).

Notes: This species is a member of subsect. *Xerampelinae* Singer, sect. *Polychromae* for its context browning with age, mild taste, fishy odor, a suprapellis with non-incrusted pileocystidia, and the absence of primordial hyphae (Sarnari, 1998). ITS phylogenetic analyses showed that the *R*. *paraxerampelina* clade did not cluster with any other species in this subsection. Multi-gene analyses indicated that *R*. *paraxerampelina* clustered with the European *R. favrei* M.M. Moser and North American *R*. cf. *katarinae* Adamčík & Buyck, *R. flavobrunnescens* A. Kong & Buyck, and *R. madrensis* A. Kong & Buyck. *Russula favrei* can be distinguished from *R*. *paraxerampelina* as having a brownish ocher, pinkish-olivaceous brown, opaque or finely velutinous pileus, and 2–3 septate pileocystidia (Romagnesi, 1985; Sarnari, 2005). *R*. *katarinae* differs in having an orange to orange-yellow or light-yellow pileus, wider hymenial cystidia, 68–85 × 9–11 µm, and narrower pileocystidia, 5–6.5 µm in width (Adamčík et al., 2015). *R. flavobrunnescens* can be differentiated by its yellowish-brown pileus, shorter basidia, 34–46 × 9.5–13.5 µm, hymenial cystidia with long appendages, and coniferous forest habitat. *R. madrensis* can be distinguished from *R*. *paraxerampelina* by the presence of lower spore ornamentations, 0.3–0.5 µm high, narrower hymenial cystidia, 9.5–13 µm wide, and 1–2 celled pileocystidia (Adamčík et al., 2019).

The other subsect. *Xerampelinae* species with a reddish to purplish pileus can be distinguished by the presence of specific morphological characters: *R. amoenoides* Romagn. has a purplish red, pruinous, even furfuraceous pileus, and narrower basidia/pileocystidia, 10–12/4–7 µm wide (Romagnesi, 1985). *R. graveolens* Romell has a pileus that can be purple, brown, green, or yellow on sunny sites, longer hymenial cystidia up to 110 µm in length, and subulate to bulky terminal cells of the suprapellis. *R. pascua* (F.H. Møller & Jul. Schäff.) Kühner has brown and yellow coloration on the pileus surface, narrower hymenial cystidia of 9–11 µm, and a habitat of high-elevation pastureland (Sarnari, 2005). *R. sancti-pauli* A. Kong & Buyck has hymenial cystidia with an apex 1–3.5(–10.5) µm long, narrow 1–2 celled pileocystidia 4.5–7µm wide, and a habitat of *Pinus* forests (Adamčík et al., 2019). *R. subrubens* has a bronze-brown pileus, rarely marbled with yellow reticulated spore ornamentations, 1–3 celled, narrow pileocystidia 5–8 µm wide, and a habitat of willow forest. *R. xerampelina* has larger basdiomata up to 110 mm in diam., longer but slender hymenial cystidia 50–115 × 8–12 µm, and narrower pileocystidia 5–7 µm wide (Romagnesi, 1985; Sarnari, 2005).

*Russula prunicolor* C.Y. Niu, T.Z. Liu and G.J. Li, sp. nov.

**Figure 14:**
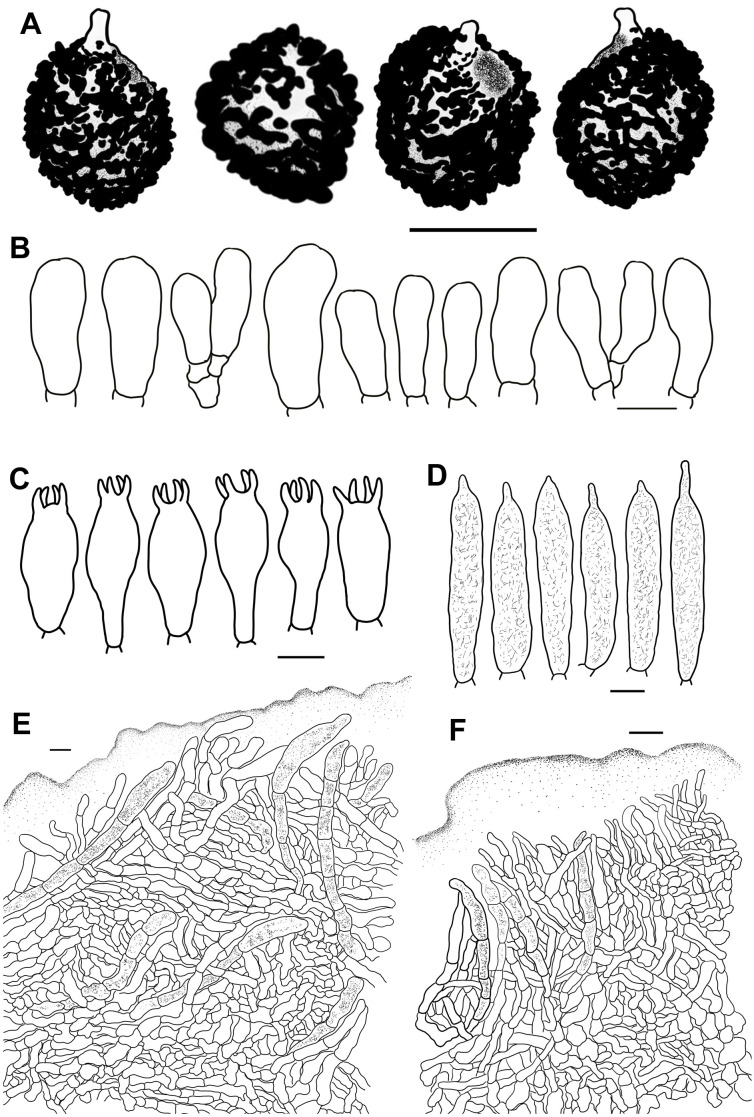
Line drawings of microscope images of *Russula prunicolor* (CFSZ19604, holotype). **(A)** basidiospores, **(B)** marginal cells, **(C)** basidia, **(D)** pleurocystidia, **(E)** suprapellis in the pileus center, **(F)** suprapellis at the pileus margin.

**Figures 2G**, **11E**, **14**.

Fungal Names FN 571966.

Diagnosis: Pileus 28–55 mm in diam., bright pinkish to reddish coloration, sometimes tinged with orange; margin subacute, but not striated. Lamellae adnate to somewhat adnexed, white, cream to ochraceous; edge even; lamellulae infrequent. Stipe 27–55 × 8–14 mm, cylindrical to subcylindrical, surface white, unchanging when bruised, rarely becoming pale ochraceous. Context, 2–3 mm thick at pileus center, white, cream to pale ochraceous; taste mild, odor indistinctly fishy. Spore print ocher. Basidiospores (6.1–) 6.4–8.8 (–9.3) × (4.8–) 5.1–7.5 (–7.8) μm, ornamentations 0.4–1.1 μm high, frequently linked by occasional to frequent fine lines; suprahilar spot large, amyloid. Basidia, 21–39 × 9–14 μm, clavate to subclavate, rarely cylindrical. Pleurocystidia 42–86 × 9–13 μm, cylindrical, subcylindrical to subclavate; apex often lanceolate to papilliform, infrequently bluntly acuminate; cheilocystidia not observed. Pileipellis composed of suprapellis and subpellis. Suprapellis an ixotrichoderm at the pileus center and margin. Pileocystidia present in the suprapellis and subpellis, multi-septate, cylindrical, 7–12 µm in width. Habitat in broad-leaved forests of *Quercus*.

Etymology: The epithet ‘*prunicolor*’ specifically refers to the color of the *Prunus persica* flower.

Holotype: from China’s Inner Mongolia Autonomous Region, Chifeng City, Harqin Qi, Meilin Township, Taipingzhuang Village, 6 August 2018, T.Z. Liu, Y.Q. Guan & N. Liu (CFSZ19604).

Description: Basidiomata small- to medium-sized, rarely very small. Pileus, 28–55 mm in diam., hemispheric when young, then turning convex, plano-convex to planate, rarely depressed at the center, viscid to glutinous when wet, glabrous, smooth; tinged with bright pinkish to reddish, sometimes with orange coloration; Dragon’s Blood Red (XIII5′i), Coral Red (XIII5′) to Light Coral Red (XIII5′b) at the center, often faded to Coral Pink (XIII5′d), Strawberry Pink (I5d) to Shrimp Pink (I5f) towards the edge; margin subacute, incurved to outstretched, occasionally wavy and cracked, not striated, peeling 1/5–1/3 towards the center. Lamellae, adnate to somewhat adnexed, 2–5 mm in height at mid-point of radius, occasionally forked near the stipe attachment, faintly interveined, White (LIII) when young, becoming cream to ochraceous at maturity, Light Buff (XV17′f), Massicot Yellow (XVI21′f) to Cream Color (XVI19′f), Naples Yellow (XVI19′d) to mustard yellow (XVI19′b) when bruised; edge even, 14–20 lamellae per cm along the pileus margin; lamellulae infrequent. Stipe, central, 27–55 × 8–14 mm, cylindrical to subcylindrical, rarely subclavate or ventricose towards the base, smooth, turning longitudinally rugulose when mature, surface white (LIII), unchanging when bruised, rarely becoming pale ochraceous, of a Cream Buff (XXX19′′d) or Chamois (XXX19′′b) color, solid when young, turning tubular to hollow with age. Context, 2–3 mm thick at pileus center, White (LIII), usually unchanging, but in rare cases slowly becoming cream-colored to pale ochraceous of a Light Ochraceous Buff (XV15′d) to Ochraceous Buff (XV15′b) color when bruised, brittle; taste mild, odor indistinctly fishy. Spore print, ocher (IIIa–IIIb).

Basidiospores, [100/2/2] (6.1–) 6.4–8.8 (–9.3) × (4.8–) 5.1–7.5 (–7.8) μm, Q = (1.02–) 1.05–1.38 (–1.44), (Q = 1.20 ± 0.09), subglobose, broadly ellipsoid to ellipsoid, rarely globose, ornamentations composed of moderately distant to dense [(6–)7–9(–10)], cylindrical, subcylindrical to subconical amyloid warts, 0.4–1.1 μm in height, frequently to abundantly fused in pairs and long branched chains [3–4(–5) fusions in the circle], frequently linked by occasional to frequent fine lines [2–3(–4) in the circle]; suprahilar spot large, amyloid, embossed, partly merged into ornamentations on edge. Basidia 21–39 × 9–14 μm, clavate to subclavate, rarely cylindrical, hyaline; sterigmata, 4–7 μm, often slightly incurved, occasionally straight. Marginal cells 17–27 × 6–12 μm, clavate to subclavate, rarely subcylindrical, sometimes flexuous. Hymenial cystidia pleurocystidia widely dispersed, 100–220/mm^2^, 42–86 × 9–13 μm, cylindrical, subcylindrical to subclavate, projecting 10–40 μm beyond hymenium; apex often lanceolate to papilliform, infrequently bluntly acuminate; contents crystalline, sparse, grayish in SV; cheilocystidia not observed; lamellar edges fertile. Pileipellis composed of suprapellis and subpellis, somewhat ambiguously distinguishable from the underlying context tissue. Suprapellis, 110–140 μm thick, with an ixotrichoderm at the center of the pileus composed of gelatinized, mostly oblique, infrequently repand and erect hyaline hyphae; terminal cells, 12–32 × 4–10 µm, subcylindrical, subclavate to clavate, occasionally ventricose or branched, apex obtuse; subapical cells, 10–18 × 3–8 µm, cylindrical, rarely inflated. Suprapellis has an ixotrichoderm at the pileus margin and contains more vertical elements; apical cells 9–23(–27) × 3–8 µm, subcylindrical, infrequently flexuous or constricted at apex; subapical cells 8–17 × 4–8 µm, cylindrical to subcylindrical, rarely branched. Subpellis, a cutis 80–100 µm thick, composed of loosely interlaced, flexuous, subglobose to irregularly shaped hyphal cells, sometimes inflated; cystidioid elements rare. Pileocystidia in the pileus margin present in the suprapellis and subpellis, multi-septate, cylindrical, 7–12 µm in width; apex obtuse; contents granular to crystalline, uneven, grayish in SV; pileocystidia in the pileus center 7–13 µm in width; apex obtuse to bluntly acuminate, other characters the same as those in the pileus margin. Clamp connections absent in all tissues.

Habit and habitat: dense to scattered in broad-leaved forests of *Quercus aliena*, *Q. dentata*, *Q. mongolica*, *Q. variabilis* and *Q. wutaishansea.*

Known distribution: northern China, Hebei Province, Inner Mongolia Autonomous Region.

Additional specimens examined: China, Hebei Province, China: Baoding City, Laishui County, Jiulongcheng Township, Beibianqiao Village, Baicaopan Forest Park, 16 August 2021, S.Y. Zhao, C.Y. Niu, S. Chen, X.L. Gao & G.J. Li, 20210438 (HBAU15884); ibid, 18 August 2022, G.J. Li, 20211463 (HBAU15883).

Notes: The phylogenetic assignments of *R*. *prunicolor* are the same as those of *R*. *paraxerampelina.* A BLAST search of the ITS sequence and phylogenetic analysis indicated that this species has a close relationship with *R. graveolens*. These two species can be distinguished by the presence of yellow and ochraceous tinges on the pileus surface, basidiospores with isolated warts and spines, longer basidia/hymenial cystidia, up to 60/110 µm, and apically attenuated, subulate, fusiform or lageniform terminal cells in the suprapellis of *R. graveolens* (Romagnesi, 1985; Sarnari, 2005; Adamčík et al., 2016b). The ITS phylogeny also indicated that *R*. *prunicolor* was closely related to the undescribed taxon, *R.* cf. *amoenoides*. A comparative analysis was not possible because of the insufficient type description of *R.* cf. *amoenoides* (Adamčík et al., 2016b).

The other red-capped member of subsect. *Xerampelinae* species can be distinguished from *R*. *prunicolor* as follows: *R. amoenoides* has longer basidia, 40–50 × 10–12 µm, and narrower pileocystidia, 4–7 µm in width (Romagnesi, 1985; Sarnari, 2005); *R. katarinae* has spore ornamentation composed of narrower pileocystidia, mostly linked by fine lines, 4.5–7 µm in width, and a habitat of *Pinus* forests (Adamčík et al., 2015). *R. madrensis* has low spore ornamentations 0.3–0.5 µm high, longer basidia, (40–)42–48(–53) × 11–13(–14.5) µm, and a coniferous forest habitat of *Picea* and *Pinus* (Adamčík et al., 2019). *R. pascua* has shades of brown, yellow, or olivaceous on the pileus surface, narrower pileocystidia 5–8 µm in width, and a habitat of high elevation pastureland (Romagnesi, 1985; Sarnari, 2005). *R. sancti-pauli* has larger basidiospores, (7.6–) 8.4–9.8 (–10.6) × (4.8–) 5.1–7.5 (–7.8) μm, longer basidia, (42–)44.5–55.5(–60) × (11–)11.5–13.5(–15) µm, and a habitat of *Pinus* forests (Adamčík et al., 2019). *R. subrubens* has tinges of yellow to ochraceous on the pileus, longer hymenial cystidia 62–100 × 10–13.5 μm in length, and a habitat of *Salix* forests. *R. xerampelina* has larger basidiospores, 8–11 × 6.5–8.2 µm, longer basidia of 40–60 × 10–15 µm, narrower pileocystidia 5–8 µm in width, and a coniferous forest habitat of *Larix* and *Picea* (Romagnesi, 1985; Sarnari, 2005).

There is an unidentified *Russula* sample from Thailand (TUF 116539, UDB025298) in the *R*. *prunicolor* complex clade. This specimen may represent a closely related, but different, undescribed species because there are significant variations in geographical location, climate, vegetation form, and flora between North China and Thailand. This can be explained by the theory that the speciation of ectomycorrhizal fungi such as the *Russula* species is driven by host-switch events (Looney et al., 2016; Feng and Yang, 2019).

*Russula subrubens* (J.E. Lange) Bon, Docums Mycol. [2] (no. 5): 33, 1972.

**Figure 15:**
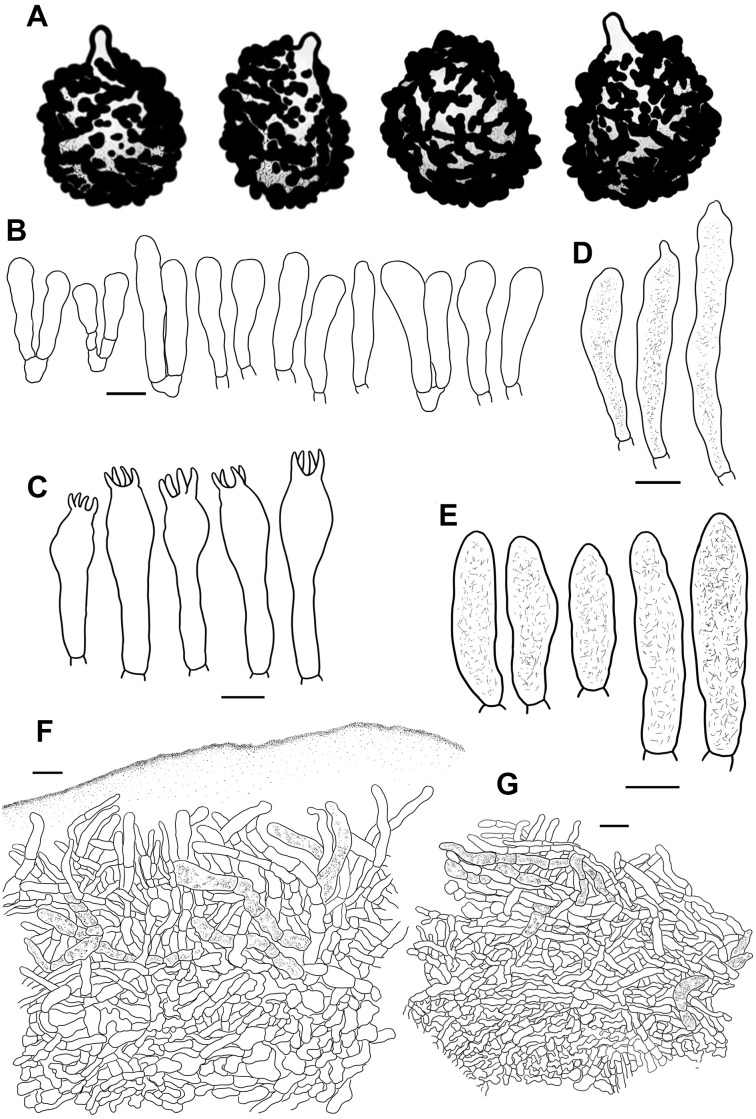
Line drawings of microscope images of *Russula subrubens* (CFSZ12356). **(A)** basidiospores, **(B)** marginal cells; **(C)** basidia, **(D)** cheilocystidia, **(E)** pleurocystidia, **(F)** suprapellis in the pileus center, **(G)** suprapellis at the pileus margin.

**Figures 2H**, **12F**, **15**.

MycoBank MB 322966.

Description: Basidiomata small- to medium-sized. Pileus 35–58 cm in diam., initially plano-hemispheric to umbonate, expanding to pulvinate, plano-convex, and planate to slightly concave when mature, glutinous when wet, glabrous, smooth, rarely flaking in small patches; mainly pale yellowish to ochraceous, often tinged with helvus to orangish colors, Capucine Orange (III15d), Orange Buff (III15d) to yellow ocher (XV17′) at center, Ochraceous Buff (XV15′b), Light Ochraceous Buff (XV15′d) to Capucine Buff (III13f) towards the edge; margin subacute, initially aduncal, then flat when mature, indistinctly fluctuant, occasionally cracked, non-striated, peeling 1/5–1/4 towards the center. Lamellae adnate when young, adnexed with age, 2–4 mm in height at the mid-point of the pileus radius, occasionally forking near the stipe attachment and edge, slightly veined, initially white (LIII), becoming cream to pale ochraceous with tinges of Light Buff (XV17′f), Pale Ochraceous Buff (XV15′f) to Cream Color (XVI19′f); edge even, 13–19 blades in 1 cm of the pileus margin, lamellulae present. Stipe central, 47–64 × 9–16 mm, clavate to subclavate, sometimes subcylindrical, slightly tapered at the lower end, annulus absent, initially smooth, then longitudinally rugulose with age, surface white (LIII), unchanging or becoming pale yellowish to ochraceous with tinges of Cream Buff (XXX19′′d), Antimony Yellow (XV17′b) to Yellow Ocher (XV17′) at base when bruised, stuffed and solid at first, then becoming tubercular to hollow at maturity. Context 2–4 mm thick at the pileus center, White (LIII) at first, unchanging or becoming cream colored with tinges of Massicot Yellow (XVI21′f) to Naphthalene Yellow (XVI23′f) when old, occasionally becoming Capucine Buff (III13f) to Pale Yellow Orange (III15f) when bruised; no distinct taste or odor. Spore print ocher (IIIb–IIIc).

Basidiospores [100/2/2] (7.0–) 7.5–9.7 (–10.0) × (5.5–) 6.0–7.8 (–8.1) μm, Q = (1.02–) 1.08–1.39 (–1.45), (Q = 1.23 ± 0.08), subglobose, broadly ellipsoid to ellipsoid, occasionally globose, ornamentations moderately distant to dense (5–9 in a 3 μm diam. circle), verrucose, subcylindrical to cylindrical, rarely subconical amyloid warts 0.3–0.7 μm in height, reticulate, occasionally to frequently fused as long ridges and crests [1–3(–4) fusions in the circle], linked by frequent to abundant fine lines [(0–)1–3(–5) in the circle]; suprahilar spot large, amyloid, smooth to slightly verrucose, often radically projected in edge. Basidia 33–49 × 10–15 μm, subclavate to subcylindrical, infrequently clavate, four-spored, hyaline; sterigmata 3–7 μm, often straight to slightly incurved. Marginal cells 16–37 × 5–11 μm, mostly subclavate to subcylindrical, infrequently clavate and cylindrical. Hymenial cystidia pleurocystidia dispersed to moderately numerous, 300–1000/mm^2^, 36–80 × 7–13 μm, cylindrical to subcylindrical, occasionally subfusiform, projecting 15–40 μm beyond the hymenium; apex obtuse, contents crystalline, sparsely distributed, grayish in SV; cheilocystidia dispersed to moderately numerous, 41–73 × 7–11 μm; apex obtuse, bluntly acuminate to papilliform; lamellar edges fertile. Pileipellis two-layered, composed of suprapellis and subpellis, with diffuse delimitation between pileipellis and context spherocytes. Suprapellis 60–90 μm thick, an ixotrichoderm in the pileus center, composed of gelatinized, tightly interlaced, vertical to oblique, rarely repand hyaline hyphae; apical cells 8–30(–35) × 3–6 μm, cylindrical to subclavate, sometimes constricted or flexuous near the apex; subapical cells 9–17 × 3–6 μm, cylindrical, infrequently inflated or branched. Suprapellis in the pileus margin a trichoderm containing mostly horizontal to tilted, infrequently vertical hyaline hyphae; terminal cells 8–23 × 3–6 μm, cylindrical, rarely flexuous; apex obtuse; subapical cells 8–18 × 3–5 μm, cylindrical to subcylindrical, infrequently branched. Subpellis 70–110 μm thick, a cutis, composed of subcylindrical, often inflated to irregularly shaped hyphal cells, 4–10 μm in width; cystidioid elements infrequent. Pileocystidia in the pileus margin abundant in suprapellis, rarely arising from the subpellis, multi-septate, sometimes single-celled, cylindrical to subclavate, 5–9 μm in width; apex obtuse, occasionally constricted; contents granular, heterogeneous, partly crystalline and sparse, pale grey in SV; pileocystidia in pileus center 5–11 μm in width, other characters the same as those in the pileus margin. Clamp connections absent in all tissues.

Habit and habitat: dense to scattered in riparian broad-leaved forests of *Salix schwerinii* E.L. Wolf.

Known distribution: northern China (Inner Mongolia Autonomous Region), Austria, Denmark, France, Italy, and Norway.

Additional specimens examined: China, Inner Mongolia Autonomous Region: Chifeng City, Bairin Youqi, Saihanwula National Nature Reserve, Wangfengou, 13 September 2016, T.Z. Liu & Z.L. Song (CFSZ12356); ibid, 26 July 2017, T.Z. Liu, Wulantuya & Zhaorigetu (CFSZ12952); Hulun Buir City, Ergun City, Labudalin Subdistrict, 6 August 2022, T.Z. Liu, W.L. Hua & Y.M. Gao (CFSZ25048); ibid (CFSZ25050).

Notes: This species is described based on samplings from Denmark. Its habitat is well-characterized as primarily *Salix* spp. in subsect. *Xerampelinae*. Most of the morphological characters of *R. subrubens* collected in China were in accordance with those of European samplings. The latter have minor differences such as more reddish coloration on the pileus surface and longer basidia/hymenial cystidia (50–63 × 10–14/62–100 × 10–13.5 μm) (Sarnari, 2005). Subtle habit and habitat differences were also found in collections from various regions. The European specimens were collected among low shrubs of *Salix herbacea*, whereas the Chinese ones were found among tall shrubs or small trees of *S*. *schwerinii*. Further multi-gene phylogenetic analyses of European samplings of this species are still needed. Multi-gene phylogenetic analyses indicated that *R. subrubens* was closely related to *R. prunicolor* and several North American subsect. *Xerampelinae* members (**Figure 1**).

## Discussion

The concept of a crown clade in this study is similar to that of Looney et al. (2016) and Clade VIII in Buyck et al. (2018). Previous analyses have revealed a high species diversity in this group (Miller and Buyck, 2002; Li, 2014; Looney et al., 2016; Buyck et al., 2018; Adamčík et al., 2019). Sectional and subsectional infrageneric classification of *Russula* genera has not been comprehensively updated based on morphology molecular phylogeny and ecological habit. When the new infrageneric taxon concepts are employed, the new species and Chinese records in this analysis belonged to five subsections of four sections.

Section *Amethystinae* contains subsect. *Amethystinae*, subsect. *Chamaeleontinae*, subsect. *Integroidinae* Romagn, and subsect. *Olivaceinae* following the morphological classification of Sarnari (1998). The multi-gene phylogenetic analyses of this study indicated a narrower range of sect. *Amethystinae*. Three lineages of *R. amethystina*, *R. burlinghamiae*, and *R*. *risigallina* generally corresponded with subsect. *Amethystinae*, subsect. *Alboflavinae*, and subsect. *Chamaeleontinae* (**Figure 1**). Members of subsect. *Olivaceinae* and subsect. *Integroidinae* did not cluster with these three lineages. The monophyly of subsect. *Chamaeleontinae* was not supported in the ITS phylogeny; for example, the *R. plana* complex did not cluster with that of *R*. *risigallina* (**Supplementary Figure 1**). The species of the newly established subsect. *Alboflavinae* all grow in broad-leaved forest habitats, which clearly distinguished them from the coniferous habitats of subsect. *Amethystinae* (Sarnari, 1998, 2005). The habitat of subsect. *Chamaeleontinae* is somewhat complicated, especially for the *R*. *risigallina* complex (**Supplementary Figure 1**). *Russula chrysantha*, *R. helios*, and *R. gilva* grow in broad-leaved forests (Romagnesi, 1985; Siquier et al., 2015), while *R. brunneopurpurea* and *R. postiana* favor coniferous forest habitats (Romell, 1911; Ruotsalainen and Vauras, 1990; Jabeen et al., 2017). The basidiomata of *R*. *flaviceps*, *R*. *risigallina*, and *R. vitellina* were collected from intermixed or undetailed forest habitats (Romagnesi, 1985; Sarnari, 2005; Adamčík et al., 2013). These indicated that the symbiotic tree species of subsect. *Chamaeleontinae* members are still undefined. It should be noted that a large proportion of the *R*. *risigallina* complex members from different continents had >98% similarity in the ITS region. This may indicate a recent or ongoing allopatric speciation in various habitats and regions. The concrete phylogenetic positions of several sect. *Amethystinae* species still remain unsolved in current and previous ITS phylogenetic analyses. These species include *R. clavatohyphata* R.P. Bhatt, A. Ghosh, Buyck & K. Das from *Pinus* forests (Wang et al., 2019), *R. uttarakhandia* A. Ghosh & K. Das in mixed forests (Phookamsak et al., 2019), and a gasteroid taxon, *R. chlorineolens* Trappe & T.F. Elliott under *Picea* and *Tsuga* (Smith, 1963; Xu et al., 2019); therefore, multi-gene analyses are necessary for the identification and phylogeny of these species.

Our multi-gene phylogenetic analyses showed that the three lineages of *R. laricina*, *R. melliolens*, and *R. puellaris* clustered together and formed a well-supported clade (MLBS 83, BSPP 1, **Figure 1**). This supported the hypothesis that sect. *Tenellae* contained subsect. *Melliolentinae* (Singer) Sarnari., subsect. *Laricinae*, and subsect. *Puellarinae* Singer. The monophyly of subsect. *Puellarinae* was not supported in the ITS phylogeny (**Supplementary Figure 2**). This could explain the unresolved phylogenetic positions of some sect. *Tenellae* species such as *R. coronaspora* Y. Song and *R. minor* Y. Song in their original descriptions (Song et al., 2021). Our multi-gene phylogenetic topology indicated that *R. coronaspora* and *R. minor* correspondingly belong to subsect. *Puellarinae* and subsect. *Melliolentinae*. The phylogenetic backbone of subsect. *Puellarinae* still remains largely unresolved because only limited sequences were available for the multi-gene analyses. Subsection *Laricinae* was well supported in the ITS phylogeny with high species diversity (MLBS 88, MPBS 89, BSPP 1, **Supplementary Figure 2**). The regularity of habitat can be summarized for the three main clades in this subsection. Species in clades 1 and 2 grow in broad-leaved forests, while clade 3 members of the *R. laricina* complex prefer coniferous habitats (**Supplementary Figure 2**). *Russula graminea* Ruots., H.-G. Unger & Vauras may be an exception to this regularity. It is reported that this species grows in old forests of *Picea abies*, whereas scattered *Alnus incana*, *Betula pubescens*, and abundant *Vaccinium myrtillus* were recorded at sampling sites (Vauras et al., 2012). It should be noted that *R. font-queri* and *R. liyui* of subsect. *Laricinae* were barely distinguishable in the ITS phylogeny (**Supplementary Figure 2**). The 99% similarity of these ITS regions is reminiscent of the gasteroid *R. sichuanensis* and agaricoid *R. vinosobrunneola* also in subsect. *Laricinae* (Li et al., 2018a). In our opinion, it is incorrect to classify the agaricoid samplings from Pakistan as *R. sichuanensis* because of obvious morphological differences. These agaricoid collections may represent an unknown species because they can also be distinguished from *R. vinosobrunneola* as having larger basidiospores of 8–12 × 7–10 µm, with higher warts up to 1.5 µm, wider basidia/pleurocystidia of 11–17/8–16 µm, and an ixotrichoderm pileipellis (Saba and Khalid, 2015).

The ITS phylogeny of this study manifested a high species diversity in subsect. *Olivaceinae*. It should be noted that the clades of the *R. olivacea* samples in Eberhardt (2002), Miller and Buyck (2002), and Caboň et al. (2017) did not cluster together (**Supplementary Figure 3**). This indicated that for the concrete phylogenetic species concept of *R. olivacea*, the type of species of subsect. *Olivaceinae* still remain unsolved. There is no doubt that the Asian and North American samples labeled as *R. olivacea* in **Supplementary Figure 3** are misidentified. Similar disagreements also occurred in complexes of *R. alutacea* and *R. vinososordida*. Only three species, *R*. *alutacea*, *R*. *olivacea*, and *R. vinosobrunnea* (Bres.) Romagn., were subsumed into subsect. *Olivaceinae* based on morphology (Sarnari, 2005). Molecular phylogenetic results supported the hypothesis that *R. decolorans* (Fr.) Fr., *R. firmula* Jul. Schäff., *R. rivulicola* Ruots. & Vauras, and *R. vinososordida* Ruots. & Vauras are members of this subsection (**Supplementary Figure 3**). *Russula decolorans*, *R. rivulicola*, and *R. vinososordida* belong to subsect. *Integriforminae* (Bon) Sarnari, of sect. *Polychromae*. *Russula firmula* is a member of subsect. *Urentes* Maire, sect. *Russula* (Romagn.) Sarnari following the infrageneric taxonomy of Sarnari (1998, 2005). The topology of the ITS phylogenetic analyses indicated three main clades in subsect. *Olivaceinae* (**Supplementary Figure 3**). It can be seen that these clades inhabit different forest types. Species in clades 1 and 3 are found primarily in broad-leaved forests dominated by *Betula* and Fagaceae, respectively. Clade 2 members grow in coniferous forest mainly composed of *Abies*, *Picea*, and *Pinus* (Romagnesi, 1985; Sarnari, 2005). The basal multi-gene phylogenetic relationships of subsect. *Olivaceinae* are unclear because of the limited number of species sampled (**Figure 1**).

The taxonomy and phylogeny of subsect. *Xerampelinae* have been well analyzed in a series of studies in recent decades (Adamčík and Marhold, 2000; Adamčík, 2002, 2004; Adamčík and Knudsen, 2004; Adamčík and Buyck, 2010, 2011; Adamčík et al., 2016b; Noffsinger et al., 2024). Most of these analyses were carried out based on European and North American specimens, with few Asian samples being cited. The basal phylogenetic relationships of subsect. *Xerampelinae* could be clarified if their ITS sequences were analyzed (**Supplementary Figure 4**). Most of the highly supported clades in the ITS and multi-gene phylogenies in this study corresponded to those of Noffsinger et al. (2024). Clades 1, 2, and 3 in **Figure 1** are associated with clades of *R. graveolens*, *R. clavipes*, and *R. xerampelina* in Noffsinger et al. (2024). Summarizing the main forest habitats of these three clades, most species of clade 1 were from Fagaceae forests, while clade 2 members generally associated with trees of *Betula* and *Salix*. Taxa from clade 3 preferred coniferous forest dominated by *Picea* and *Pinus*. A possible exception for *R. paraxerampelina* in clade 3 should be noted because *Quercus* species have always been recorded at its collection sites. The ITS phylogeny of this analysis (**Supplementary Figure 4**) supported the point that some subsect. *Xerampelinae* species, e.g., *R. favrei* (known as *R*. *serissima* Peck in Noffsinger et al., 2024), *R. nuoljae*, and *R. subrubens*, may have a Holarctic distribution from Europe to North Asia, whereas *R. paraxerampelina* is probably endemic to Asian regions. The distribution of *R. nuoljae* in northern China has been continuously reported in recent years based on morphology and ITS evidence (Liu et al., 2017; Cao et al., 2019; Wei and Liu, 2019; Hu, 2020; Li, 2021; Wang et al., 2024). The *R. favrei* samples used in the ITS phylogenetic analyses (**Supplementary Figure 4**) were obtained in the Xinjiang Uygur Autonomous Region of northwest China, as well as central Sikhote-Alin of eastern Siberia. Morphological observations and multi-gene phylogenetic analyses are still needed to clarify the distribution of this species in Asia.

## Data Availability

The datasets presented in this study can be found in online repositories. The names of the repository/repositories and accession number(s) can be found in the article/**Supplementary Material**.

## Supplementary material

Supplementary Table 1 Sampling site information of the newly collected specimens in this study.

Supplementary Table 2 Species information and GenBank accession numbers of the DNA sequences in the multi-gene phylogenetic analyses of the *Russula* crown clade.

Supplementary Table 3 Species information and GenBank/UNITE accession numbers of the DNA sequences in the ITS phylogenetic analyses of sect. *Amethystinae*.

Supplementary Table 4 Species information and GenBank/UNITE accession numbers of the DNA sequences in the ITS phylogenetic analyses of subsect. *Laricinae*.

Supplementary Table 5 Species information and GenBank/UNITE accession numbers of the DNA sequences in the ITS phylogenetic analyses of subsect. *Olivaceinae*.

Supplementary Table 6 Species information and GenBank/UNITE accession numbers of the DNA sequences in the ITS phylogenetic analyses of subsect. *Xerampelinae*.

Supplementary Table 7 Results obtained by ITS sequence GenBank BLAST tests.

Supplementary Figure 1 Maximum likelihood (ML) phylogenetic tree of sect. *Amethystinae* based on ITS region. Bootstrap values ≥75% of ML and MP, as well as posterior probabilities ≥0.9 are presented above the clades as MLBS/MPBS/PP. Labels in bold represented new collections for this analysis.

Supplementary Figure 2 Maximum likelihood (ML) phylogenetic tree of subsect. *Laricinae* based on ITS region. Bootstrap values ≥75% of ML and MP, as well as posterior probabilities ≥ 0.9 are presented above the clades as MLBS/MPBS/PP. Labels in bold represented new collections for this analysis.

Supplementary Figure 3 Maximum likelihood (ML) phylogenetic tree of subsect. *Olivaceinae* based on ITS region. Bootstrap values ≥75% of ML and MP, as well as posterior probabilities ≥0.9 are presented above the clades as MLBS/MPBS/PP. Labels in bold represented new collections for this analysis.

Supplementary Figure 4 Maximum likelihood (ML) phylogenetic tree of subsect. *Xerampelinae* based on ITS region. Bootstrap values ≥75% of ML and MP, as well as posterior probabilities ≥ 0.9 are presented above the clades as MLBS/MPBS/PP. Labels in bold represented new collections for this analysis.

## References

[B1] Adamčík S. (2002). Taxonomy of the *Russula xerampelina* group. Part 2. Taxonomic and nomenclatural study of *Russula xerampelina* and *R. erythropoda* . Mycotaxon 82, 241–267.

[B2] Adamčík S. (2004). Studies on *Russula clavipes* and related taxa of *Russula* section *Xerampelinae* with a predominantly olivaceous pileus. Persoonia 18, 393–409.

[B3] Adamčík S. Buyck B. (2010). Re-instatement of *Russula levyana* Murrill as a good and distinct American species of *Russula* section *Xerampelinae* . Cryptogam. Mycol. 31, 119–135.

[B4] Adamčík S. Buyck B. (2011). The species of Russula subsection Xerampelinae described by C.H. Peck and Miss G.S. Burlingham. Cryptogam. Mycol. 32, 63–81. doi: 10.7872/crym.v32.iss1.2012.063

[B5] Adamčík S. Knudsen H. (2004). Red-capped species of *Russula* sect. *Xerampelinae* associated with dwarf scrub. Mycol. Res. 108, 1463–1475. doi: 10.1017/S0953756204000875 15757183

[B6] Adamčík S. Marhold K. (2000). Taxonomy of the *Russula xerampelina* group. I. Morphometric study of the *Russula xerampelina* group in Slovakia. Mycotaxon 76, 463–479.

[B7] Adamčík S. Caboň M. Eberhardt U. Saba M. Hampe F. Slovak M.. (2016a). A molecular analysis reveals hidden species diversity within the current concept of *Russula maculata* (Russulaceae, Basidiomycota). Phytotaxa 270, 71–88. doi: 10.11646/phytotaxa.270.2.1

[B8] Adamčík S. Cai L. Chakraborty D. Chen X. H. Cotter H. V. T. Dai D. Q.. (2015). Fungal biodiversity profiles 1-10. Cryptogam. Mycol. 36, 121–166. doi: 10.7872/crym/v36.iss2.2015.121

[B9] Adamčík S. Carteret X. Buyck B. (2013). Type studies on some *Russula* species described by C.H. Peck. Cryptogam. Mycol. 40, 57–95. doi: 10.7872/crym.v34.iss2.2013.367

[B10] Adamčík S. Slovák M. Eberhardt U. Ronikier A. Jairus T. Hampe F.. (2016b). Molecular inference, multivariate morphometrics and ecological assessment are applied in concert to delimit species in the *Russula clavipes* complex. Mycologia 108, 716–730. doi: 10.3852/15-194 27091390

[B11] Adamčík S. Looney B. Caboň M. Jančovičová S. Adamčíková K. Avis P. G.. (2019). The quest for a globally comprehensible *Russula* language. Fungal Divers. 99, 369–449. doi: 10.1007/s13225-019-00437-2

[B12] Bazzicalupo A. L. Buyck B. Saar I. Vauras J. Carmean D. Berbee M. L. (2017). Troubles with mycorrhizal mushroom identification where morphological differentiation lags behind barcode sequence divergence. Taxon 66, 791–810. doi: 10.12705/664.1

[B13] Buyck B. Halling R. E. Mueller G. M. (2003). Inventario del genere *Russula* in Costa Rica: ritrovamento di due specie nordamericane molto rare nelle foreste di quercia di montagna. Bollettino. del Gruppo. Micologico. “G. Bresadola”. 46, 57–74.

[B14] Buyck B. Hofstetter V. Eberhardt U. Verbeken A. Kauff F. (2008). Walking the thin line between *Russula* and *Lactarius*: the dilemma of *Russula* subsect. *Ochricompactae* . Fungal Divers. 28, 15–40.

[B15] Buyck B. Thoen D. Watling R. (1996). Ectomycorrhizal fungi of the Guinea-Congo region. P. R. Soc Edinb. A. 104, 313–333. doi: 10.1017/S0269727000006175

[B16] Buyck B. Zoller S. Hofstetter V. (2018). Walking the thin line … ten years later: the dilemma of above- versus below-ground features to support phylogenies in the Russulaceae (Basidiomycota). Fungal Divers. 89, 267–292. doi: 10.1007/s13225-018-0397-5

[B17] Caboň M. Eberhardt U. Looney B. Hampe F. Kolařík M. Jančovičová S.. (2017). New insights in *Russula* subsect. *Rubrinae*: phylogeny and the quest for synapomorphic characters. Mycol. Prog. 16, 877–892. doi: 10.1007/s11557-017-1322-0

[B18] Caboň M. Li. G. J. Saba M. Kolařík M. Jančovičová S. Khalid A. N.. (2019). Phylogenetic study documents different speciation mechanisms within the *Russula globispora* lineage in boreal and arctic environments of the Northern Hemisphere. IMA. Fungus. 10, 5. doi: 10.1186/s43008-019-0003-9 32647614 PMC7325667

[B19] Calonge F. D. Martín M. P. (2000). Morphological and molecular data on the taxonomy of *Gymnomyces*, *Martellia* and *Zelleromyces* (Russulales). Mycotaxon 76, 9–15.

[B20] Cao B. Li G. J. Zhao R. L. (2019). Species diversity and geographic components of *Russula* from the Greater and Lesser Khinggan Mountains. Biodivers. Sci. 27, 854–866. doi: 10.17520/biods.2019040

[B21] Crous P. W. Wingfield M. J. Lombard L. Roets F. Swart W. J. Alvarado P.. (2019). Fungal Planet description sheets: 951–1041. Persoonia 43, 223–425. doi: 10.3767/persoonia.2019.43.06 32214501 PMC7085856

[B22] Dai Y. C. Zhou L. W. Yang Z. L. Wen H. A. Bau T. Li T. H. (2010). A revised checklist of edible fungi in China. Mycosystema 29, 1–21.

[B23] Dugan F. M. (2011). Conspectus of world ethnomycology (St. Paul: American Phytopathological Society).

[B24] Durall D. M. Gamiet S. Simard S. W. Kudrna L. Sakakibara S. M. (2006). Effects of clearcut logging and tree species composition on the diversity and community composition of epigeous fruit bodies formed by ectomycorrhizal fungi. Can. J. Bot. 84, 966–980. doi: 10.1139/b06-045

[B25] Eberhardt U. (2002). Molecular kinship analyses of the agaricoid Russulaceae: correspondence with mycorrhizal anatomy and sporocarp features in the genus *Russula* . Mycol. Prog. 1, 201–223. doi: 10.1007/s11557-006-0019-6

[B26] Edler D. Klein J. Antonelli A. Silvestro D. (2020). raxmlGUI 2.0: A graphical interface and toolkit for phylogenetic analyses us-ing RAxML. Methods Ecol. Evol. 12, 1–5. doi: 10.1111/2041-210X.13512

[B27] Felsenstein J. (1985). Confidence intervals on phylogenetics: an approach using bootstrap. Evolution 39, 783–791. 28561359 10.1111/j.1558-5646.1985.tb00420.x

[B28] Feng B. Yang Z. L. (2019). Diversity of mycobionts and molecular mechanisms that entail the development of ectomycorrhizae (in Chinese). Sci. Sin. Vitae. 49, 436–444. doi: 10.1360/N052018-00220

[B29] Geml J. Laursen G. A. Timling I. Mcfarland J. M. Booth M. G. Lennon N.. (2009). Molecular phylogenetic biodiversity assessment of arctic and boreal ectomycorrhizal *Lactarius* Pers. (Russulales; Basidiomycota) in Alaska, based on soil and sporocarp DNA. Mol. Ecol. 18, 2213–2227. doi: 10.1111/j.1365-294X.2009.04192.x 19389163

[B30] Geml J. Taylor D. L. (2013). Biodiversity and molecular ecology of *Russula* and *Lactarius* in Alaska based on soil and sporocarp DNA sequences. Scripta. Botanica Belgica. 51, 132–145.

[B31] Ghosh A. Buyck B. Chakraborty D. Hembrom M. E. Bera I. Das K. (2023). Three new species of genus *Russula* Pers. from Sal dominated forests of tropical India based on morphotaxonomy and multigene phylogenetic analysis. Cryptogam. Mycol. 44, 27–50. doi: 10.5252/cryptogamie-mycologie2023v44a3

[B32] Ghosh A. Das K. Buyck B. (2021a). Two new species in the *Russula* (Russulaceae, Basidiomycota) crown clade from Indian Himalaya. Eur. J. Taxonomy. 782, 157–172. doi: 10.5852/ejt.2021.782.1595

[B33] Ghosh A. Das K. Chakraborty D. (2021b). Morphology and molecular approach reveal a new species of the genus *Russula* subsect. *Lepidinae* (Russulaceae) from India. Phytotaxa 483, 244–254. doi: 10.11646/phytotaxa.483.3.4

[B34] Guo J. Y. Karunarathna S. C. Mortimer P. E. Xu J. C. Hyde K. D. (2014). Phylogenetic diversity of *Russula* from Xiaozhongdian, Yunnan, China, inferred from internal transcribed spacer sequence data. Chiang. Mai. J. Sci. 41, 811–821.

[B35] Hackel J. Henkel T. W. Moreau P.-A. Crop E. D. Verbeken A. Sà M.. (2022). Biogeographic history of a large clade of ectomycorrhizal fungi, the Russulaceae, in the Neotropics and adjacent regions. New Phytol. 236, 698–713. doi: 10.1111/nph.18365 35811430 PMC9795906

[B36] Hall T. A. (1999). BioEdit: a user-friendly biological sequence alignment editor and analyses program for windows 95/98/NT. Nucleic Acids Symp. Ser. 734, 95–98. doi: 10.1021/bk-1999-0734.ch008

[B37] Hampe F. Manz C. (2021). Two new *Russula* species from Thailand and the new subsection *Magicae* . Z. für Mykologie. 87, 17–30.

[B38] Hu H. Z. (2020). Investigation on edible and medicinal fungi and “Taimo” in Wutai Mountain (Changchun (Jilin: Jilin Agricultural University).

[B39] Jabeen S. Naseer R. Khalid A. N. (2020). *Russula rubricolor* sp. nov. from Himalayan forests of Pakistan. Mycotaxon 135, 763–774. doi: 10.5248/135.763

[B40] Jabeen S. Niazi A. R. Khalid A. N. (2017). *Russula brunneopurpurea* sp. nov. and its ectomycorrhiza from Pakistan. Mycosphere 8, 1059–1069. doi: 10.5943/mycosphere/8/8/7

[B41] Ji R. Q. Xie M. L. Zhou J. J. Meng L. P. Li Y. Zhang Z. H.. (2022). *Russula rubiginosus* sp. nov. in *Russula* subsect. *Maculatinae* from Heilongjiang Province, Northeast China. Phytotaxa 575, 140–148. doi: 10.11646/phytotaxa.575.2.3

[B42] Jiang X. M. (2017). The taxonomy of *Russula* subgenus *Russula* from China (Kunming (Yunan: Southwest Forestry University).

[B43] Jiang X. M. Li Y. K. Liang J. F. Wu J. R. (2017). Two new *Russula* species in China. J. Fujian. Agric. Forestry. Univ. (Natural. Sci. Edition). 46, 103–108. doi: 10.13323/j.cnki.j.fafu(nat.sci.).2017.01.016

[B44] Katanić M. Orlović S. Grebenc T. Bajc M. Pekeč S. Drekić M. (2019). Ectomycorrhizae of Norway spruce from its southernmost natural distribution range in Serbia. iForest 12, 43–50. doi: 10.3832/ifor2729-011

[B45] Katoh K. Standly D. M. (2016). A simple method to control over-alignment in the MAFFT multiple sequence alignment program. Bioinformatics 32, 1933–1342. doi: 10.1093/bioinformatics/btw108 27153688 PMC4920119

[B46] Khurshid R. Naseer A. Ahmad I. Khalid N. (2022). *Russula kashmiriana* sp. nov. in subgenus *Tenellula* from Himalayan temperate forests of Pakistan. Nord. J. Bot. 2022, e03644. doi: 10.1111/njb.03644

[B47] Kiran M. Caboň M. Senko D. Khalid A. N. Adamčík S. (2021). Description of the fifth new species of *Russula* subsect. *Maculatinae* from Pakistan indicates local diversity hotspot of ectomycorrhizal fungi in southwestern Himalayas. Life 11, 662. doi: 10.3390/life11070662 34357034 PMC8303804

[B48] Kirk P. M. Cannon P. F. Minter D. W. Stalpers J. A. (2008). Dictionary of the Fungi. 10th Edition (Wallingford: CAB International).

[B49] Kranabetter J. M. Friesen J. Gamiet S. Kroeger P. (2009). Epigeous fruiting bodies of ectomycorrhizal fungi as indicators of soil fertility and associated nitrogen status of boreal forests. Mycorrhiza 19, 535–548. doi: 10.1007/s00572-009-0255-0 19449039

[B50] Leonhardt T. Borovicka J. Sacky J. Santrucek J. Kamenik J. Kotrba P. (2019). Zn overaccumulating *Russula* species clade together and use the same mechanism for the detoxification of excess Zn. Chemosphere 225, 618–626. doi: 10.1016/j.chemosphere.2019.03.062 30901655

[B51] Li G. J. (2014). Taxonomy of *Russula* from China (Beijing: Institute of Microbiology, Chinese Academy of Sciences & University of Chinese Academy of Sciences).

[B52] Li G. J. Li S. F. Wen H. A. (2010). The *Russula* species resources and its economic values of China. Acta Edulis. Fungi. 17, 155–160.

[B53] Li G. J. Zhang C. L. Lin F. C. Zhao R. L. (2018a). Hypogeous gasteroid Lactarius sulphosmus sp. nov. and agaricoid *Russula vinosobrunneola* sp. nov. (Russulaceae) from China. Mycosphere 9, 838–858. doi: 10.5943/mycosphere/9/4/9

[B54] Li G. J. Zhang C. L. Zhao R. L. Lin F. C. (2018b). Two new species of Russula from Northeast China. Mycosphere 9, 431–443. doi: 10.5943/mycosphere/9/3/1

[B55] Li G. J. Zhao D. Li S. F. Yang H. J. Wen H. A. Liu X. Z. (2013). *Russula changbaiensis* sp. nov. from northeast China. Mycotaxon 124, 269–278. doi: 10.5248/124.269

[B56] Li G. J. Zhao R. L. Zhang C. L. Lin F. C. (2019). A preliminary DNA barcode selection for the genus *Russula* (Russulales, Basidio-mycota). Mycology 10, 61–74. doi: 10.1080/21501203.2018.1500400 31069120 PMC6493256

[B57] Li G. J. Liu T. Z. Li S. M. Zhao S. Y. Niu C. Y. Liu Z. Z.. (2023). Four new species of *Russula* subsection *Sardoninae* from China. J. Fungi. 9, 199. doi: 10.3390/jof9020199 PMC9963349 36836313

[B58] Li X. Y. (2021). Study on the species diversity and crude drug resources of *Russula* in Jilin Province. Jilin Agricultural University, Changchun (Jilin.

[B59] Li X. Y. Bau T. (2022). Three new records of *Russula* from Northeast China. J. Fungal Res. 20, 1–5. doi: 10.13341/j.jfr.2022.1403

[B60] Liddle A. R. (2007). Information criteria for astrophysical model selection. Mon. Not. R. Astron. Soc 377, 74–78. doi: 10.1111/j.1745-3933.2007.00306.x

[B61] Liu X. L. Bau T. Wang X. H. (2017). Species diversity of *russula* from the greater and lesser hinggan mountains in northeast China. Mycosystema 36, 1355–1368. doi: 10.13346/j.mycosystema.170015

[B62] Liu S. L. Wang X. W. Li G. J. Deng C. Y. Ross W. Leonardi M.. (2024). Fungal diversity notes 1717–1817: taxonomic and phylogenetic contributions on genera and species of fungal taxa. Fungal Divers. 124, 1–216. doi: 10.1007/s13225-023-00529-0

[B63] Liu Y. L. Whelen S. Hall B. D. (1999). Phylogenetic relationships among ascomycetes: evidence from an RNA polymerase II subunit. Mol. Biol. Evol. 16, 1799–1808. doi: 10.1093/oxfordjournals.molbev.a026092 10605121

[B64] Looney B. P. Meidl P. Piatek M. J. Miettinen O. Martin F. M. Matheny P. B.. (2018). Russulaceae: a new genomic dataset to study ecosystem function and evolutionary diversification of ectomycorrhizal fungi with their tree associates. New Phytol. 218, 54–65. doi: 10.1111/nph.15001 29381218

[B65] Looney B. P. Ryberg M. Hampe F. Sánchez-García M. Matheny P. B. (2016). Into and out of the tropics: global diversification patterns in a hyperdiverse clade of ectomycorrhizal fungi. Mol. Ecol. 25, 630–647. doi: 10.1111/mec.13506 26642189

[B66] Luo K. W. Yu X. L. Liu X. F. Li J. X. Huang H. Q. (2009). Vegetation investigation of tianmenshan national forest park. Hunan. Fore. Sci. Technol. 36, 37–41.

[B67] Malysheva E. F. Malysheva V. F. Kovalenko A. E. Pimenova E. A. Gromyko M. N. Bondarchuk S. N.. (2016). Below-ground ectomycorrhizal community structure in the postfire successional *Pinus koraiensis* forests in the central Sikhote-Alin (the Russian Far East). Botanica Pacifica. 5, 19–31. doi: 10.17581/bp.2016.05102

[B68] Matheny P. B. (2005). Improving phylogenetic inference of mushrooms with RPB1 and RPB2 nucleotide sequences (*Inocybe*, Agaricales). Mol. Phylogenet. Evol. 35, 1–20. doi: 10.1016/j.ympev.2004.11.014 15737578

[B69] Matheny P. B. Liu Y. J. Ammirati J. Hall B. (2002). Using RPB1 sequences to improve phylogenetic inference among mushrooms (*Inocybe*, Agaricales). Am. J. Bot. 89, 688–698. doi: 10.3732/ajb.89.4.688 21665669

[B70] Miller S. L. Buyck B. (2002). Molecular phylogeny of the genus Russula in Europe with a comparison of modern infrageneric clas-sifications. Mycol. Res. 106, 259–276. doi: 10.1017/S0953756202005610

[B71] Motiejūnaitė J. Kačergius A. Kasparavičius J. Taraškevičius R. Matulevičiūtė D. Iršėnaitė R. (2021). Response of ectomycorrhizal and other *Pinus sylvestris* root-associated fungi to the load of allochthonous material from a great cormorant colony. Mycorrhiza 31, 471–481. doi: 10.1007/s00572-021-01034-5 34101027

[B72] Mua A. Casula M. Sanna M. (2016). *Russula* rare o interessanti della Sardegna (Italia), 4. Rivista Micologica Romana. Bollettino. dell’. Associazione. Micol. Ecol. Romana. 97, 26–41.

[B73] Noffsinger C. R. Adamčíková K. Eberhardt U. Caboň M. Bazzicalupo A. Buyck B. (2024). Three new species in *Russula* subsection *Xerampelinae* supported by genealogical and phenotypic coherence. Mycologia 116, 322–349. doi: 10.1080/00275514.2023.2295957 38363178

[B74] Nylander J. A. A. (2004). MrModelTest v2. Program distributed by the author (Evolutionary Biology Center. (Biskopsgatan: Uppsala University).

[B75] Osmundson T. W. Robert V. A. Schoch C. L. Baker L. J. Smith A. Robich G.. (2013). Filling gaps in biodiversity knowledge for macrofungi: contributions and assessment of an herbarium collection DNA barcode sequencing project. PloS One 8, e62419. doi: 10.1371/journal.pone.0062419 23638077 PMC3640088

[B76] Palmer J. M. Lindner D. L. Volk T. J. (2008). Ectomycorrhizal characterization of an American chestnut (*Castanea dentata*)-dominated community in Western Wisconsin. Mycorrhiza 19, 27–36. doi: 10.1007/s00572-008-0200-7 18807258

[B77] Peck C. H. (1913). Report of the state botanist. 1912. Bull. New York. State. Museum. 167, 1–137.

[B78] Phookamsak R. Hyde K. D. Jeewon R. Bhat D. J. Jones E. B. G. Maharachchikumbura S. S. N.. (2019). Fungal diversity notes 929–1035: taxonomic and phylogenetic contributions on genera and species of fungi. Fungal Divers. 95, 1–273. doi: 10.1007/s13225-019-00421-w

[B79] Qi C. J. (1990). Vegetation of Hunan (Changsha: Hunan Science & Technology Press).

[B80] Rambaut A. Drummond A. J. Xie D. Baele G. Suchard M. (2018). A. Posterior summarization in bayesian phylogenetics using Tracer 1.7. Syst. Biol. 67, 901–904. doi: 10.1093/sysbio/syy032 29718447 PMC6101584

[B81] Rehner S. Buckley E. A. (2005). *Beauveria* phylogeny inferred from nuclear ITS and EF1-α sequences: evidence for cryptic diversification and links to *Cordyceps* teleomorphs. Mycologia 97, 84–98. doi: 10.1080/15572536.2006.11832842 16389960

[B82] Ridgway R. (1912). Color standards and color nomenclature (Washington: Robert Ridgway). Available at: http://www.columbia.edu/cu/lweb/digital/collections/cul/texts/ldpd_8627102_000/index.html.

[B83] Romagnesi H. (1985). Les Russules d’Europe et d’Afrique du Nord. Reprint with supplement (Lehre: J. Cramer).

[B84] Romell L. (1911). Hymenomycetes of lappland. Arkiv. før Botanik. 11, 1–35.

[B85] Ronquist F. Huelsenbeck J. P. (2003). MRBAYES 3: Bayesian phylogenetic inference under mixed models. Bioinformatics 19, 1572–1574. doi: 10.1093/bioinformatics/btg180 12912839

[B86] Ronquist F. Teslenko. M. van der Mark P. Ayres D. D. A. Höhna S. Larget B.. (2012). MrBayes 3.2: Efficient Bayesian phylogenetic inference and model choice across a large model space. Syst. Biol. 61, 539–542. doi: 10.1093/sysbio/sys029 22357727 PMC3329765

[B87] Rosenblad M. A. Martín M. P. Ryberg M. Larsson E. Wurzbacher C. Abarenkov K.. (2016). Detection of signal recognition particle (SRP) RNAs in the nuclear ribosomal internal transcribed spacer I (ITSI) of three lineages of ectomycorrhizal fungi (Agaricomycetes, Basidiomycota). MycoKeys 13, 21–33. doi: 10.3897/mycokeys.13.8579

[B88] Ruotsalainen J. Vauras J. (1990). Finnish records of the genus *Russula.* The new species *R. olivina* and *R. taigarum* . Karstenia 30, 15–26. doi: 10.29203/ka.1990.277

[B89] Saba M. Khalid A. N. (2015). *Russula sichuanensis* and its ectomycorrhizae from Himalayan moist temperate forests of Pakistan. Mycotaxon 130, 629–639. doi: 10.5248/130.629

[B90] Sarnari M. (1998). Monografia illustrate del genere *Russula* in Europa; Tromo Primo (Trento: AMB, Centro Studi Micologici).

[B91] Sarnari M. (2005). Monografia illustrate del genere *Russula* in Europa; Tromo Secondo (Trento: AMB, Centro Studi Micologici).

[B92] Schoch C. L. Seifert K. A. Huhndorf S. Robert V. Spouge J. L. Levesque C. A.. (2012). Nuclear ribosomal internal transcribed spacer (ITS) region as a universal DNA barcode marker for Fungi. Proc. Natl. Acad. Sci. U. S. A. 109, 6241–6246. doi: 10.1073/pnas.1117018109 22454494 PMC3341068

[B93] Shi L. Y. (2021). Species diversity of macrofungi in northern Taihang Mountains, Hebei Province and mycelium research of *Phellinus orientoasiaticus* . Hebei Agricultural University, Baoding (Hebei.

[B94] Shirakawa M. Ishikawa A. Fuchigami T. Tanaka M. (2022). Assessment of ectomycorrhizal fungal diversity in a suburban secondary forest in the northwestern part of Tama Area, Tokyo. J. Jpn. For. Soc 104, 351–362. doi: 10.4005/jjfs.104.351

[B95] Singer R. (1938). Contribution à l'étude des Russules (1)-3. Quelques Russules américaines et asiatiques. Bull. la. Société. Mycol. France. 54, 132–177.

[B96] Singer R. (1986). The Agaricales in Modern Taxonomy (Koenigstein: Koeltz Scientific Books).

[B97] Siquier J. L. Salom J. C. Espinosa J. Esteve- Raventós F. Llistosella J. Gomes S. (2015). Contribució al coneixement micològic de les Illes Balears (Espanya). XXI. Rev. Catalana. Micol. 36, 59–88.

[B98] Sleiman S. Bellanger J. M. Richard F. Stephan J. (2021). First molecular-based contribution to the checklist of Lebanon macrofungi. Mycotaxon 136, 687–687. doi: 10.5248/136.687

[B99] Smith A. H. (1963). New astrogastraceous fungi from the Pacific Northwest. Mycologia 55, 421–441. doi: 10.1080/00275514.1963.12018037

[B100] Song B. Li T. H. Wu X. L. Li J. J. Shen Y. H. Lin Q. Y. (2007). Known species of *Russula* from China and their distribution. J. Fungal Res. 5, 20–42. doi: 10.13341/j.jfr.2007.01.007

[B101] Song Y. Xie X. C. Buyck B. (2021). Two novel species of subgenus *Russula* crown clade (Russulales, Basidiomycota) from China. Eur. J. Taxonomy. 775, 15–33. doi: 10.5852/ejt.2021.775.1543

[B102] Suz L. M. Barsoum N. Benham S. Dietrich H. P. Fetzer K. D. Fischer R.. (2014). Environmental drivers of ectomycorrhizal communities in Europe’s temperate oak forests. Mol. Ecol. 23, 5628–5644. doi: 10.1111/mec.12947 25277863

[B103] Swofford D. L. (2004). PAUP*: phylogenetic analysis using parsimony and other methods. Version4.0b10 (Sunderland: Sinauer).

[B104] Trendel J. M. (2021). *Russula pseudochamaeleontina* sp. nov. Une nouvelle russule de la sous-section *Chamaeleontinae* . Bull. Soc Mycol. Strasbg. 120, 3–10.

[B105] Vaidya G. Lohman D. J. Meier R. (2011). SequenceMatrix: concatenation software for the fast assembly of multi-gene datasets with character set and codon information. Cladistics 27, 171–180. doi: 10.1111/j.1096-0031.2010.00329.x 34875773

[B106] Vauras J. Ruotsalainen J. Liimatainen K. (2012). *Russula graminea*, a new green species from Fennoscandia. Karstenia 52, 51–57. doi: 10.29203/ka.2012.449

[B107] Vidal J. M. Alvarado P. Loizides M. Konstantinidis G. Chachuła P. Mleczko P.. (2019). A phylogenetic and taxonomic revision of sequestrate Russulaceae in Mediterranean and temperate Europe. Persoonia 42, 127–185. doi: 10.3767/persoonia.2019.42.06 31551617 PMC6712534

[B108] Vidal J. M. Calonge F. D. Martín M. P. (2002). *Macowanites ammophilus* (Russulales) a new combination based on new evidence. Rev. Catalana. Mycol. 24, 69–74.

[B109] Vilgalys R. Hester M. (1990). Rapid genetic identification and mapping of enzymatically amplified ribosomal DNA from several *Cryptococcus* species. J. Bacteriol. 172, 4238–4246. doi: 10.1128/jb.172.8.4238-4246.1990 2376561 PMC213247

[B110] Wang J. (2019). Taxonomy of edible species of *Russula* in Yunnan. Jilin Agricultural University, Changchun (Jilin.

[B111] Wang X. H. (2020). Taxonomic comments on edible species of Russulaceae. Mycosystema 39, 1617–1639. doi: 10.13346/j.mycosystema.200209

[B112] Wang X. H. Das K. Bera I. Chen Y. H. Bhatt R. P. Ghosh. A.. (2019). Fungal biodiversity profiles 81–90. Cryptogam. Mycol. 40, 57–95. doi: 10.5252/cryptogamie-mycologie2019v40a5

[B113] Wang L. Dong L. Zhao Z. P. Lu S. Z. Wang J. Liu Y. G.. (2021). Vegetation diversity and mapping in the priority area of Taihang Mountains biodiversity conservation (Beijing-Tianjin-Hebei region). Sci. Sin. Vitae. 51, 289–299. doi: 10.1360/SSV-2020-0174

[B114] Wang K. Du Z. Guo Y. B. Liu T. Z. Xie M. L. Zhao M. J.. (2024). Diversity of macrofungi in taihang mountains of Beijing and Hebei, North China. Mycosystema 43, 230311. doi: 10.13346/j.mycosystema.230311

[B115] Wang X. B. Liu J. J. Long D. F. Han Q. S. Jiang H. (2017). The ectomycorrhizal fungal communities associated with *Quercus liaotungensis* in different habitats across northern China. Mycorrhiza 27, 441–449. doi: 10.1007/s00572-017-0762-3 28120112

[B116] Wang X. H. Yang Z. L. Li Y. C. Knudsen H. Liu P. G. (2009). *Russula griseocarnosa* sp. nov. (Russulaceae, Russulales), a commercially important edible mushroom in tropical China: mycorrhiza, phylogenetic position, and taxonomy. Nova. Hedwigia. 88, 269–282. doi: 10.1127/0029-5035/2009/0088-0269

[B117] Wang J. Zeng W. J. Zheng X. Wang W. K. Zhu Y. M. Yang Y. H. (2021). *Russula abietiphila*, a new record of *Russula* subg. in China. J. West. China Forestry. Sci. 50, 113–119. doi: 10.16473/j.cnki.xblykx1972.2021.05.016

[B118] Wei J. Gao W. Huang C. Y. (2021). A checklist of edible ectomycorrhizal mushrooms in China. Mycosystema 40, 1938–1957. doi: 10.13346/j.mycosystema.210031

[B119] Wei T. Z. Liu T. Z. (2019). Resource survey of Macro-Basidiomycetes in southern Greater Khingan Mountains, Chifeng City. J. Liaocheng. Univeristy. (Nat. Sci. ) 32, 76–89. doi: 10.19728/j.issn1672-6634.2019.06.012

[B120] Whitbeck K. L. (2003). Systematics of Pacific Northwestern species of the genus *Gymnomyces* inferred from nuclear ribosomal DNA internal transcribed spacer sequences. Oregon State University, Corvallis (Oregon.

[B121] White T. J. Bruns T. Lee S. Taylor J. (1990). “Amplification and direct sequencing of fungal ribosomal RNA genes for phylogenies,” in PCR protocols, a guide to methods and applications. Eds. Innis M. A. Gelfand D. H. Sninsky J. J. White T. J. (Academic, San Diego), 315–322. doi: 10.1016/B978-0-12-372180-8.50042-1

[B122] Xu W. J. Qiao P. Wan S. P. Gong H. D. Yu F. Q. (2019). New record of *Macowanites* in China. J. West. China For. Sci. 48, 137–140. doi: 10.16473/j.cnki.xblykx1972.2019.03.022

[B123] Yang S. Pfister D. (2006). *Monotropa uniflora* plants of eastern Massachusetts form mycorrhizae with a diversity of russulacean fungi. Mycologia 98, 535–540. doi: 10.1080/15572536.2006.11832656 17139846

[B124] Zhou H. Cheng G. Q. Wang Q. T. Zhuo L. Yan H. F. Li G. J.. (2022a). Morphological characteristics and phylogeny reveal six new species in *Russula* subgenus *Russula* (Russulaceae, Russulales) from Yanshan Mountains, North China. J. Fungi. 8, 1283. doi: 10.3390/jof8121283 PMC9785408 36547616

[B125] Zhou H. Shen X. Y. Hou C. L. (2023). A new species of *Russula* subgenus *Russula* (Russulaceae, Russulales) from Yanshan Mountains, North China. Phytotaxa 609, 195–208. doi: 10.11646/phytotaxa.609.3.3 PMC9785408 36547616

[B126] Zhou J. J. Xie M. L. Li G. J. Song J. Abdula D. Xing P. J.. (2022b). *Russula quercina*, a new species of *Russula* subsect. *Integroidinae* (Russulaceae, Russulales) from the *Quercus mongolica* forest in Heilongjiang Province, China. Phytotaxa 549, 77–86. doi: 10.11646/phytotaxa.549.1.6

