# Supplementary figures and images for: Multi-gene analysis of the Russula crown clade (Russulales, Basidiomycota) revealed six new species and Alboflavinae subsect. nov. from Fagaceae forests in China
Source: Front Plant Sci. 2024 Oct 7;15:1454035. doi: 10.3389/fpls.2024.1454035 (PMC11494609; doi:10.3389/fpls.2024.1454035)

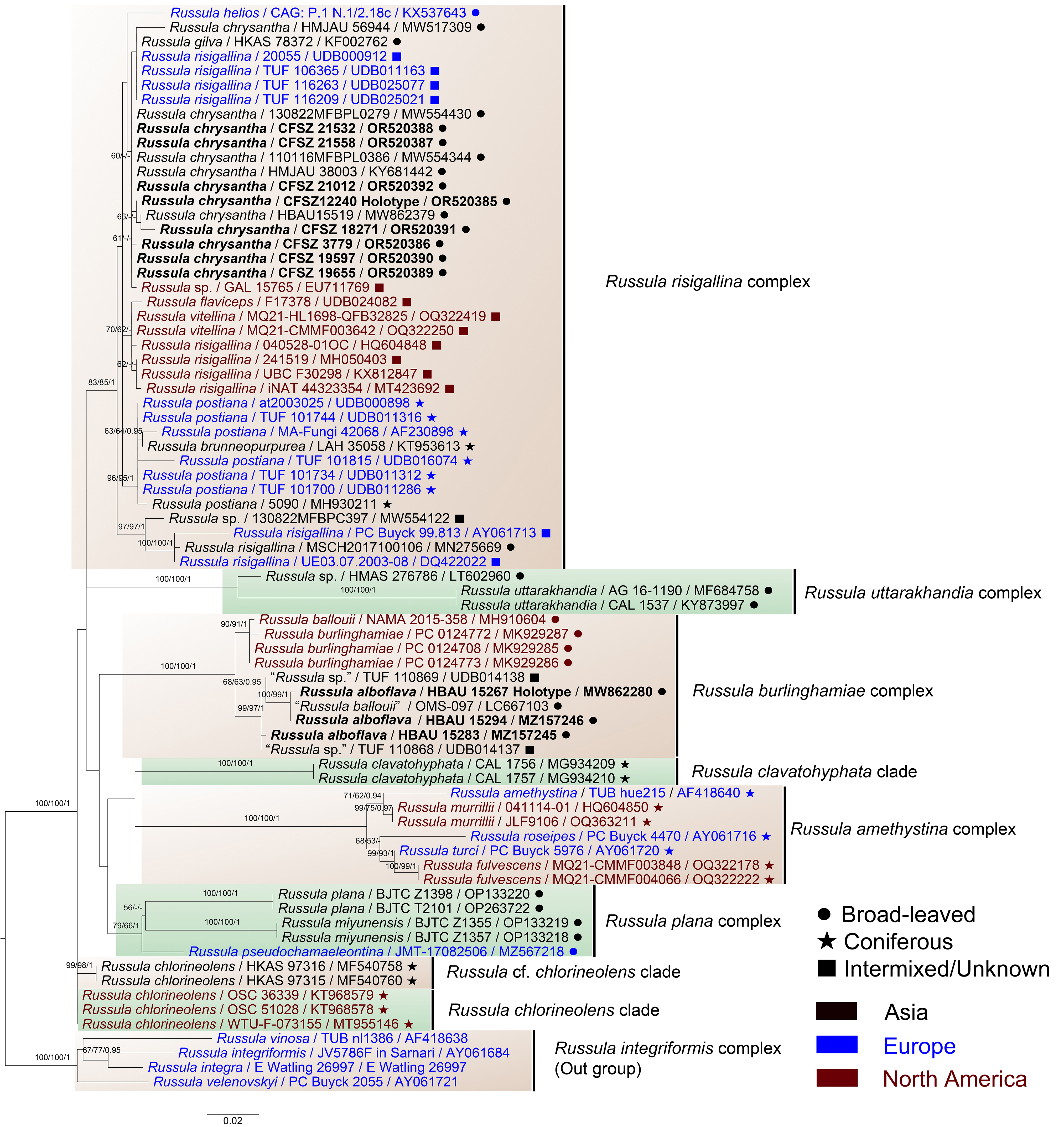

Supplement: Supplementary Figure 1 — Maximum likelihood (ML) phylogenetic tree of sect. Amethystinae based on ITS region. Bootstrap values ≥75% of ML and MP, as well as posterior probabilities ≥0.9 are presented above the clades as MLBS/MPBS/PP. Labels in bold represented new collections for this analysis. [file Image1.jpg]

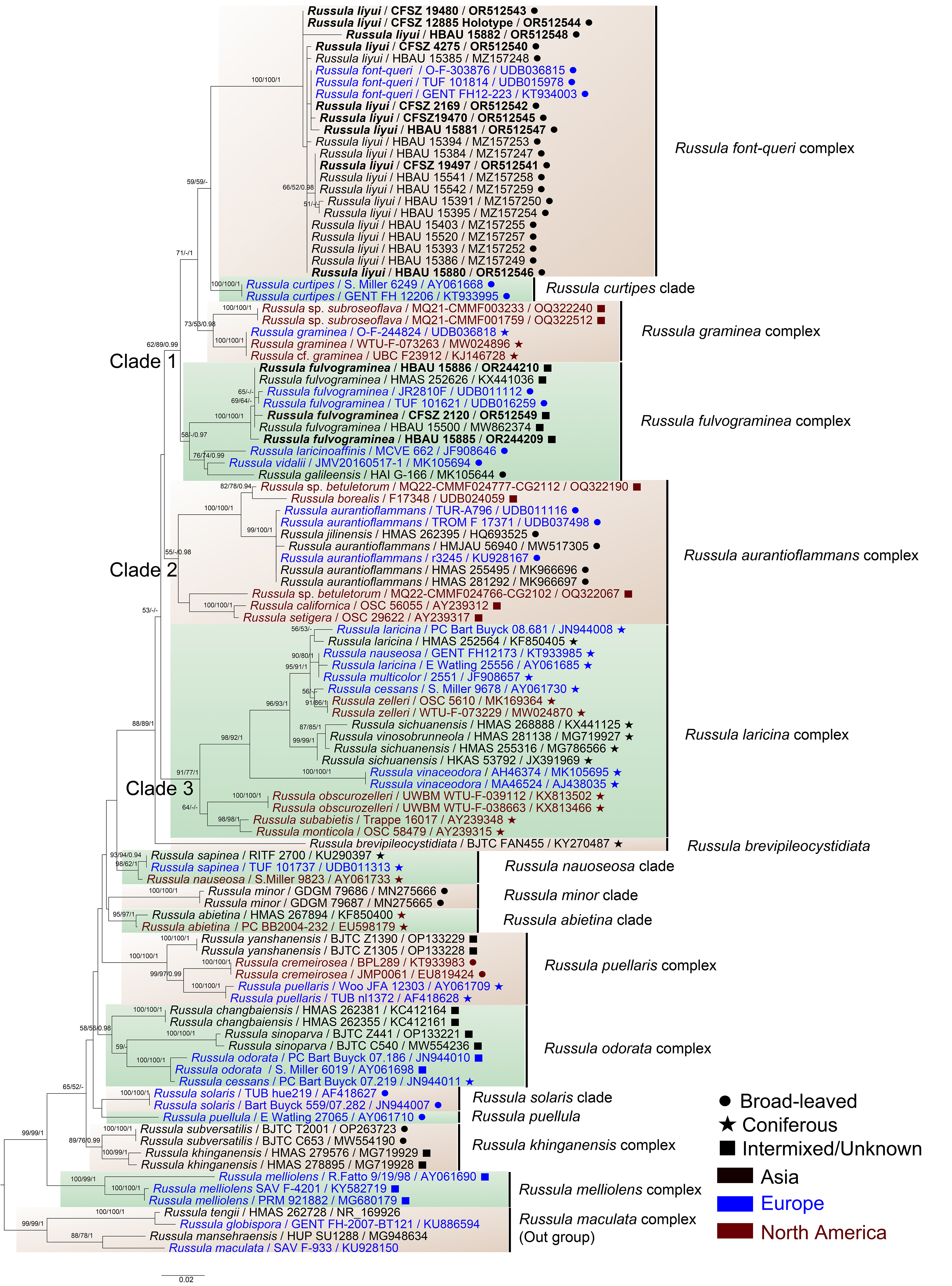

Supplement: Supplementary Figure 2 — Maximum likelihood (ML) phylogenetic tree of subsect. Laricinae based on ITS region. Bootstrap values ≥75% of ML and MP, as well as posterior probabilities ≥ 0.9 are presented above the clades as MLBS/MPBS/PP. Labels in bold represented new collections for this analysis. [file Image2.jpg]

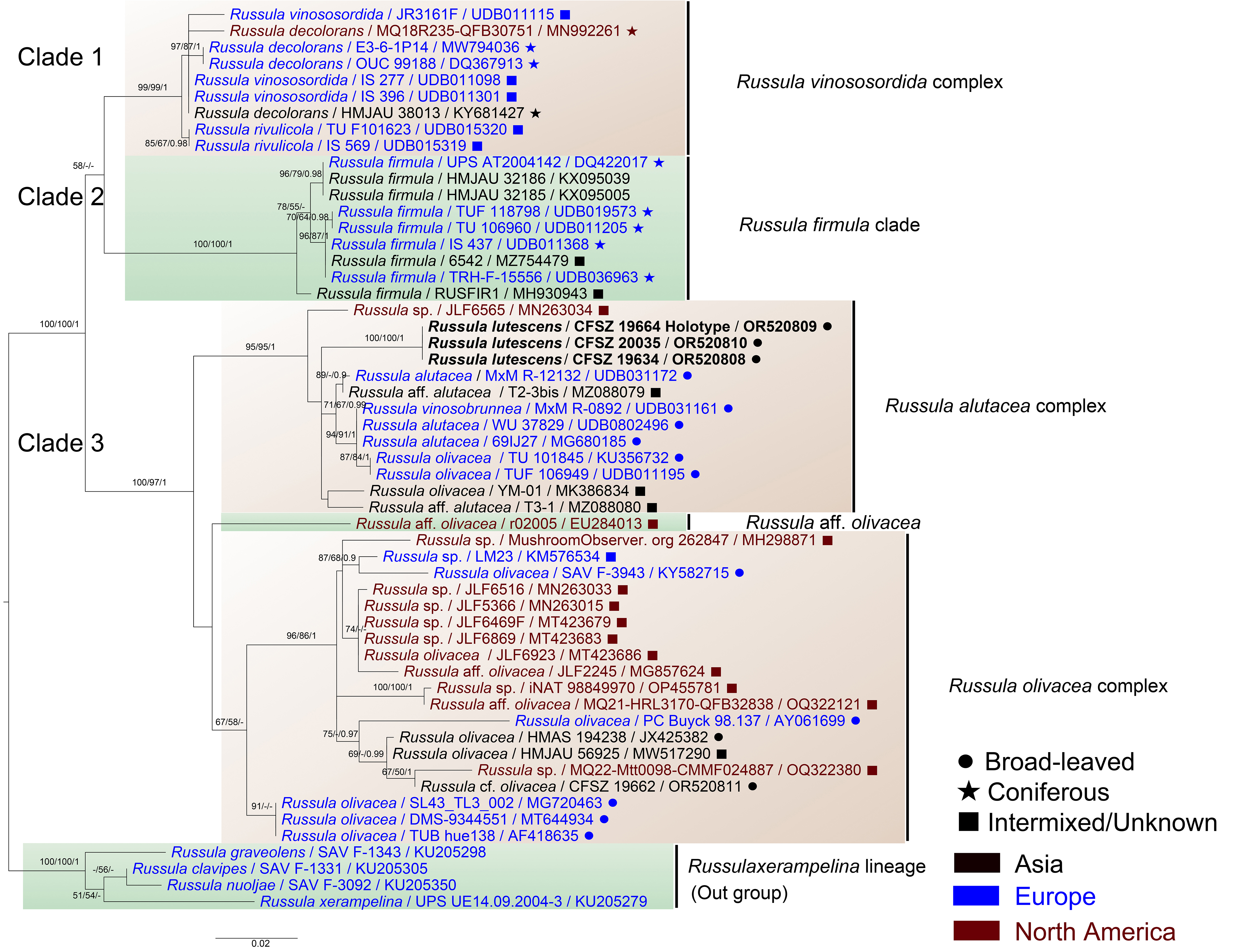

Supplement: Supplementary Figure 3 — Maximum likelihood (ML) phylogenetic tree of subsect. Olivaceinae based on ITS region. Bootstrap values ≥75% of ML and MP, as well as posterior probabilities ≥0.9 are presented above the clades as MLBS/MPBS/PP. Labels in bold represented new collections for this analysis. [file Image3.jpg]

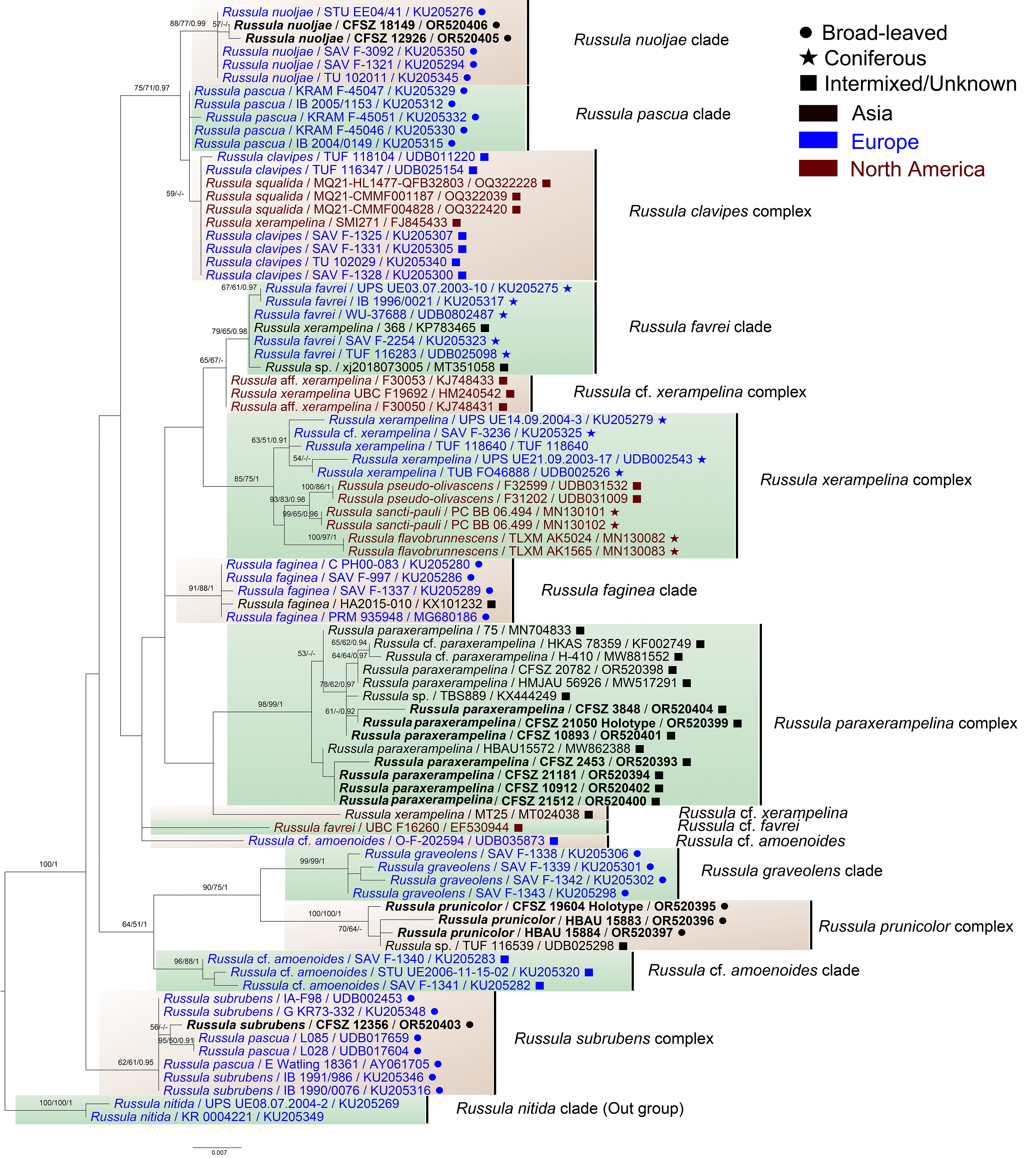

Supplement: Supplementary Figure 4 — Maximum likelihood (ML) phylogenetic tree of subsect. Xerampelinae based on ITS region. Bootstrap values ≥75% of ML and MP, as well as posterior probabilities ≥ 0.9 are presented above the clades as MLBS/MPBS/PP. Labels in bold represented new collections for this analysis. [file Image4.jpg]
